# Freestanding Metal–Organic Frameworks and Their Derivatives: An Emerging Platform for Electrochemical Energy Storage and Conversion

**DOI:** 10.1021/acs.chemrev.1c00978

**Published:** 2022-04-21

**Authors:** Bing He, Qichong Zhang, Zhenghui Pan, Lei Li, Chaowei Li, Ying Ling, Zhixun Wang, Mengxiao Chen, Zhe Wang, Yagang Yao, Qingwen Li, Litao Sun, John Wang, Lei Wei

**Affiliations:** † School of Electrical and Electronic Engineering, Nanyang Technological University, 50 Nanyang Avenue, Singapore 639798, Singapore; ‡ Key Laboratory of Multifunctional Nanomaterials and Smart Systems, Suzhou Institute of Nano-Tech and Nano-Bionics, Chinese Academy of Sciences, Suzhou 215123, China; § Department of Materials Science and Engineering, National University of Singapore, Singapore 117574 Singapore; ∥ SEU-FEI Nano-Pico Center, Key Laboratory of MEMS of Ministry of Education, Southeast University, Nanjing 210096, China; ⊥ Division of Nanomaterials and Jiangxi Key Lab of Carbonene Materials, Jiangxi Institute of Nanotechnology, Nanchang 330200, China; # College of Engineering and Applied Sciences and Collaborative Innovation Center of Advanced Microstructures, Nanjing University, Nanjing 210093, China; ∇ College of Biomedical Engineering and Instrument Science, Zhejiang University, Hangzhou 310027, China; ◊ Henan Key Laboratory of New Optoelectronic Functional Materials, College of Chemistry and Chemical Engineering, Anyang Normal University, 436 Xian’ge Road, Anyang 455000, China; ○ Institute of Materials Research and Engineering, A*Star, Singapore 138634, Singapore

## Abstract

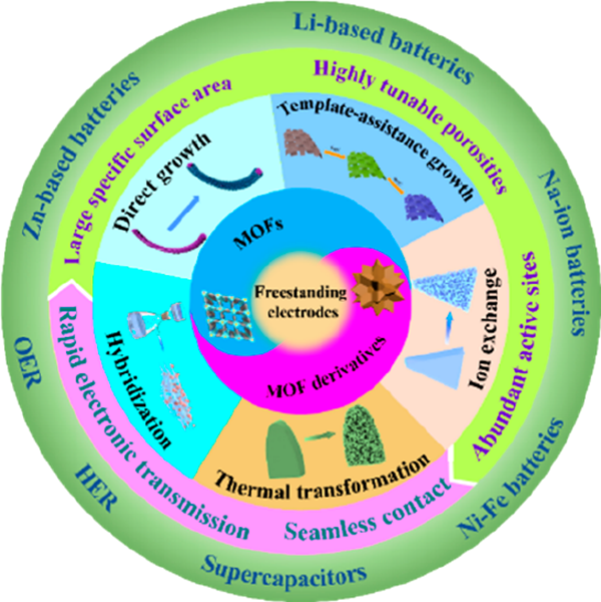

Metal–organic frameworks (MOFs) have recently emerged as ideal electrode materials and precursors for electrochemical energy storage and conversion (EESC) owing to their large specific surface areas, highly tunable porosities, abundant active sites, and diversified choices of metal nodes and organic linkers. Both MOF-based and MOF-derived materials in powder form have been widely investigated in relation to their synthesis methods, structure and morphology controls, and performance advantages in targeted applications. However, to engage them for energy applications, both binders and additives would be required to form postprocessed electrodes, fundamentally eliminating some of the active sites and thus degrading the superior effects of the MOF-based/derived materials. The advancement of freestanding electrodes provides a new promising platform for MOF-based/derived materials in EESC thanks to their apparent merits, including fast electron/charge transmission and seamless contact between active materials and current collectors. Benefiting from the synergistic effect of freestanding structures and MOF-based/derived materials, outstanding electrochemical performance in EESC can be achieved, stimulating the increasing enthusiasm in recent years. This review provides a timely and comprehensive overview on the structural features and fabrication techniques of freestanding MOF-based/derived electrodes. Then, the latest advances in freestanding MOF-based/derived electrodes are summarized from electrochemical energy storage devices to electrocatalysis. Finally, insights into the currently faced challenges and further perspectives on these feasible solutions of freestanding MOF-based/derived electrodes for EESC are discussed, aiming at providing a new set of guidance to promote their further development in scale-up production and commercial applications.

## 1 Introduction

With the rapid rise in demand for energy consumption and climate change, developing renewable energy technologies has been an imminent task to address the severe energy and environmental issues resulting from the excessive use of fossil fuels (e.g., coal, oil, natural gas, etc.).^1−3^ In recent years, there have been widespread efforts in developing new electrochemical energy storage and conversion (EESC) technologies, which have stimulated an extensive range of research enthusiasm into various key energy materials and devices.^4−8^ Among the EESC systems are rechargeable batteries, supercapacitors, and electrocatalysis, which effectively store and convert the different types of energy, including intermittent clean energy (wind, solar, etc.). Both batteries and supercapacitors are at the forefront of energy storage, where advanced electrodes with high specific capacity and durability are inevitably needed,^9−11^ while electrocatalysis is the dominating energy conversion process in which functional electrocatalysts with high activity, stability, and selectivity are critically important.^12−14^ Although relying on different working mechanisms, both the energy storage and the conversion systems possess a fundamental pursuit for the physical and chemical properties of electrode materials, consisting of a large accessible specific surface, high electrical/ionic conductivity, abundant reactive sites, long-term structure stability, etc., which play essential roles in the overall performance of EESC devices.^15−17^ Consequently, the exploration of better performing materials with optimal architectures and appropriate functionalities has always been the primary pathway to the development/breakthrough of future EESC technologies.

As a new class of crystalline porous materials, metal–organic frameworks (MOFs), also denoted as porous coordination polymers (PCPs), consist of metal ions/clusters and organic ligands with long-range ordered structures, which have been studied and developed rapidly in EESC in recent years.^18−26^ In particular, among their most fascinating features is that the framework could be designed by a modular self-assembly process and thus be beneficial for achieving the versatility of MOFs suitable for various applications, including energy storage,^27,28^ electrocatalysis,^29,30^ selective gas adsorption and separation,^31,32^ hydrogen storage,^33,34^ and even drug delivery.^35,36^ MOF-based/derived materials have received increasing attention more recently owing to their highly tunable and ordered structures, controllable level of porosities, and rich physical and chemical properties. As a result, developing MOF-based/derived materials for EESC applications has been witnessed by a large number of research publications in the past decades, covering electrochemistry, materials chemistry, materials engineering, and their synthesis and fabrications.^37−46^ Despite the significant progress achieved, these MOF-based/derived materials are primarily fabricated in various powder forms, and the common slurry-processed powder electrodes generally contain binders and additives, fundamentally eliminating some of the active sites and thus degrading the performance of the MOF-based/derived materials.^47−49^ Undoubtedly, developing freestanding electrodes would not only simplify the fabrication process and eliminate the undesirable interfaces produced by additional binders/additives but also provide a larger surface area, more abundant accessible active sites, and much enhanced charge transfer efficiency.^48,50−53^ Therefore, the developments of freestanding MOF-based/derived electrodes have brought new yet promising opportunities into EESC technologies. As freestanding MOFs and their derivatives are among the most exciting and rapidly ongoing topics, it would be critical and timely to examine the latest development in this field, providing the much needed guidance for the design and fabrication of freestanding MOF-based/derived electrodes for future EESC applications.

This review will summarize the material design strategies and fabrication methods, showing the diversification of the chemical components and crystal structures of freestanding MOF-based/derived electrodes. The recent advances in the composition/structure/performance relationship will then be highlighted, and their applications in electrochemical energy storage and electrocatalysis will be presented in detail. Finally, based on the current developments, insights into the present challenges and the perspectives on these feasible solutions of freestanding MOF-based/derived electrodes for EESC will be discussed, aiming to provide a new set of guidelines on the future development of the highly promising field.

## 2 Structural Features and Superiority of Freestanding MOF-Based/Derived Electrodes

### 2.1 Freestanding Electrodes

**Figure 1:**
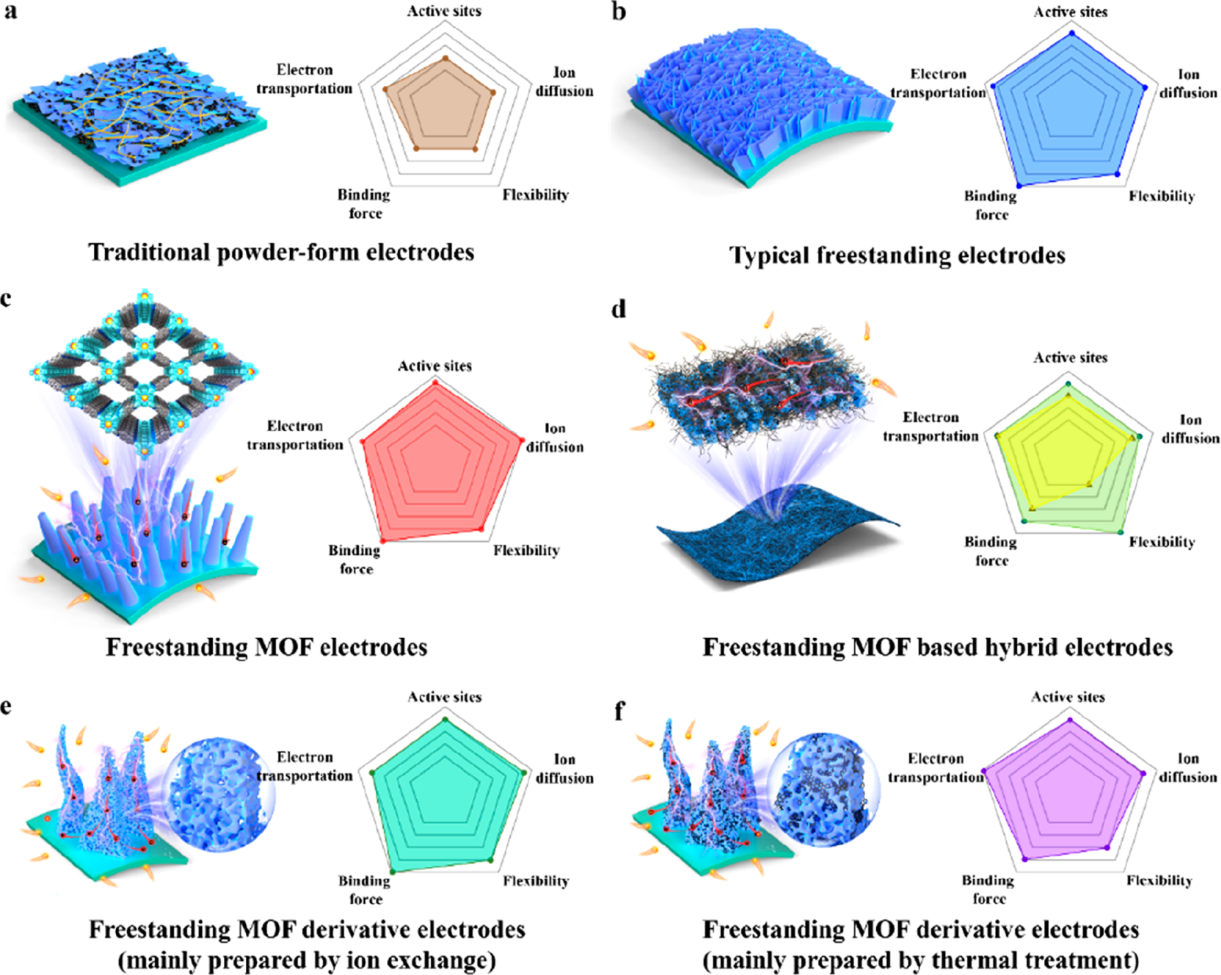
Principle and feature comparison of (a) traditional powder-form electrodes and (b) typical freestanding electrodes. Schematic diagrams and corresponding features of freestanding MOF-based electrodes for EESC: (c) freestanding MOF-based electrodes, (d) freestanding MOF-hybrid electrodes (green mainly represents the electrodes prepared by electrospinning and filtration, while yellow represents the electrodes fabricated by 3D printing), and freestanding MOF-derived electrodes prepared by (e) ion exchange and (f) thermal treatment.

For EESC, the construction of appropriate electrodes plays a determining role in achieving the expected electrochemical performance. Among various fabrication strategies, the slurry-casting method is a common way to prepare the conventional powder-form electrodes by mixing active materials, conductive carbon, and nonconductive binders and then coating them on conductive substrates, as illustrated in Figure 1a. Despite the fact that the slurry-casting method is widely adopted in industrial applications, introduction of any additives would not only bury the active sites resulting in low electrochemical capacitances/capacities or catalytic activities but also impede the continuous transport of electrons/charges and effective diffusion of ions leading to poor electrical conductivity and sluggish kinetics.^54,55^ In addition, the weak binding force between active materials and conductive substrates can severely limit their life spans in diversified applications, such as when they are acting as the power sources for wearable electronics.^54,56^ In comparison, the development of robust freestanding electrodes provides a promising pathway to overcome these limitations caused by the use of binders, which are the “dead” mass, and the undesirable interfaces.^47,55,57^Figure 1b displays the typical structural schematic of freestanding electrodes in which active materials are directly grown on the surface of substrates without any binders, exhibiting numerous merits in EESC. First, direct growth of active materials on substrates avoids the addition of conductive agents and binders, which adequately exposes more active sites and simplifies the manufacturing process of electrodes. Second, formation of nanoarrays effectively prevents the aggregation of nanostructured active materials, and the tunable space among nanoarrays achieves the rapid diffusion of electrolyte ions and the accommodation of volume expansion during electrochemical reactions, especially for energy storage. Third, the seamless contact between the active materials and conductive substrates enhances the electrical conductivity of electrodes while forming a strong binding force for stable electrochemical reactions. Finally, compared with the powder-form electrodes with limited conductive substrates, freestanding electrodes enable various choices in substrates, endowing them with unique features, including flexibility, being lightweight, and having stitchability. As a result, these advantages can fundamentally boost the electrochemical capacities/capacitance, rate capabilities, and catalytic activities of electrode materials for EESC.

### 2.2 Freestanding MOF-Based/Derived Electrodes

As mentioned in section 2.1, freestanding electrodes provide a promising platform for high-performance EESC owing to their intriguing structural features. MOF-based/derived materials possess high porosity and excellent specific surface areas composed of a large number of organic ligands and metal ions, which are very conducive to the rapid transfer of ions and accessibility of active sites.^58−62^ Therefore, the combination of freestanding structures and MOFs is expected to generate a number of synergistic effects to achieve superior electrochemical performance in EESC. To date, based on the compositions and structures, freestanding MOF-based/derived electrodes can mainly be divided into three categories: including freestanding MOF-based electrodes (Figure 1c), freestanding MOF–hybrid electrodes (Figure 1d), and freestanding MOF-derived electrodes (Figure 1e and 1f). It is worth mentioning that the preparation strategies of freestanding electrodes have significant effects on the structure and performance. Therefore, compared with conventional powder-form MOF-based/derived electrodes, the freestanding MOF-based/derived electrodes exhibit the following merits. (i) Direct formation of electrodes, where the as-obtained freestanding MOF-based/derived materials can directly serve as binder-free electrodes for EESC, which not only simplifies the preparation process of the electrodes but also benefits in achieving a uniform distribution of active materials. (ii) Remarkable electron transfer kinetics. Due to the absence of inactive polymer binders, freestanding electrodes exhibit seamless contact between active materials and conductive substrates, which is conducive to the fast transportation of electrons. In particular, the application of most of the pristine MOFs in EESC is generally limited by their poor electrical conductivity. Therefore, the combination of freestanding architectures and MOF-based materials could address the above issues and accordingly make use of the excellent ion diffusion property of the latter with abundant porous structures. (iii) Abundant reactive sites. Benefiting from the large specific surface area of MOF-based materials and the additive-free design of freestanding electrodes, the target electrodes would offer more active sites and be expected to achieve higher electrochemical performance. Besides, in the energy storage devices, the elimination of “dead mass”, typically greater than 20% in traditional powder-form MOF-based/derived electrodes, also effectively improves the volumetric/mass energy and power densities. (iv) Good cycle stability. For energy storage, enough free space shall accommodate the volume expansion of active materials during the charging/discharging processes to restrain the capacity decay. For energy conversion, open array structures and strong adhesion with substrates could effectively facilitate the fast diffusion of bubbles and suppress the exfoliation of active materials in the catalytic reactions. (v) Diversified selections of conductive substrates. Compared to the powder-form MOF-based/derived materials, the freestanding MOF-based/derived electrode possesses a broader selection range of conductive substrates, from 1D to 3D, such as carbon nanotube fibers (CNTF), carbon clothes (CC), carbon papers, metallic foils, carbon-based aerogels, and nickel/copper foams (NF/CF), endowing electrodes with unique characteristics including being lightweight and low cost and having a small volume, remarkable flexibility, and stitchability.

## 3 Design and Fabrication of Freestanding MOFs and Their Derivatives

Rational design and synthesis strategies could endow freestanding MOF-based/derived electrodes with desirable structural features, further achieving promising electrochemical performance. For freestanding MOFs, their preparations are primarily dependent on selecting the appropriate metal sources, organic linkers, solvents, and substrates, while the fabrication of freestanding MOF derivatives is related to the post-treatment processes, such as thermal treatment and ion exchanges. To date, a number of strategies have been adopted to construct freestanding MOF-based electrodes, and they are summarized in terms of MOFs and MOF derivatives in the following sections.

### 3.1 Preparation of Freestanding MOFs

#### 3.1.1 Direct Growth

**Figure 2:**
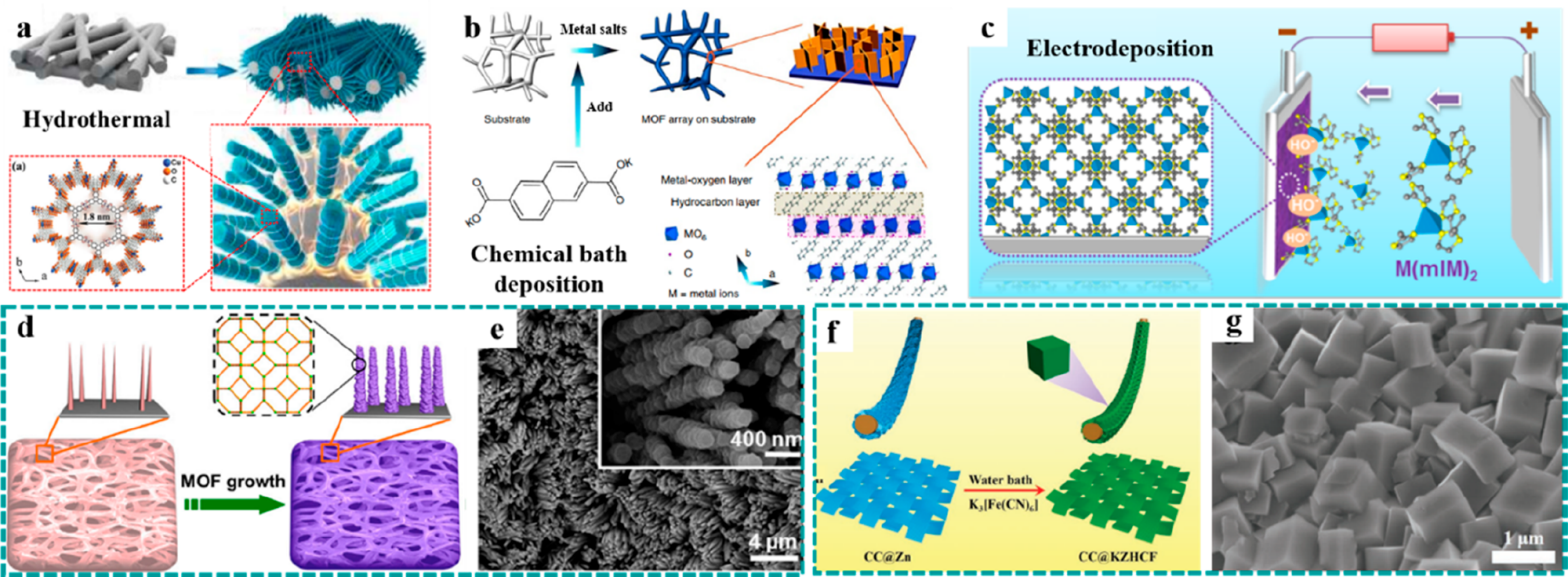
Synthesis strategies of freestanding MOFs. Direct growth: preparation schematic diagrams of (a) Cu–CAT nanorod arrays grown on CFP by a hydrothermal method. Reproduced with permission from ref (75). Copyright 2021 Wiley-VCH. (b) Ultrathin NiFe–MOF nanosheet arrays synthesized on NF by chemical bath deposition. Reproduced with permission from ref (77). Copyright 2017 The Author(s). (c) ZIF films grown on various substrates by an electrodeposition method. Reproduced with permission from ref (81). Copyright 2018 American Chemical Society. Template-assistance growth: preparation flowcharts and SEM images of (d and e) Ni@CoO@ZIF-67 (Reproduced with permission from ref (82). Copyright 2017 Elsevier.) and (f and g) CC@KZHCF (Reproduced with permission from ref (85). Copyright 2019 Wiley-VCH.).

Preparations of freestanding MOFs can be classified into three categories, namely, direct growth, template-assistance growth, and hybridization. Direct growth, just as its name implies, is to grow active materials on the surface of selected conductive substrates (e.g., NF, CC, conductive glass, Cu foil, CNTF, etc.) by a one-step method,^63−67^ consisting of the hydro/solvothermal method, chemical bath deposition, and electrodeposition.^68−72^ In this strategy, the target conductive substrates are immersed into a reaction solution, where MOF arrays are grown on the surface of conductive substrates at a specific temperature through a liquid–solid growth mechanism. Thus, the hydro/solvothermal method is a common and effective means to synthesize MOFs with various morphologies on the substrates in a pressure-tight reactor within a wide temperature range of 100–200 °C.^63,73,74^ During the synthetic process, the surface defects and oxyl groups on the surface of conductive substrates provide accessible sites to achieve the nucleation and conformal growth of MOFs, which are beneficial to the uniform distribution of MOFs and the enhancement of the binding force between MOFs and substrates.^54^ Moreover, the strategy favors the oriented growth of crystalline materials and thus could effectively construct various nanostructured MOF arrays, exposing larger specific surfaces and accessible active sites for better electrochemical performance. For example, the conductive Cu–CAT MOF nanowire arrays were grown on carbon fiber paper (CFP) by a one-step hydrothermal method (Figure 2a), directly acting as freestanding electrodes without conductive additives and binders.^72^ The Cu–CAT nanowire arrays exhibited a high level of porosity and excellent conductivity, leading to an outstanding capacitive performance. Compared to the hydro/solvothermal method, chemical bath deposition is a more facial strategy to grow MOFs on the surface of conductive substrates at a low temperature (<100 °C) in an ambient atmosphere, which has a similar synthetic mechanism as the former. Thus, this strategy is frequently adopted to prepare MOF nanostructured arrays on conductive substrates.^76,77^ For example, Zhao et al. developed a simple one-step chemical bath deposition method to synthesize ultrathin NiFe–MOF nanosheet arrays with a thickness of ∼3.5 nm on the surface of NF (Figure 2b).^77^ Due to the ultrasmall thickness of nanosheets, abundant porous structure, and excellent electrical conductivity, the freestanding NiFe–MOF electrode exhibited remarkable catalytic performance for electrochemical water splitting. Electrodeposition, as a different solution deposition method, is also a versatile technique to prepare freestanding MOF-based electrodes, which is usually carried out in a three/two-electrode electrochemical cell with selected conductive substrates as working electrodes.^71,78−81^

In comparison to other preparation strategies, the advantages of this method include a high synthesis rate and controllable mass loading. In particular, the mass loading of MOF-based materials can be controlled accurately by adjusting the electrodeposition time or current density/potential. Using such a universal electrodeposition method, as shown in Figure 2c, uniform zeolitic imidazolate framework (ZIF) films were directly grown on various substrates (e.g., conductive glass, NF, CF, and CC) and have a nanostructured architecture (1D nanorod array, 2D nanowall array, and 3D nanoframework), which provided a homogeneous coating on the surface of substrates or nanostructured architectures for subsequent handling.^81^

#### 3.1.2 Template-Assistance Growth

Since many MOFs cannot be grown on substrates by the direct growth method, template-assistance methods play essential roles in preparing some of the freestanding MOFs.^64,82−86^ This strategy mainly utilizes nanostructured metal oxides/hydroxides or metals, which are easy to nucleate and grow on the surface of conductive substrates, as templates and/or metal sources for the conversion preparation of MOFs, providing a new approach to design freestanding MOF-based electrodes. In this respect, Jiang et al. developed a general strategy to grow various MOF-hybrid arrays (e.g., Co–MOF-74, ZIF-67, Ni–MOF-74, and HKUST-1) on different substrates (e.g., NF, Cu mesh, Cu foil, and Fe mesh) by using metal oxide and nanostructured hydroxide arrays as the self-sacrificing templates.^82^Figure 2d shows a schematic illustration of the metal oxide@MOF hybrid arrays fabricated by the template-assistance strategy. The corresponding scanning electron microscopy (SEM) image shows that the obtained ZIF-67 was uniformly coated on the surface of CoO nanorod arrays on NF, and the 3D nanorod arrays were still preserved intact (Figure 2e). This strategy provided an excellent example for the rational construction of well-aligned 3D MOF nanocrystals on substrates, serving as freestanding electrodes for energy storage and conversion. Inspired by this work, Yao et al. developed a facial and mild method to synthesize microcube-like K_2_Zn_3_(Fe(CN)_6_)_2_·9H_2_O (KZHCF) on CC using metallic Zn nanosheets as the Zn source and reducing agent (Figure 2f and 2g), which was used as the freestanding cathode for aqueous sodium-ion batteries (SIBs).^85^ Their results demonstrated that the construction of freestanding electrodes could achieve splendid ion transport and high electron conductivity.

#### 3.1.3 Hybridization

**Figure 3:**
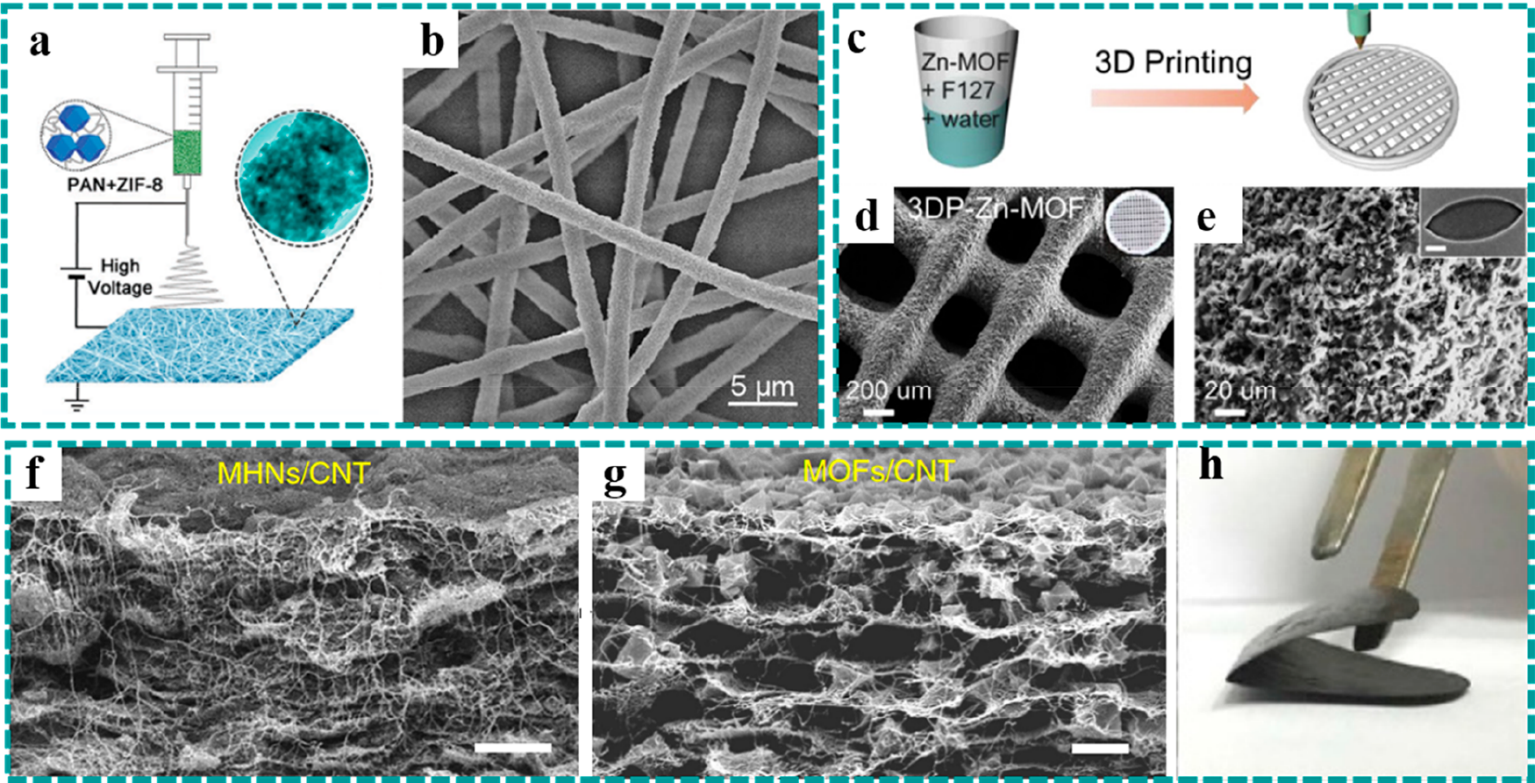
Synthesis strategies of freestanding MOFs. Hybridization: (a and b) freestanding ZIF-8 film prepared by electrospinning. Reproduced with permission from ref (87). Copyright 2017 Royal Society of Chemistry. (c–e) Three-dimensional Zn–MOF network achieved by 3D printing. Reproduced with permission from ref (101). Copyright 2020 Elsevier. (f–h) Freestanding H_3_BTC/CNT hybrid film constructed by filtration. Reproduced with permission from ref (96). Copyright 2017 The Author(s).

In addition to the above-listed strategies, hybridization is another common way to achieve the conversion from MOFs in powder form to freestanding MOFs by a specific technology, such as electrospinning, filtration, 3D printing, etc.^87−101^ Electrospinning, as one of the most effective methods to synthesize 1D nanostructured fibers with controllable diameters, has attracted tremendous attention in energy storage and conversion. For example, Lou et al. prepared a freestanding film composed of polyacrylonitrile (PAN) and ZIF-8 composite nanofibers by a facile electrospinning method (Figure 3a).^87^ Their SEM studies showed that ZIF-8 nanoparticles were uniformly embedded into electrospun 1D PAN fibers (Figure 3b), and they could convert to a freestanding film electrode consisting of a 1D carbon-containing composite after subsequent carbonization, accordingly forming a continuous conductive network and even carbon coating for better rate capability and cycle stability. Three-dimensional printing technology, possessing a similar fabrication mechanism as the former, has been applied to fabricate 3D-structured freestanding electrodes owing to the unique quality of the rapidly constructed complex 3D architectures. One of the advantages of 3D printing is the ability to use computer software to design complex electrode structures. For example, a 3D freestanding Zn–MOF precursor with mesh-like architecture was constructed by an extrusion-based 3D printing technology (Figure 3c), which consisted of uniform filaments with a diameter of ∼200 μm and abundant macropores (∼300 μm) as shown in Figure 3d and 3e.^101^ In addition, filtration is also a standard and simple technology to construct freestanding MOFs by mixing MOF materials and conductive nanostructure materials, like reduced graphene oxide (rGO) and carbon nanotubes. Nevertheless, the uniform distribution of active materials is a commonly known challenge for the filtration strategy, limiting its wide application on the construction of freestanding electrodes. Peng et al. put forward an ingenious method to construct a freestanding film with the uniform distribution of MOFs (Figure 3f–h).^96^ Benefiting from the electrostatic self-assembly, the positively charged copper hydroxide nanostrands (CHNs) and pretreated negatively charged CNTs were mixed homogeneously and then filtrated into a freestanding hybrid film (Figure 3f), which was further transformed into a freestanding MOF film after immersion in a 1,3,5-benzenetricarboxylic acid (H_3_BTC) solution. This strategy achieved the homogeneous distribution of MOFs and the tight coupling between MOFs and CNTs (Figure 3g), laying a good foundation for the subsequent acquisition of high-performance freestanding electrodes. It is worth noting that the freestanding MOFs obtained by the above two methods usually need to be further converted to freestanding electrodes for EESC.

#### 3.1.4 Other Preparation Strategies

**Figure 4:**
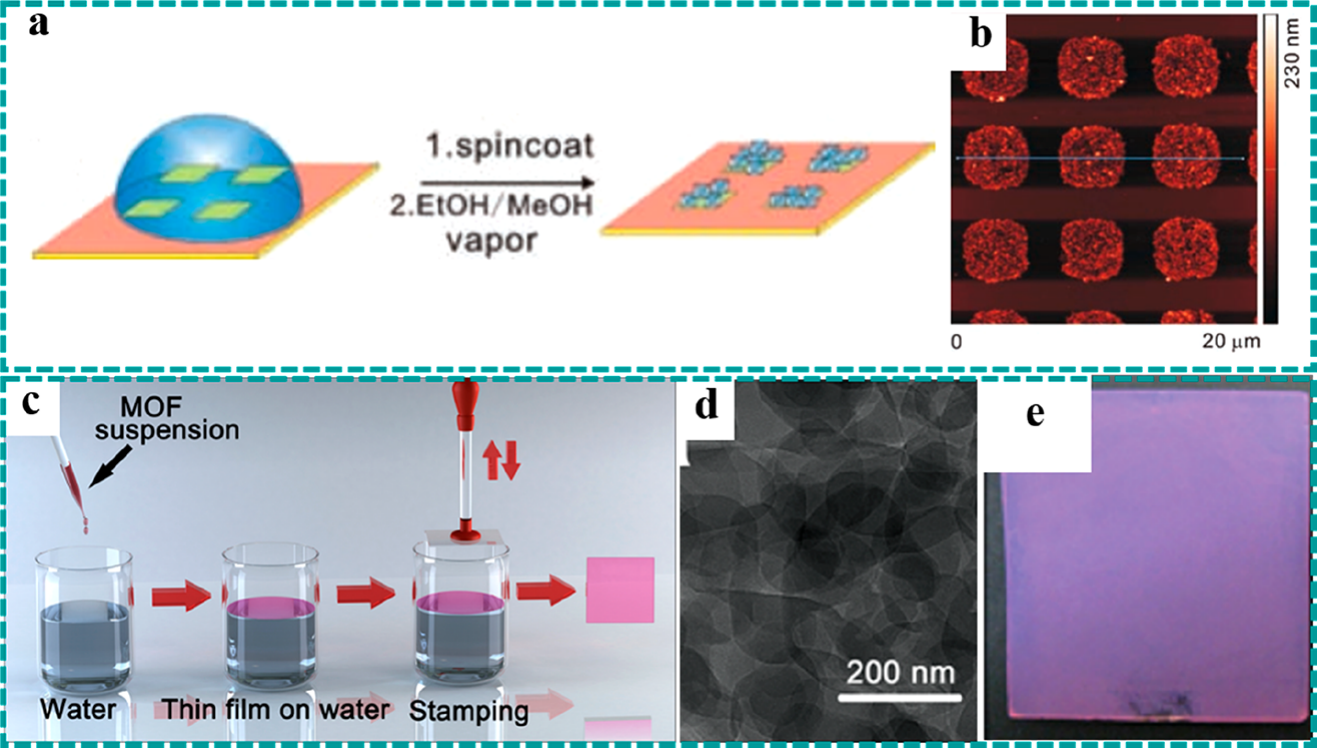
Synthesis strategies of freestanding MOFs: other preparation strategies. (a) Preparation schematic and (b) AFM image of HKUST-1 film patterns by combining spin coating with microcontact printing. Reproduced with permission from ref (107). Copyright 2011 Wiley-VCH. (c) Preparation flow of the “modular assembly” strategy. (d) TEM image of Cu–TCPP nanosheets. (e) Optical photograph of MOF film. Reproduced with permission from ref (103). Copyright 2012 American Chemical Society.

There are several other ingenious synthesis methods for constructing freestanding MOFs, which have not served as the electrodes for EESC, for example, including microcontact printing and Langmuir–Blodgett techniques.^102−107^ Benefiting from the molecular design and assembly, they would be expected to provide new vitality for the preparation of freestanding MOF-based electrodes. Thus, microcontact printing is a microprocessing technology that combines elastic stamps with self-assembled monolayer technology to print micrometer- or submicrometer-siized graphics on a substrate. The technology is not only conductive to prepare highly oriented MOF films with controllable thickness but also able to largely reduce the synthesis time in comparison to most of the other preparation strategies. For example, Andreas Terfort et al. achieved the patterning of surface HKUST-1 films by combining spin coating and microcontact printing in which the whole synthetic process only took several minutes.^107^ As shown in the preparation schematic (Figure 4a), the microcontact printing technology was used to fabricate the hydrophilic/hydrophobic patterns to realize the regionalized growth of MOFs, where the 11-mercaptoundecanoic (green) acid and 1-hexadecanethiol (red) on gold substrates (yellow) served as the hydrophilic and hydrophobic materials, respectively. Under the ethanol vapor treatment, the MOF particles were fully filled in every square (∼4 μm × 4 μm) after 10 cycles of spin coating and the thickness was about 90 nm (Figure 4b). The thickness of the MOF pattern could be effectively controlled by adjusting the cycles of spin coating. Due to the high purity, effective controllability, and fast preparation characteristic, the microcontact printing technology is expected to enable extended functional applications of MOFs, especially for the construction of microdevices.

Different from the microcontact printing technology, the Langmuir–Blodgett technique provides an effective pathway to synthesize highly crystalline “modules” and transfers them to the target substrate in the following “assembly” step, the so-called “modular assembly” strategy.^103^ Compared to the traditional preparation strategies of MOF-based thin films, this technology displays the following advantages: (i) fast synthesis and controllable thickness, (ii) universal adaptation to various substrates, and (iii) overcoming the limitation of preparing highly oriented crystalline freestanding MOF thin films by the traditional strategies. As shown in Figure 4c, the as-synthesized Cu–TCPP nanosheets (Figure 4d) were uniformly dispersed in ethanol by ultrasonication and subsequently added dropwise to the surface of water to form the stable thin film, owing to the hydrophobic characteristic of the Cu–TCPP nanosheets.^103^ Finally, the target substrates were immersed into the water surface and achieved the rapid transfer of a MOF thin film by stamping 15 cycles (Figure 4e). The resulting thin film attached to the substrate exhibited high purity and orientation, and its thickness could be adjusted by repeated stacking, which was conductive to explore the structural transformation and reaction mechanism of MOF-based materials for EESC.

### 3.2 Preparation of Freestanding MOF Derivatives

Freestanding MOF derivatives exhibit numerous remarkable physical and chemical properties, which have been extensively explored on EESC. Due to the abundant porous structures and the existence of both metals and organic linkers, pristine MOFs are naturally promising precursors for various nanostructured metal compounds, heteroatom-doped carbons, and their composites. On the basis of the reaction mechanism during the conversion process, the fabrication of freestanding MOF derivatives can be divided into two categories: thermal transformation and ion exchange.

#### 3.2.1 Thermal Transformation

**Figure 5:**
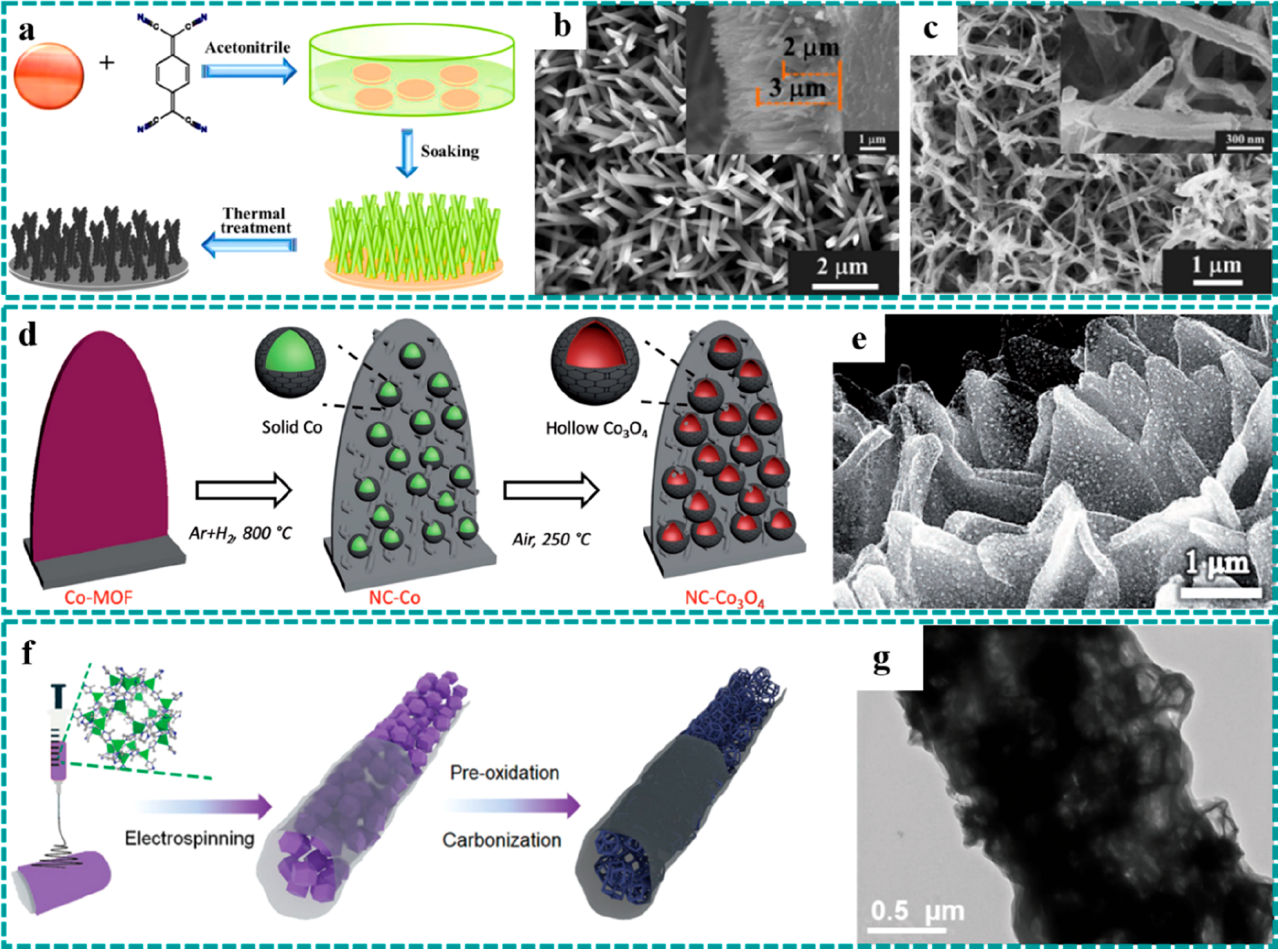
Synthesis strategies of freestanding MOF derivatives. Thermal transformation: (a–c) MOF-derived CuO nanorod arrays on CF derived by thermal annealing at 250 °C under an Ar and air atmosphere. Reproduced with permission from ref (67). Copyright 2017 American Chemical Society. (d and e) NC–Co_3_O_4_ nanoarrays synthesized by carbonization under an Ar/H_2_ atmosphere and subsequent thermal treatment in air. Reproduced with permission from ref (52). Copyright 2017 Wiley-VCH. (f and g) One-dimensional porous carbon nanofibers obtained by combination of electrospinning and carbonization. Reproduced with permission from ref (93). Copyright 2020 Royal Society of Chemistry.

Thermal transformation provides an effective pathway to prepare various freestanding MOF derivatives by direct pyrolysis of pristine MOFs at a specific temperature under a particular atmosphere (e.g., air, N_2_, or Ar). The difference of target derivatives usually means different pyrolysis temperatures and reactive atmospheres. To obtain MOF-derived nanostructured metal oxides or their composites, the thermal transformation reaction is generally carried out at a relatively low pyrolysis temperature (250–450 °C) under an air atmosphere,^65,66,108−110^ while a reaction at a high pyrolysis temperature (600–1000 °C) under a N_2_/Ar atmosphere is used to prepare MOF-derived nanostructured carbon or carbon-based composites.^63,76,87,97,111^ For example, as illustrated in Figure 5a, freestanding porous CuO nanorod arrays (Figure 5c) were derived from Cu–MOF grown on CF (Figures 5b) by simple thermal annealing at 250 °C in Ar and air atmospheres.^67^ The construction of freestanding MOF derivatives not only could overcome the poor contact problem between CF and CuO nanorod arrays but also avoided the addition of conductive carbon additives and binders, exhibiting outstanding electrochemical performance as the binder-free anode for lithium-ion batteries (LIBs). Besides, pristine MOFs have also been regarded as an ideal sacrifice template for porous heteroatom-doped carbons due to the large content of carbon-based organic linkers. In one of our previous works, hollow Co_3_O_4_ nanospheres embedded in nitrogen-doped carbon (NC) nanowall arrays derived from Co–MOF grown on flexible CC were prepared, serving as a freestanding electrode for Zn–air batteries.^52^ As shown in Figure 5d, during the annealing process at 800 °C under an Ar/H_2_ atmosphere, the organic linkers were converted into porous NC while the Co ions were reduced to Co nanoparticles, which were further transferred into hierarchical NC–Co_3_O_4_ nanoarrays after a subsequent thermal treatment in air. The SEM image (Figure 5e) of NC–Co_3_O_4_ showed the intact porous nanowall arrays on the surface of CC, which could significantly expose accessible active sites and promote the fast transport of ions and electrons. In another work, the combination of electrospinning and carbonization was used to construct freestanding MOFs derivatives.^93^ As illustrated in Figure 5f, nanostructured CoO_*x*_ derived from ZIF-67 and encapsulated into 1D porous carbon nanofibers was fabricated by electrospinning and a subsequent carbonization process, directly serving as freestanding electrodes for energy storage. Figure 5g shows that nanoframes of CoO_*x*_ were uniformly distributed on the carbon nanofibers with a diameter of ∼2 μm, which would benefit the fast transport of ions and electrons. Moreover, the carbon coating, derived from pristine MOFs and PAN, could effectively restrain the structure collapse resulting from the volume change during the charge/discharge processes.

#### 3.2.2 Ion Exchange

**Figure 6:**
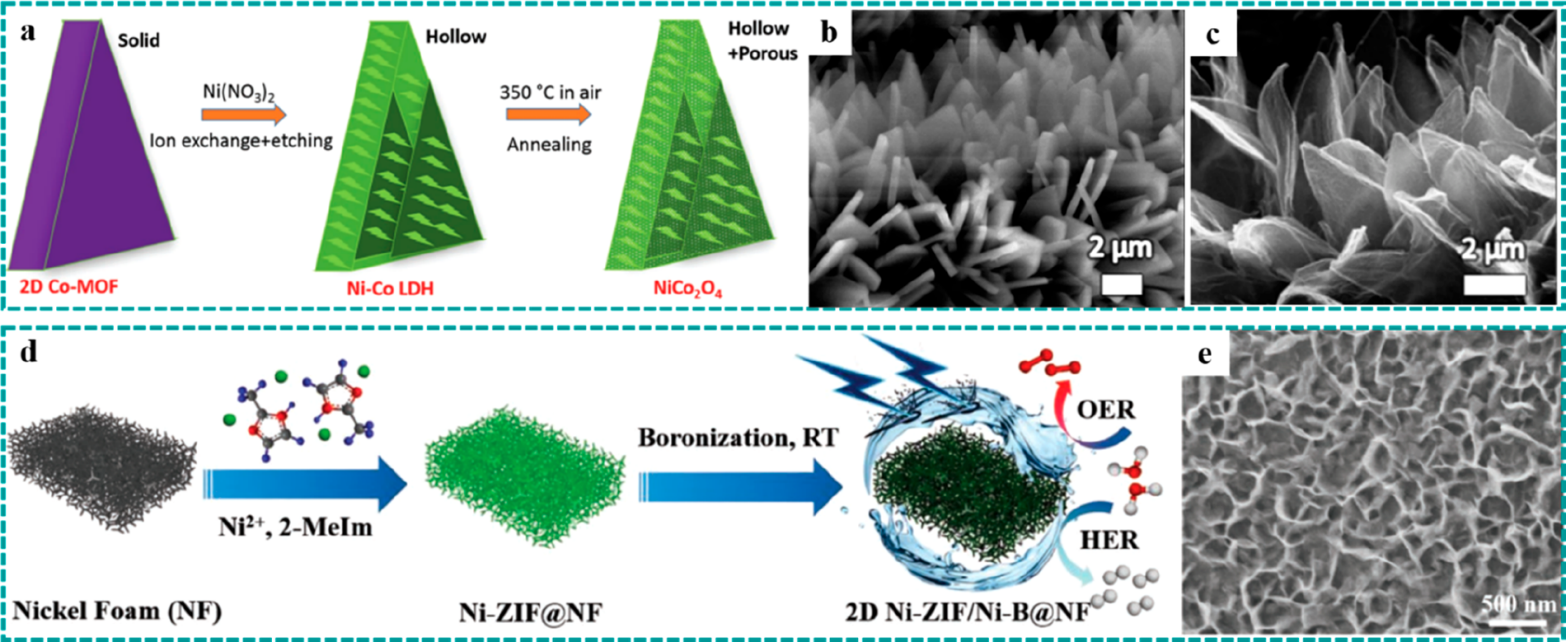
Synthesis strategies of freestanding MOF derivatives. Ion exchange: (a–c) hollow NiCo_2_O_4_ nanoarrays on CC derived by ion exchange and subsequent thermal treatment. Reproduced with permission from ref (49). Copyright 2017 Wiley-VCH. (d and e) Crystalline–amorphous Ni–ZIF/Ni–B nanosheets derived by a boronization strategy. Reproduced with permission from ref (116). Copyright 2020 Wiley-VCH.

In addition to the thermal transformation method, ion exchange, as a solid–liquid reaction, is also an effective pathway to prepare nanostructured MOF derivatives with tunable structures and compositions, which can occur at relatively mild conditions if the interaction force between the metal ion and organic linkers is weaker than that between the metal ion and other inorganic anions.^49,112−115^ Interestingly, the process can generate a unique transformation on the morphologies and structures. For example, in one of our previous works, we reported the preparation of a Ni–Co-layered double hydroxide (Ni–Co LDH) with a hollow wall structure derived from Co–MOF nanowall arrays grown on CC by a facial ion-exchange and etching process, which further transferred into crystallized NiCo_2_O_4_ after the subsequent thermal process (Figure 6a).^49^ As shown in the SEM images (Figure 6b and 6c), the process of ion exchange constructed the hollow structures with abundant porosity and a large specific surface area, which exhibited numerous advantages: accessible active sites, fast ion diffusion, and buffering the volume change. Besides metal oxides/hydroxides, other nanostructured metal compounds derived from pristine MOFs were also explored as freestanding electrodes for EESC. Recently, Wu et al. reported a simple room-temperature boronization strategy to synthesize ultrathin Ni–ZIF/Ni–B nanosheets derived from Ni–ZIF nanorods grown on NF, as illustrated in Figure 6d and 6e.^116^ Interestingly, partial crystalline Ni–ZIF would be preferentially transferred into amorphous Ni–B during the process, thus forming ultrathin crystalline–amorphous Ni–ZIF/Ni–B nanosheets, which could generate interesting qualities in the physical and chemical properties, such as phase boundaries for the improvement on catalytic performance.

## 4 Freestanding MOF-Based/Derived Electrodes for Energy Storage

**Figure 7:**
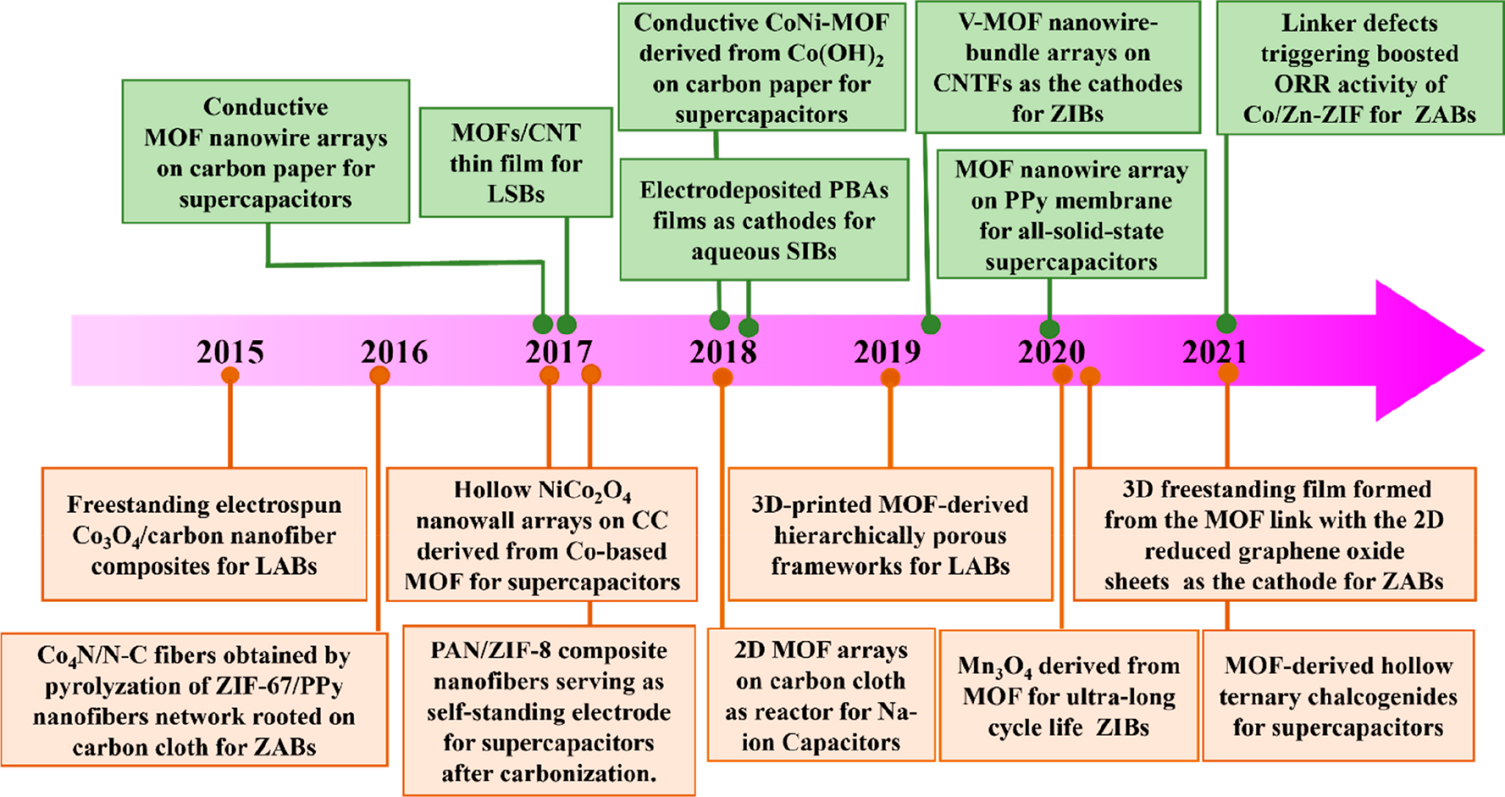
Key timeline of freestanding MOF-based/derived electrodes for various energy storage devices.

Energy storage, as the most important component in the development and utilization of clean energy, plays a critical role in our daily uses, such as smart mobile phones, electric vehicles, etc. Over the past two decades, intensive research on electrode materials has never stopped and great progress has been made. Among various electrode materials, freestanding MOF-based/derived electrodes have been demonstrated to exhibit remarkable potential owing to their unique advantages as highlighted in the previous part: large specific surface area, highly tunable porosities, abundant active sites, rapid electronic transmission, and seamless contact.^49,84,88,111,117^ The timeline in Figure 7 illustrates some of the important milestones of progress related to freestanding pristine MOFs (labeled as green) and their derivatives (labeled as orange) as electrodes for various energy storage devices. Due to the fact that most pristine MOFs possess poor electrical conductivity, freestanding MOF derivatives with enhanced conductivity were initially applied as electrodes for energy storage. As reported in a pioneer work published in 2015, combined with electrospinning and subsequent thermal treatment, freestanding ZIF-9-derived Co_3_O_4_/carbon nanofiber composites were used as binder-free electrodes for Li–air batteries (LABs).^118^ Next, ZIF-L, grown on different conductive substrates (e.g., CC, NF, CNTF, etc.), were used to derive various metal compounds or carbon-based composites, which have stimulated much interest in the field of energy storage.^49,87,88,100,109,111,114,119^ Recently, enormous efforts have been devoted to the design and preparation of freestanding MOF derivatives, preventing the damage and collapse of the MOFs’ original morphologies and porous structures under high-temperature conditions. Encouragingly, the emergence of conductive MOFs provides a great opportunity to directly develop into freestanding pristine MOFs as an electrode for energy storage. In 2017, conductive MOF nanowire arrays were first successfully grown on the surface of carbon paper, serving as freestanding electrodes for supercapacitors, which were shown to exhibit excellent electrochemical properties.^75^ Subsequently, a series of freestanding pristine MOFs were explored in various energy storage systems, such as supercapacitors, Li–S batteries (LSBs), Na-ion batteries (SIBs), Zn-ion batteries (ZIBs), and Zn–air batteries (ZABs), showing great application prospects in energy storage.^70,79,86,96,120,121^

### 4.1 Li-Based Batteries

In this section, Li-based batteries consisting of LIBs, LSBs, and LABs will be discussed separately. LIBs, commercialized by Sony Inc. in 1991, have been widely applied in electronic products owing to the satisfactory energy density, good cycling stability, and stable output voltage.^122−124^ However, the ever-increasing demand has promoted the search for next-generation energy storage devices with higher energy density. Compared to the conventional LIBs, LSBs and LABs have higher theoretical energy densities, exhibiting tremendous application potential in electric vehicles.^125,126^ Despite sharing different energy storage mechanisms by these three different types of batteries, freestanding MOF-based/derived electrodes with unique advantages have attracted wide attention for them. The relative works on freestanding MOFs and their derivatives as cathodes/anodes for Li-based batteries are summarized in Table S1.

#### 4.1.1 LIBs

LIBs have been the “workhorses” in portable electronics and more recently in electric vehicles due to their good overall electrochemical properties. Nevertheless, they still face numerous challenges, for example, capacity fading resulting from the large volume change in the ion intercalation/deintercalation processes and low power density because of the sluggish ion diffusion and transmission among reactive active sites as well as the poor safety.^127^ To address these issues, extensive efforts have been devoted to designing and preparing advanced nanostructured electrodes for desirable Li-storage properties.^62,128^ It is noteworthy that freestanding MOF-based/derived electrodes exhibit unique properties as advanced electrodes for LIBs.^67,95,99,115,117,129−136^ First, MOF-based/derived materials with large specific surfaces could provide more redox-active sites to accommodate Li^+^, leading to higher specific capacities and energy densities. Besides, the regular channels and porous structures of pristine MOFs and their derivatives can construct fast ways for the diffusion of Li^+^ and buffer the volume change during the lithiation/delithiation processes for stable cycle performance. Finally, the construction of freestanding electrodes can give a seamless contact between the conductive substrates and the MOF-based/derived materials, thus overcoming the limitations caused by inactive binders, which could facilitate charge transport to improve the overall conductivity of the electrode for outstanding power densities. As a result, the design of freestanding MOF-based/derived electrodes provides excellent opportunities owing to the diversification of conductive substrates, especially for those power sources of portable and wearable electronics.

**Figure 8:**
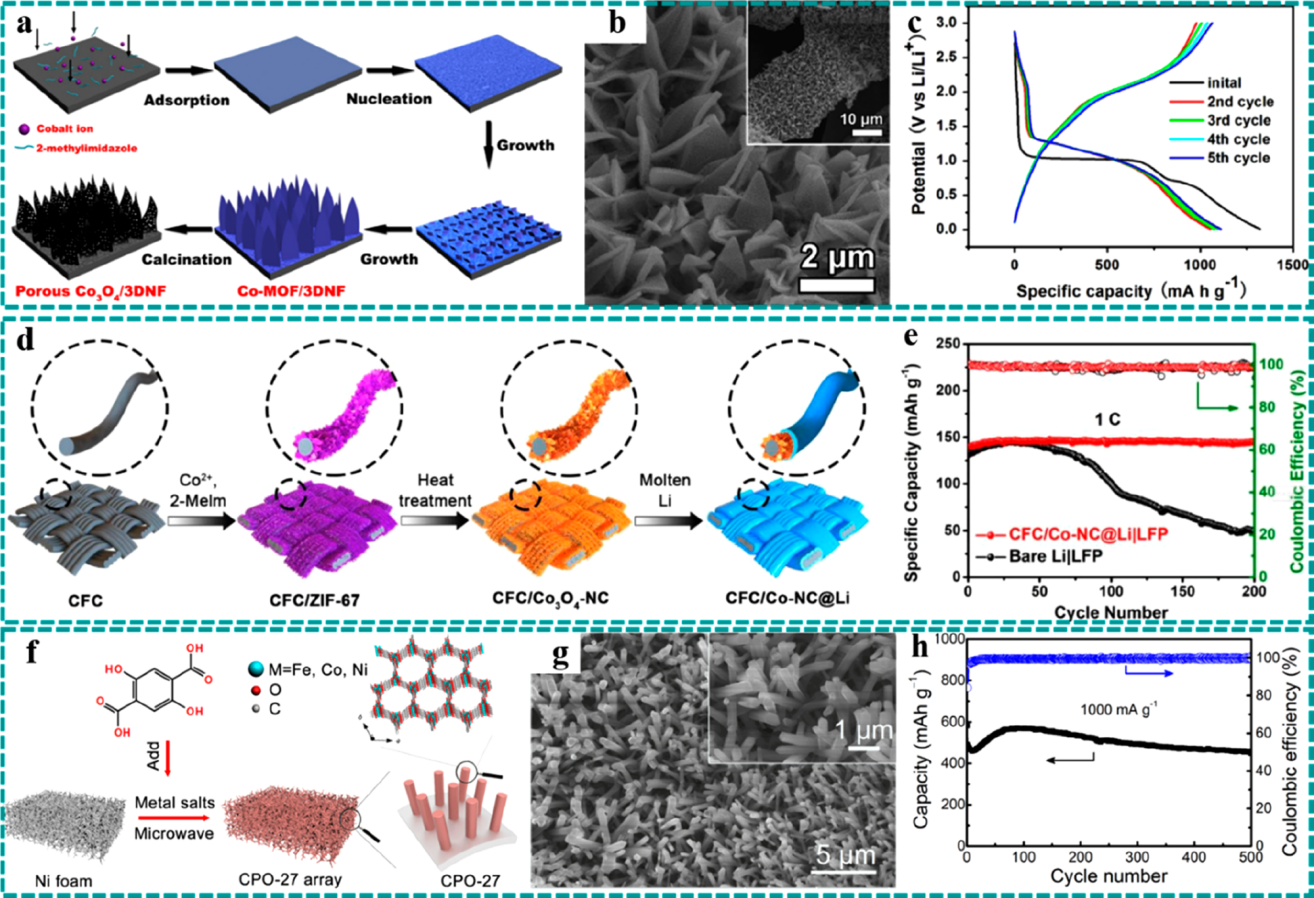
Freestanding MOF-based/derived electrodes for LIBs. (a) Schematic diagram of the preparation process, (b) SEM images, and (c) initial five charging/discharging curves of Co_3_O_4_/3DNF. Reproduced with permission from ref (132). Copyright 2016 Elsevier. (d) Preparation flowchart of CFC/Co_3_O_4_–NC@Li. (e) Cycle stability and Coulombic efficiency of CFC/Co_3_O_4_–NC@Li and control group. Reproduced with permission from ref (140). Copyright 2019 Elsevier. (f) Schematic diagram of the preparation strategy and structure, (g) SEM images, and (h) cycle stability and Coulombic efficiency at 1000 mA g^–1^ of the CPO-27 electrode. Reproduced with permission from ref (117). Copyright 2019 Elsevier.

In 2016, Liang et al. prepared porous Co_3_O_4_ nanosheets derived from Co–MOF grown on 3D NF and used them as freestanding anodes (Co_3_O_4_/3DNF) for LIBs.^132^ The detailed synthetic process of the Co_3_O_4_/3DNF electrode is illustrated in Figure 8a, in which porous Co_3_O_4_ nanosheets were obtained by simple calcination of Co–MOF at 300 °C in air. The SEM images (Figure 8b) show that Co_3_O_4_ nanosheets with porous structures were uniformly covered on the surface of NF. Highly porous structures were demonstrated to provide more abundant redox-active sites and shorten the Li^+^ diffusion distance. As shown in Figure 8c, the charging/discharging curves of the initial five cycles at 0.5 A g^–1^ almost coincided (except the first cycle), indicating a very stable electrochemical performance. Moreover, after 300 cycles at 0.5 A g^–1^, the freestanding electrode still maintained a high specific capacity of 1204 mAh g^–1^, further demonstrating the stable electrochemical performance of freestanding MOF-derived Co_3_O_4_ electrodes as advanced anodes for LIBs.

Except for the transition metal oxides, metallic lithium has been widely studied as anodes for LIBs because of their high theoretical capacity (3860 mAh g^–1^), low density (0.59 g cm^–3^), and very low negative electrochemical potential (−3.04 V vs standard hydrogen electrode). However, the growth of Li dendrite and the change of electrode volume during long-term charging/discharging processes severely limit the further development of metallic Li anodes. Composite metallic Li anodes with freestanding 3D carbon-based networks have thus been proven to be an effective strategy for high-performance metallic Li anodes.^137−139^ For example, Li et al. reported Co_3_O_4_-embedded and nitrogen-doped porous carbon nanoflake arrays (Co_3_O_4_–NC) derived from Co–MOF grown on carbon fiber cloth (CFC) as a 3D stable host to prestore molten Li for high-performance metallic Li anodes, as illustrated in Figure 8d.^140^ The components of the freestanding MOF derivatives had synergistic effects on the electrochemical performance. First, the reaction between Co_3_O_4_–NC and molten Li significantly contributed to the increment of lithiophilicity of the CFC/Co_3_O_4_–NC host. Second, NC nanosheets, directly grown on conductive substrates, exhibited excellent chemical stability and high electrical conductivity, which could help keep the structural stability of the CFC/Co_3_O_4_–NC host during the coverage process of molten Li and achieve the rapid transmission of the electron and ion during the charging/discharging processes, respectively. Besides, the freestanding MOF derivatives possessed a large specific surface area and highly porous structures, which are conducive to hinder the growth of Li dendrite and buffer the volume change during long-term charging/discharging processes. As expected, the assembled cell, using CFC/Co_3_O_4_–NC@Li as the anode, could maintain ∼100% of the initial capacity (Figure 8e), greatly exceeding the cell’s performance using bare Li as the anode.

High-temperature thermal treatment is a common practice to convert pristine MOFs into metal oxides or carbon-based materials with improved conductivity as the electrode materials for LIBs. However, the high temperature employed could destroy intrinsic channel structures and active sites of pristine MOFs. Therefore, developing advanced freestanding pristine MOFs directly as the electrodes for LIBs would be needed. Recently, Li et al. prepared 3D multicomponent CPO-27 nanorod arrays on the surface of NF by a facile microwave-assisted solvothermal method, as illustrated in Figure 8f and 8g, directly used as the anode for LIBs.^117^ In this work, two approaches were adopted to overcome the limitation of low conductivity: on one hand, the interaction between multiple metal components (Fe, Co, and Ni) effectively facilitated electronic delocalization of pristine MOFs and thus improved the electrical conductivity of the electrode. The 3D freestanding nanorod arrays, on the other hand, considerably shortened the transmission and diffusion distance of the electrons and ions. As a result, the freestanding CPO-27 electrode achieved a high mass capacity of 834 mAh g^–1^ at 50 mA g^–1^ and maintained 52.8% of the initial capacity as the current density increased to 2000 mA g^–1^, exhibiting an outstanding rate capability. Moreover, the freestanding electrodes showed excellent electrochemical stability (Figure 8h), retaining 93% of the second-cycle capacity after 500 cycles at 1000 mA g^–1^.

#### 4.1.2 LSBs

Due to the extremely high theoretical energy density (2600 Wh kg^–1^) and abundant low-cost sulfur resources, LSBs, one of the most promising candidates for next-generation energy storage devices, have been widely studied with great progress made in recent years.^141^ Nevertheless, it is still challenging for LSBs to be commercialized like LIBs because of the following limitations: (i) the intrinsic insulation nature of a sulfur cathode leads to sluggish electrochemical kinetics and severe polarization,^142^ (ii) the solubility and shuttle of intermediate polysulfides (LiPSs) in organic electrolytes result in poor cycle stability,^143^ (iii) the large volume change of cathodes during the charging/discharging processes further causes the fast capacity fading,^144^ and (iv) the damaging issues caused by shuttling of polysulfides to lithium anode.^145^ To overcome the above obstacles, a feasible approach is to construct nanostructured host materials with high conductivity and abundant porosity to enhance the overall conductivity of the target cathode and hinder the shuttle effect of soluble LiPSs.^146^ In this connection, freestanding MOF-based/derived electrodes possess present favorable potentials.

**Figure 9:**
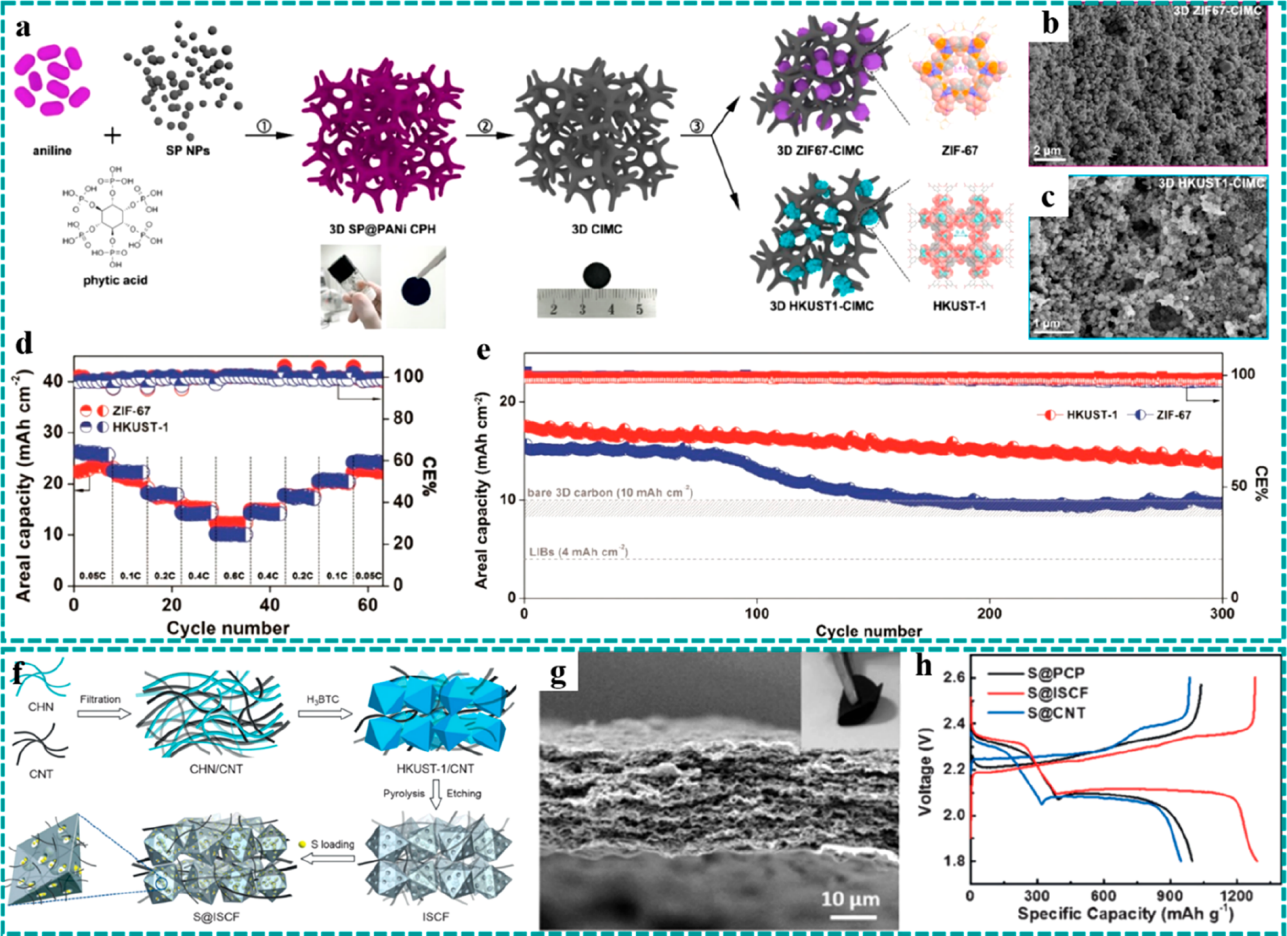
Freestanding MOF-based/derived materials served as host electrodes for LSBs. (a) Schematic of the fabrication processes, (b and c) SEM images, (d) rate performance with the corresponding Coulombic efficiencies, and (e) cycle stabilities with the corresponding Coulombic efficiencies at 0.2 C of ZIF-67-CIMC and HKUST1-CIMC. Reproduced with permission from ref (53). Copyright 2019 American Chemical Society. (f) Schematic diagram of the preparation process. (g) SEM and optic images of S@ISCF. (h) Charging/discharging curves of S@ISCF and control groups. Reproduced with permission from ref (92). Copyright 2017 Wiley-VCH.

Tricoli et al. developed a conducting polymer hydrogel-integrated freestanding 3D monolithic covered by uniform MOF nanoparticles (ZIF-67 and HKUST-1) as the electrode architecture for high-performance LSBs, as shown in Figure 9a–c.^53^ The hierarchical monolithic 3D carbon networks (CIMC) facilitated the transport of electrons and diffusion of ions and enhanced the mechanical and chemical stability of the cathode. More importantly, the existence of MOF nanoparticles greatly improved the sulfur loading and limited the shuttle effect of soluble LiPSs, thus achieving high areal/volumetric capacity and excellent cycle stability. Taking HKUST-1 as an example, the resulting electrode delivered a high specific capacity of 1377 mAh g^–1^ at 0.05 C and still kept a capacity of 541 mAh g^–1^ as the current density was increased to 0.60 C (Figure 9d), exhibiting an excellent rate capability. In addition, it is worth mentioning that the HKUST1-CIMC electrode delivered more outstanding cycle stability (0.06% decay per cycle) compared with the ZIF-67-CIMC electrode (0.13% decay per cycle) (Figure 9e). The phenomenon is mainly attributed to the larger pore size (0.9 nm) of HKUST1 than that of ZIF-67 (0.34 nm), which can allow the access of elemental sulfur (0.68 nm) and LiPSs (>0.4 nm) into its porous structure and thus effectively hinders the shuttle effect of soluble LiPSs, achieving better electrochemical stability.

In addition, freestanding porous carbon-based materials derived from pristine MOFs have been regarded as promising sulfur hosts for LSBs owing to their excellent electrical conductivity and high porosity, which can promote sulfur electrochemical activity and offer effective sulfur confinement.^92,97,98,147^ For example, Peng et al. developed an ingenious strategy to prepare an interpenetrated and self-standing conductive framework (ISCF) by combining CNTs with highly porous MOF-derived carbon polyhedrons.^92^ As illustrated in Figure 9f, a flexible freestanding thin film was initially obtained by simple filtration, where the filter liquor consisted of positively charged copper hydroxide nanostrands (CHN) and negatively charged CNTs. Then, the CHN/CNT thin film with a thickness of 4.3 μm was converted into the HKUST-1/CNT film by a template-assistance growth method. Finally, the freestanding sulfur host ISCF was developed by subsequent pyrolysis and etching, which directly served as the sulfur cathode for LSBs after loading sulfur. SEM and optical images (Figure 9g) showed the flexible freestanding ISCF film with a thickness of ∼20 μm in which the MOF-derived carbon polyhedrons uniformly dispersed in the laminated structure of the ISCF film. Benefiting from highly porous carbon polyhedrons, abundant inner space, continuous conductive networks, and freestanding electrode design, the electrode delivered a high initial capacity of 1290.9 mAh g^–1^ at C/5 (Figure 9h) and excellent cycle stability with a negligible capacity fading rate of 0.0054% per cycle at 1 C.

#### 4.1.3 LABs

LABs, consisting of an oxygen cathode and a metal Li anode, show great potential as another group of promising alternatives to the traditional LIBs because of their ultrahigh theoretical energy density of 11 140 Wh kg^–1^.^148,149^ Nevertheless, the application of LABs still faces enormous challenges, for example, (i) the sluggish reaction kinetics of the oxygen cathode (oxygen reduction reaction (ORR) and oxygen evolution reaction (OER)) leads to a large overpotential and inferior rate performance, (ii) some of the extra products (e.g., Li_2_CO_3_) generated by the inevitable side reactions could block the active sites of the oxygen catalysts and hinder the diffusion of electrolyte ions, and (iii) the sluggish ion diffusion and electron transport would lead to low power densities.^128,150^ To overcome these challenges, developing electrode materials with reasonable structures would be the key for high-performance LABs with low overpotentials and excellent cycle stabilities.

**Figure 10:**
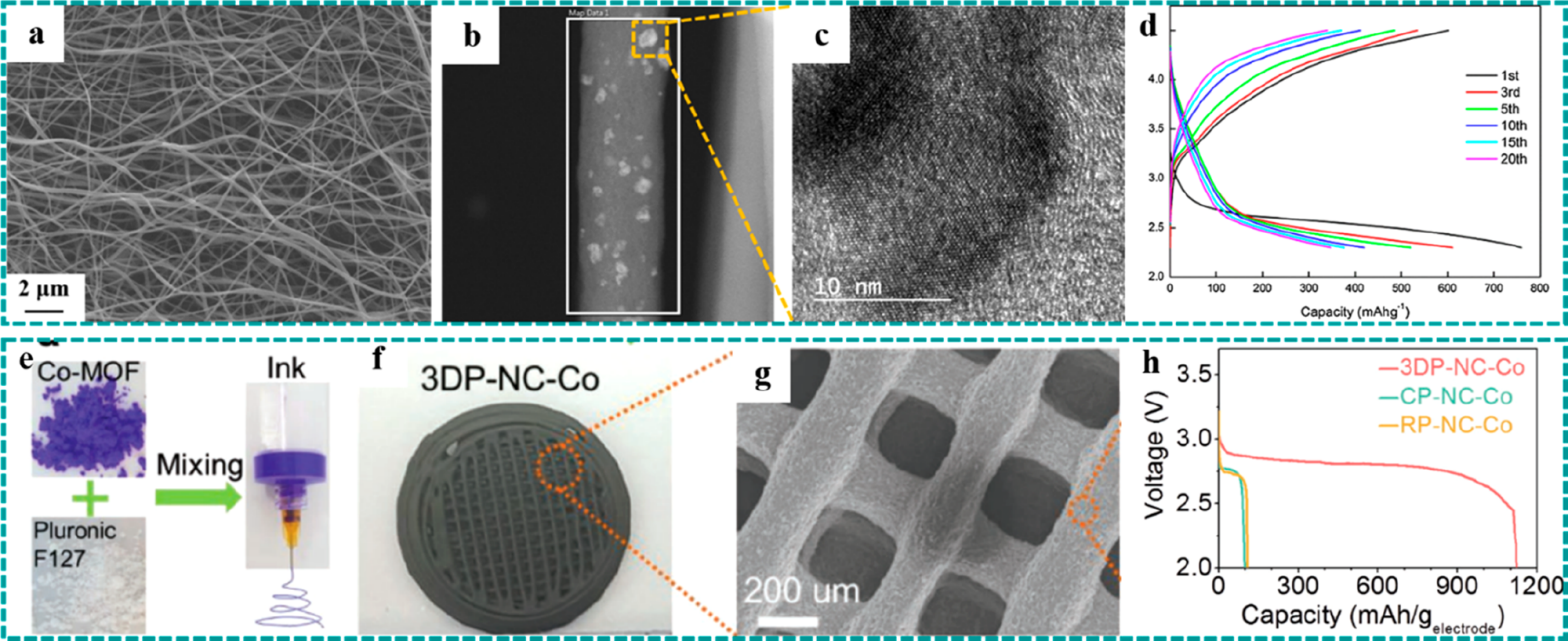
Freestanding MOF derivatives as electrodes for LABs. (a) SEM image, (b and c) TEM images, and (d) charging/discharging curves at 0.5 A g^–1^ of the Co_3_O_4_ cathode. Reproduced with permission from ref (118). Copyright 2015 Elsevier. (e and f) Optical image and (g) SEM image of the 3DP–NC–Co electrode. (h) Discharging curves of 3DP–NC–Co and control groups at 0.05 mA cm^–2^. Reproduced with permission from ref (100). Copyright 2019 Wiley-VCH.

Freestanding MOF derivatives have been studied as a class of competitive candidates as electrodes in LABs due to their large surface area, high porosity, accessible metal sites, and high electron/ion conductivity, which could provide abundant sites for adsorption and transmission of O_2_.^100,110,118^ For example, Shin et al. reported freestanding Co_3_O_4_/carbon nanofiber composites derived from ZIF-9 by electrospinning and subsequent thermal treatment, directly serving as the binder-free cathode for LAB.^118^ The 3D interlaced network structure could be maintained after carbonization and oxidation in Figure 10a, exhibiting good structural stability. Besides, the high-resolution TEM images (Figure 10b and 10c) show the uniform distribution of Co_3_O_4_ nanoparticles. Benefiting from the new design of the freestanding electrode, the uniform distribution of Co_3_O_4_ nanoparticles, and the continuous conductive network, the composite electrode delivered an initial discharge capacity of 760 mAh g^–1^ (Figure 10d) and stable cycle stability as well as a low charge overpotential.

In addition to improving the electrochemical performance, constructing freestanding 3D hierarchical-structure MOF derivative electrodes with flexible conductive substrates (e.g., CC, CNTFs, carbon papers, etc.) is an effective pathway to develop high-performance wearable energy storage devices. For example, 3D porous Co_3_O_4_ nanosheets derived from Co–MOF were grown on carbon textiles via a simple chemical bath deposition and subsequent annealing treatment, directly serving as the freestanding cathode for LABs.^110^ After annealing treatment, the as-obtained Co_3_O_4_ could maintain the intact nanosheet arrays, indicating the outstanding structural stability. Due to the decomposition of the organic composition, the MOF-derived Co_3_O_4_ nanosheets exhibited a large specific surface area and abundant porous structure, which promoted the rapid diffusion and transport of electrolyte ions and offered more accessible sites for the accommodation of Li_2_O_2_. Combined with the design of the freestanding electrode, the flexible cathode delivered a high capacity of 6509 mAh g^–1^ at 200 mA g^–1^. Interestingly, compared to the freestanding MOF-derived Co_3_O_4_ electrode, the powder-form MOF-derived Co_3_O_4_ electrode only achieved a relatively lower capacity of 5018 mAh g^–1^, further demonstrating the unique advantages of freestanding architectures. Moreover, the as-fabricated flexible LAB using Co_3_O_4_/carbon textile as the freestanding cathode exhibited excellent mechanical flexibility, which proved their application potential in energy storage devices for wearable electronics.

Recently, 3D printing technology has shown great application potential in preparing new materials and especially unique structures where one can construct complex 3D architectures to satisfy the requirements for different applications.^6,151^ In one of our previous works, we reported a 3D freestanding highly porous catalyst of Co nanoparticles assembled in nitrogen-doped mesoporous carbon flakes (3DP-DC-Co) derived from Co–MOF using 3D printing technology and subsequent carbonization.^100^Figure 10h shows the preparation process of the 3D freestanding Co–MOF framework through an extrusion-based 3D printer, where printable ink was made to consist of presynthesized Co–MOF and Pluronic F127 powder. After carbonization treatment in the N_2_ atmosphere, the 3D porous framework without cracking was well maintained (Figure 10i and 10j), exhibiting excellent mechanical stability. Benefiting from the 3D porous framework structure, the existence of Co-based electrocatalysts, continuous conductive networks, and design of the freestanding electrode without substrates, the as-obtained cathode achieved a high specific capacity of 1124 mAh g^–1^ at a current density of 0.05 mA cm^–2^ (Figure 10k). Compared to the 3DP–DC–Co electrode, the CP–NC–Co and RP–NC–Co electrodes with substrates (carbon papers) delivered a much lower specific capacity, mainly attributed to the high mass proportion (∼75%) of the current collectors.

### 4.2 SIBs

Although LIBs possess excellent electrochemical properties and are occupying a dominant position in commercial energy storage markets, the grid-scale application of LIBs is still subject to the limited lithium source and the high cost in the long term as well as poor safety.^152,153^ Due to the abundant sodium resource and low cost as well as similar physical and chemical characters compared to Li, SIBs have emerged as a promising alternative to LIBs for large-scale energy storage. However, compared to Li^+^, Na^+^ possesses a much larger ionic radius and thus results in sluggish kinetics and a huge volume change during the charging/discharging processes, which lead to the inferior rate capabilities and cycle stabilities of SIBs.^154^ Therefore, most of the electrode materials constructed for LIBs are no longer applicable to SIBs, presenting the main bottleneck to restrict the development of SIBs. Freestanding MOF-based/derived electrodes with adjustable porosity and outstanding structural stability would be a good choice to realize the storage and release of Na^+^, which has been shown to be beneficial to construct high-performance SIBs with excellent power densities and cycle stabilities,^71,78,79,84,85,94,111,155^ as shown in Table S2.

**Figure 11:**
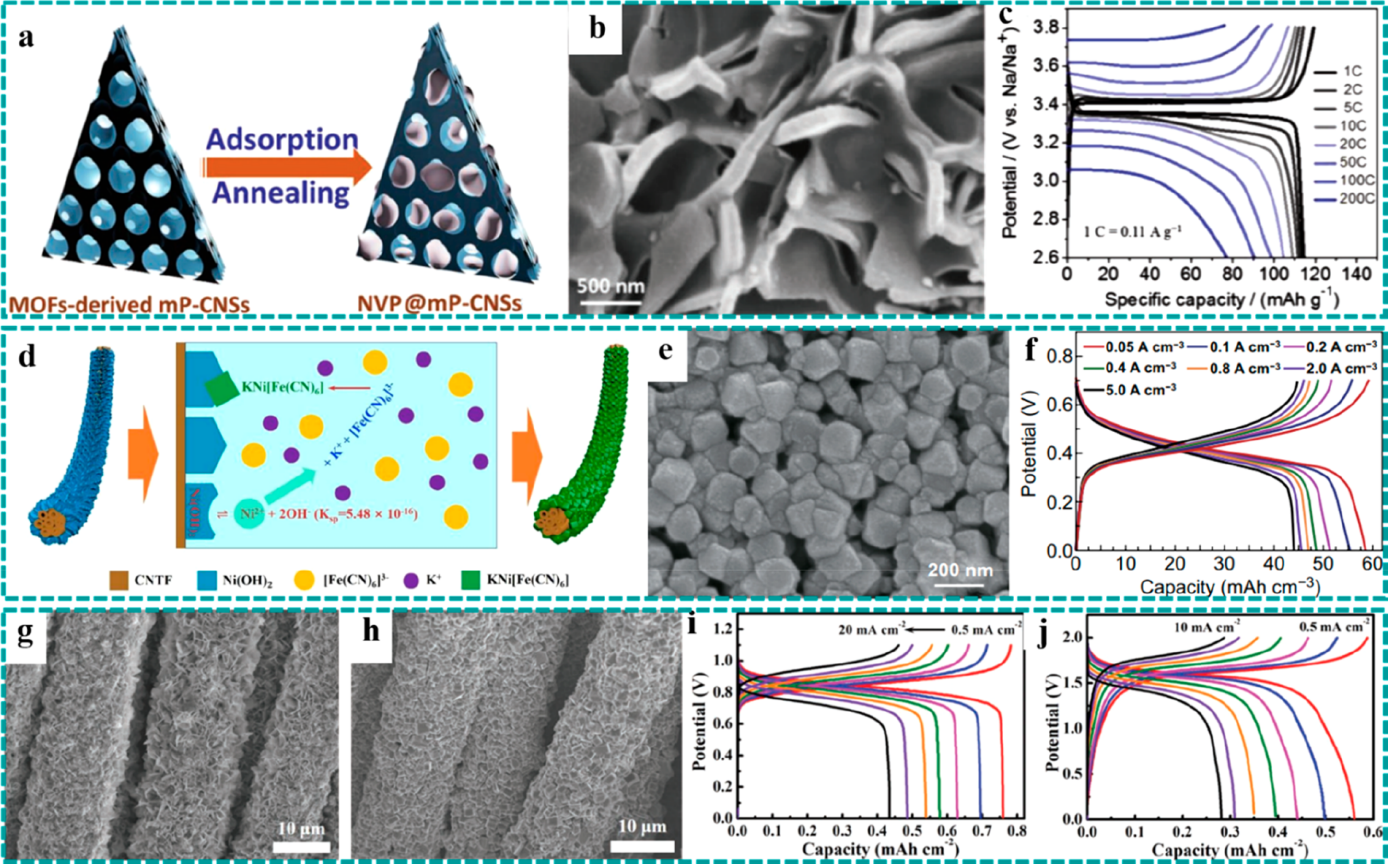
Freestanding MOF-based/derived materials used as electrodes for SIBs. (a) Schematic illustration of the preparation process of NVP@mP-CNSs by the adsorption of NVP and subsequent annealing. (b) SEM image and (c) charging/discharging curves at various current densities of NVP@mP-CNSs. Reproduced with permission from ref (111). Copyright 2018 Wiley-VCH. (d) Schematic diagram of the synthesis mechanism, (e) SEM image, and (f) charging/discharging curves at various current densities of KNHCF@CNTF. Reproduced with permission from ref (84). Copyright 2019 The Author(s). SEM images of (g) CC@Zn and (h) CC@KZHCF. Charge-discharge curves of (i) CC@KZHCF and (j) the assembled QSS SIB at various current densities. Reproduced with permission from ref (85). Copyright 2019 Wiley-VCH.

For SIBs, freestanding MOF-based/derived electrodes face both opportunities and enormous challenges. Thus, their synthesis processes have attracted extensive efforts. For example, Fan et al. developed an innovative method to utilize the oxygen/nitrogen functional groups in mesoporous carbon nanosheets (mp-CNSs) to immobilize metal ions through complexation or electrostatic interaction (Figure 11a), thereby preparing the freestanding anode for SIBs in which the mp-CNSs were derived from Co/Zn MOF arrays grown on CFC.^111^ As shown by the SEM image (Figure 11b), the original nanosheet arrays and the freestanding structure were well retained after adsorption and annealing treatment. The architecture exhibited unique advantages in electrochemical energy storage, including the following: (i) the uniform distribution and strong binding force would prevent the active materials from aggregating, (ii) the mesoporous carbon scaffold endowed the electrode material with high conductivity and accommodation for the volume change during the charging/discharging processes, and (iii) the design of a freestanding electrode based on flexible substrates provided an excellent opportunity for wearable energy storage devices. On the basis of these unique advantages, the anode delivered an excellent mass capacity of 117 mAh g^–1^ at 1 C and could maintain 70.1% of its initial capacity as the current density increased to 200 C (Figure 11c), demonstrating ultrafast charging/discharging characteristics. In addition, Prussian blue and its analogues (PBAs, A_*x*_M_1_[M_2_(CN)_6_]·*w*H_2_O) possess open framework structures and large interstitial sites, which are constructed by the N and C atoms from the inorganic cyanide bridges and combine with the M_1_ and M_2_ atoms, respectively. Due to the similar structural features with those of typical MOF materials, PBAs have been widely regarded as a member of the MOF family,^14,38^ and they have been demonstrated as promising cathode materials for metal-ion batteries, including SIBs, where they help facilitate the wanted fast intercalation and deintercalation of metal ions with large radii, such as Na^+^. Combined with the high electronic conductivity of freestanding structures, the freestanding PBAs electrodes exhibit excellent overall conductivity and thereby deliver remarkable rate capability and power density. Nevertheless, the coprecipitation method is a primary method to synthesize PBA active materials, where it is difficult to form strong adhesion between the active materials and the conductive substrates. To address this problem, Yao et al. used the Ni(OH)_2_ nanosheets as the sacrificial template to prepare KNiFe(CN)_6_ (KNHCF) nanocubes on the surface of CNTFs by chemical etching (KNHCF@CNTF), as illustrated in Figure 11d.^84^Figure 11e shows the SEM image of the freestanding KNHCF@CNTF cathode, implying that the KNHCF nanocubes were uniformly covered on the surface of CNTF. An aqueous electrolyte with a high ionic conductivity of ∼1 S cm^–1^ (organic electrolytes ≈ 1–10 mS cm^–1^) was adopted for high-power density aqueous SIBs. As a result, KNHCF@CNTF delivered a volumetric capacity of 58.54 mAh cm^–3^ at a current density of 0.05 A cm^–3^ and remarkable rate capability (72.7% retention as the current density increased to 5.0 A cm^–3^) (Figure 11f). The superfast charging/discharging characteristic was attributed to the following aspects: (i) the open framework of KNHCF for fast ion diffusion, (ii) the freestanding electrode enabling rapid electron transport, and (iii) the high ionic conductivity of the aqueous electrolyte. Combined with NaTi_2_(PO_4_)_3_ on CNTF as the anode, a quasi-solid-state (QSS) fiber-shaped aqueous SIB was successfully assembled and delivered an outstanding volumetric power density and mechanical flexibility. However, the low discharging voltage (∼0.4 V) of KNHCF@CNTF could lead to a relatively low energy density. Subsequently, Yao et al. further developed microcube-like KZHCF on CC (CC@KZHCF) by a mild water bath method (Figure 11h) in which metallic Zn nanosheets grown on CC served as the zinc sources and reductant (Figure 11g).^85^ The obtained CC@KZHCF was used as a direct freestanding cathode for aqueous SIBs, which exhibited a high output voltage of ∼0.8 V and delivered an areal capacity of 0.76 mAh cm^–2^ (0.5 mA cm^–2^) (Figure 11i). Likewise, a QSS aqueous SIB using freestanding KZHCF and NaTi_2_(PO_4_)_3_ as the cathode and anode, respectively, achieved a stable output voltage of ∼1.6 V and energy density of 0.92 mWh cm^–2^ (Figure 11j).

### 4.3 Zn-Based Batteries

Zn-based batteries have shown great potential as a class of the next-generation energy storage devices for portable and wearable electronics because of the abundant Zn source, low cost, high safety, and excellent aqueous electrolyte compatibility.^156,157^ Moreover, Zn^2+^/Zn couples involve a two-electron redox, which can offer a high theoretical capacity and energy density. Zn-based batteries include ZABs,^158,159^ Zn–Ni/Co batteries (ZN/CBs),^160,161^ and ZIBs^162,163^ in which the first two use alkaline electrolytes and the last one uses neutral/weak acid electrolytes. Although metallic Zn as the anode provides a high specific capacity and stable output voltage, the general lack of high-performance cathode materials to a certain extent limits the further development of Zn-based batteries. As mentioned in the section on Li-based batteries, freestanding MOF-based/derived electrodes could provide a large surface area, porous structures, abundant metals/metal oxides-based or carbon-based active sites, and high electrical conductivity, thereby attracting rather extensive research interest for aqueous Zn-based batteries. The electrochemical performance of freestanding MOF-based/derived electrodes for Zn-based batteries reported in recent years is displayed in Tables S3 and S4.

#### 4.3.1 ZABs

ZABs, using a silver wire as the cathode, were initially reported in 1878.^157^ Then, the primary ZABs were commercialized in the 1930s and applied in several types of applications.^157^ However, the primary batteries cannot meet the requirement of rechargeable power supplies for electronic devices nowadays. In the past few years, the rapid development of material science has also promoted continuous breakthroughs in the development of rechargeable ZABs.^164^ Nonetheless, they are still in the primary stage of development and encounter a series of challenges, including high polarization and low power density caused by the sluggish kinetics and limited reactant diffusion.^164,165^ In the rechargeable ZAB system, the air cathodes, in which the ORR and OER occur during the discharging and charging processes, play an important part in the electrochemical properties of the cells. Therefore, constructing high-performance cathodes with reasonable structures is key to overcoming the above challenges.

**Figure 12:**
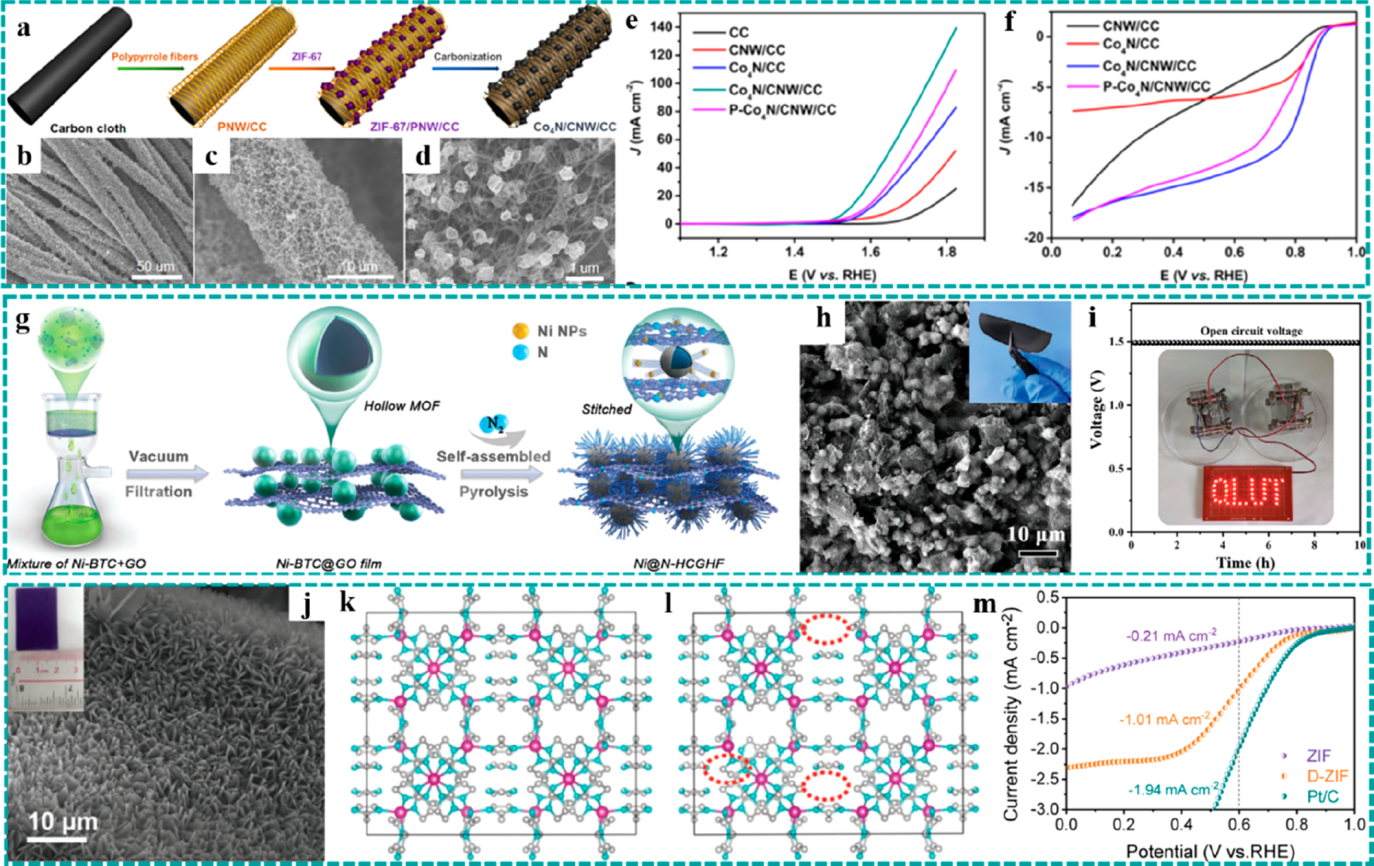
Freestanding MOF-based/derived electrodes for ZABs. (a) Schematic illustration of a synthetic strategy and (b–d) SEM images under different resolutions of Co_4_N/CNW/CC. LSV curves of Co_4_N/CNW/CC and control groups for (e) the OER and (f) the ORR. Reproduced with permission from ref (119). Copyright 2016 American Chemical Society. (g) Formation process and (h) SEM and optic images of Ni@N-HCGHF. (i) Open-circuit plots for 10 h of the assembled ZAB using Ni@N-HCGHF as a cathode (inset: optical image of lighted LEDs powered by two ZABs in series). Reproduced with permission from ref (88). Copyright 2020 Wiley-VCH. (j) SEM and optical images of D-ZIF. Structural schematics of (k) ZIF and (l) D-ZIF. (m) LSV curves of D-ZIF and control groups for the ORR. Reproduced with permission from ref (121). Copyright 2021 Wiley-VCH.

In this respect, freestanding MOF derivatives provide unique advantages: (i) MOF-derived nanometer-size active species (nitrides, carbides, oxides, and metal atoms) possess high catalytic activities for the OER and ORR, (ii) porous structures formed by carbonization promote the rapid diffusion of ions and reactants, and (iii) the carbon-based framework after carbonization and the design being freestanding form a continuous conductive network and expose abundant accessible active sites.^52,88,119,121,166−173^ For example, Zhang et al. developed an innovative strategy to prepare a 3D freestanding bifunctional catalyst on flexible CC, combining Co_4_N nanoparticles with continuous carbon fibers networks (CNW), as the binder-free cathode (Co_4_N/CNW/CC) for ZABs in which Co_4_N and CNW were derived from ZIF-67 and PPy nanofibers, as illustrated in Figure 12a.^119^ Moreover, they made full use of the excellent OER and ORR properties of Co_4_N and Co–N–C, respectively, overcoming the catalytic performance limitation of a single material. The SEM images (Figures 12b–d) showed that the 3D CNW inlaid with Co_4_N nanoparticles was uniformly covered on the surface of the CC fibers, providing a large surface area and fast electron transport for the ORR and OER. Benefiting from this ingenious design, the Co_4_N/CNW/CC was shown to exhibit outstanding OER performance with a low overpotential (at 10 mA cm^–2^) of 310 mV, and it also showed remarkable ORR performance with a high half-wave potential (*E*_1/2_) of 0.80 V (Figure 12e and 12f). In addition, the superiority of the freestanding electrode was further confirmed by comparing it to the slurry-coating electrode (P–Co_4_N/CNW/CC). Finally, a flexible rechargeable cable-type ZAB using Co_4_N/CNW/CC and a Zn belt as the cathode and anode, respectively, was assembled successfully, achieving a high open-circuit voltage of 1.346 V and outstanding mechanical flexibility.

As discussed in section 3.1.3, filtration is an effective method to prepare freestanding film electrodes. Recently, Wang et al. synthesized a freestanding 3D heterostructure film by filtration of a mixture of Ni–MOF and graphene oxide (GO).^88^ After the subsequent pyrolysis process, Ni–MOF-derived CNT microspheres and 2D reduced GO (rGO) were intertwined to form a flexible freestanding film (Ni@N-HCGHF) (Figure 12g and 12h), directly acting as a binder-free electrode for the ORR, OER, and hydrogen evolution reaction (HER). In addition to the aforementioned advantages of a large specific surface area and high porosity, this design also achieved controlled dopants and a conductive CNT@rGO heterostructure, conductive to highly effective catalytic behavior. As expected, the Ni@N-HCGHF achieved excellent OER and ORR performance with a low overpotential (10 mA cm^–2^) of 260 mV and a high half-wave potential of 0.875 V. This could be attributed to the synergistic effect of the N-doped carbon shell and Ni nanoparticles in the formed heterostructure, as confirmed by the theoretical calculations and experimental results. A ZAB, with the obtained Ni@N-HCGHF and Zn plate as the cathode and anode, respectively, was assembled, achieving a stable open-circuit voltage of 1.49 V (Figure 12i).

Because of the low electrical conductivity and poor electrocatalytic activity, most freestanding pristine MOFs are not suitable as catalytic cathodes for ZABs. To date, extensive efforts have been devoted to developing MOF derivatives for high-performance air cathodes. However, the synthesis conditions of MOF derivatives, such as pyrolysis, are generally complicated and harsh, which could also destroy the original channel structures and active sites. Therefore, it is crucial to develop a moderate strategy to treat pristine MOFs directly acting as a cathode for the ZAB. For example, Lu et al. developed a low-temperature thermal treatment method to introduce linker defects without destroying its structural integrity within the freestanding ZIF as the cathode (D-ZIF) for the ZAB, which improved the electrochemical activity and electron transport rate.^121^ Compared to the ZIF before the low-temperature thermal treatment, the optical color and nanosheet morphology of D-ZIF have hardly changed (Figure 12j). Figure 12k and 12l provide a structural representation of the ZIF and D-ZIF, respectively, in which the low-temperature thermal treatment caused the formation of linker defects. Benefiting from the existence of linker defects, the D-ZIF delivered better ORR performance with a smaller onset potential (0.86 V) and a higher half-wave potential (0.6 V) than the ZIF (Figure 12m). Using the D-ZIF as the cathode, a ZAB was assembled and delivered an acceptable open-circuit voltage (1.38 V) and good cycling stability.

#### 4.3.2 ZN/CBs

**Figure 13:**
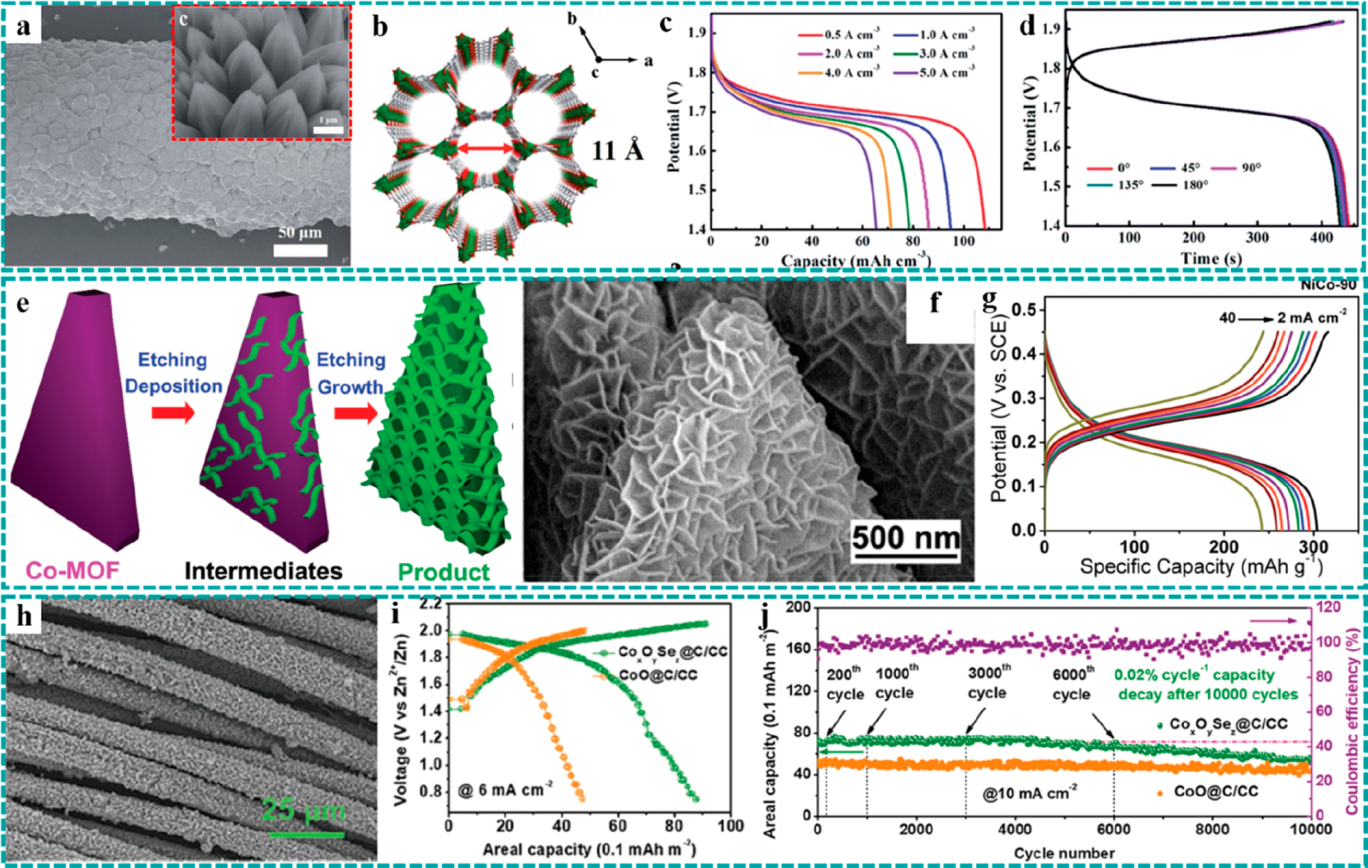
Freestanding MOF-based/derived electrodes for ZN/CBs. (a) SEM images with different resolutions of Ni–MOF-74@CNTF. (b) Crystal structural schematic of Ni–MOF-74. (c) Discharging curves at various current densities and (d) GCD curves at different bending angles of assembled QSS ZNB. Reproduced with permission from ref (72). Copyright 2019 Royal Society of Chemistry. (e) Schematic diagram of the synthetic process of NiCo–DH nanosheet arrays. (f) SEM image and (g) charging/discharging curves at different current densities under a three-electrode system of NiCo–DH. Reproduced with permission from ref (177). Copyright 2019 The Author(s). (h) SEM image of CoSe_2–*x*_@C/CC. Comparison of (i) charging/discharging curves at 6 mA cm^–2^ and (j) long-term cycling stability and corresponding Coulombic efficiency at 10 mA cm^–2^ of CoSe_2–*x*_@C/CC and CoO@C/CC. Reproduced with permission from ref (175). Copyright 2020 Wiley-VCH.

Compared to the ZABs, ZN/CBs could deliver higher discharging voltages and lower polarization as well as being processed by simpler assembly methods, exhibiting huge application potential.^174^ In the ZN/CB battery system, Ni (or Co)-based oxides/hydroxides, as the cathodes, achieve the storage/release of energy through the reversible reaction with OH^–^ in alkaline electrolytes. Due to the excellent electrochemical properties of metallic Zn in alkaline electrolytes, the design and preparation of cathodes largely determined the performance of these batteries. Ni/Co-based freestanding MOFs could provide abundant Ni/Co-based active sites and rapid transport channels for OH^–^, holding ideal application prospects for high-performance cathode materials.^72,175−178^ For example, Yao et al. developed a simple solvothermal method to grow cone-like Ni–MOF-74 with a 1D channel structure on the surface of CNTF directly as the cathode (Ni–MOF-74@CNTF) for ZNBs, as shown in Figure 13a.^72^Figure 13b shows the crystal structure of Ni–MOF-74 with a honeycomb structure in which the 1D channel structure consists of Ni and 2,5-dihydroxyterephthalic acid. In particular, the pore size was ∼11 Å, larger than the diameter of OH^–^ (∼6 Å), which offered a “nanometer highway” for the diffusion of OH^–^. Combining the fast electron transport of the freestanding structure and high mass loading, the Ni–MOF-74 delivered a high volumetric capacity and good rate capability. In addition, owing to the excellent adhesion between Ni–MOF-74 and CNTF, a flexible QSS fiber-shaped ZNB using Ni–MOF@CNTF as the cathode was assembled, delivering a high discharging voltage of ∼1.7 V and a high volumetric capacity (108.5 mAh cm^–3^) (Figure 13c). Moreover, its high volumetric energy density and power density as well as remarkable mechanical flexibility (Figure 13d) demonstrated the application potential of freestanding pristine MOFs for wearable energy storage.

Apart from the direct application as the cathodes for ZN/CBs, freestanding pristine MOFs also provide metal sources and skeletons to construct high-performance binder-free cathode materials with 3D hierarchical structures. For example, Wang et al. reported the nickel–cobalt double hydroxide (NiCo-DH) micronanosheet arrays with hierarchical structures derived from Co–MOF grown on nickel foam by a one-step etching–deposition–growth process, as illustrated in Figure 13e.^177^ After the transformation, the skeleton structure of Co–MOF could be well preserved and the NiCo-DH nanosheets were uniformly covered on their surface (Figure 13f), exposing more accessible active sites and channel structures. Benefiting from these advantages, the NiCo–DH electrode delivered a high capacity of 303.6 mAh g^–1^ at 2 mA cm^–2^ and still maintained 80% of the initial capacity as the current density increased to 40 mA cm^–2^ (Figure 13g), implying remarkable rate performance. Moreover, the assembled ZNB achieved a high discharging voltage of ∼1.65 V and low voltage hysteresis of ∼0.068 V at 0.5 mA cm^–2^, further demonstrating the excellent electrochemical performance of the NiCo–DH electrode. Nevertheless, the relatively low capacity retention (∼73%) of the assembled ZNB after 850 cycles could not satisfy the expected requirement of high-performance energy storage. To achieve a long-cycle lifespan for ZN/CBs, Zhi et al. reported a selenization method to prepare freestanding leaf-like CoSe_2–*x*_@C nanosheet arrays derived from ZIF-67 grown on CC (Figure 13h), directly serving as the cathode (CoSe_2–*x*_@C/CC) for alkaline Zn–Co batteries.^175^ Notably, the doping of Se could maintain a comparatively stable Co^3+^-rich state in the active materials, which effectively restrained thermodynamic degradation transformation of surplus Co^3+^ to equilibrium Co^2+^. Compared to the redox pair Co^3+^/Co^2+^, the Co^4+^/Co^3+^ pair provided a higher discharging voltage and had the contribution to the capacity. Therefore, the CoSe_2–*x*_@C/CC delivered a high output voltage of 1.8–1.9 V and greater areal capacity than the CoO@C/CC (Figure 13i). Moreover, benefiting from the stabilizing effect of Se on Co^3+^, the freestanding CoSe_2–*x*_@C/CC exhibited excellent cycle stability with a 0.02% cycle^–1^ capacity decay (10 000 cycles) at a current density of 10 mA cm^–2^ (Figure 13j).

#### 4.3.3 ZIBs

**Figure 14:**
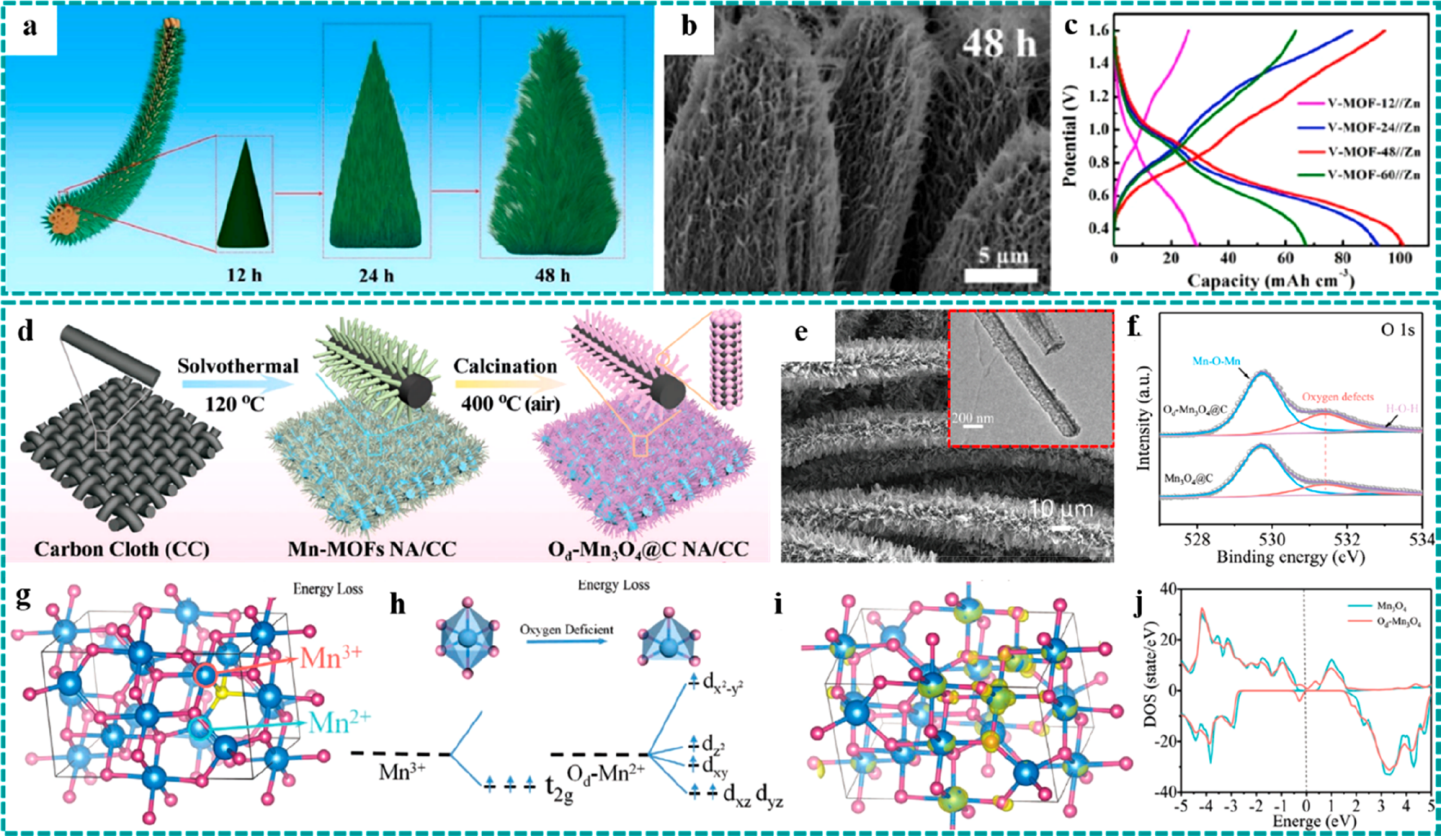
Freestanding MOF-based/derived electrodes for ZIBs. (a) Schematic illustration of the synthetic process. (b) SEM image of V–MOF@CNTF with hierarchical structures. (c) Charging/discharging curves at 0.1 A cm^–3^ of V–MOF@CNTF with different reaction times (12, 24, 48, and 60 h). Reproduced with permission from ref (120). Copyright 2019 Elsevier. (d) Preparation process and (e) SEM and TEM images of O_d_-Mn_3_O_4_@C NA/CC. (f) High-resolution O 1s XPS spectra of O_d_-Mn_3_O_4_@C. (g) Supercell model of Mn_3_O_4_. (h) Mn–O octahedral and pyramidal crystal fields and d-orbital splitting configurations. (i) Electron density difference of O_d_-Mn_3_O_4_. (j) TDOS of the Mn_3_O_4_ and O_d_-Mn_3_O_4_ bulk phase. Reproduced with permission from ref (109). Copyright 2020 Wiley-VCH.

Unlike the alkaline electrolytes adopted by the ZABs and ZN/CBs, ZIBs use largely neutral or weak acid aqueous solutions with higher safety as the electrolytes, which could effectively prevent the electrolytes from corroding the current collectors or packaging materials and avoiding the safety accidents caused by electrolyte leakage.^179,180^ In addition, the Zn anode exhibits a high theoretical capacity of 820 mAh g^–1^, low potential (0.76 V vs. SHE), and low toxicity, thereby having attracted extensive efforts devoted to the development of ZIBs. To match the excellent electrochemical performance of the Zn anode, a number of materials have been studied as cathodes for ZIBs, including transition metal-based compounds, PAB-based materials, and organic materials.^179,181^ Among them, transition metal-based compounds display great potential as promising cathodes for ZIBs owing to their relatively high theoretical capacities. Nevertheless, the poor electric conductivity, small surface area, and low structural stability of these traditional transition metal-based compounds have become the bottleneck as high-performance cathodes for ZIBs. The freestanding MOFs can provide an effective solution to construct nanostructured cathode materials with a large specific surface area and high porosity, thereby endowing assembled ZIBs with a high specific capacity and excellent rate performance. For example, Yao et al. developed a self-sacrificed method to prepare 3D V-based MOF nanowire-bundle arrays on the surface of CNTF by adjusting the reaction time, as illustrated in Figure 14a, directly serving as the freestanding cathodes (V–MOF@CNTF) in ZIBs.^120^ With the prolongation of the reaction time, the initial dense structure transformed into hierarchical nanowire-bundle arrays (reaction time of 48 h, Figure 14b), which not only greatly enriched the accessible active sites for enhanced volumetric capacity but also shortened the ion diffusion distance for good rate performance. Thus, the charging/discharging curves of V–MOF@CNTF with different reaction times at 0.1 A cm^–3^ showed that V–MOF-48 (48 referred to the reaction time, h) delivered a high volumetric capacity of 101.8 mAh cm^–3^. After 48 h, the nanostructure of V–MOF gradually became fragile as the reaction time continued to extend, leading to a degree of decay in cycle stability. Moreover, benefiting from the freestanding electrode and the unique hierarchical structure, the V–MOF-48@CNTF displayed outstanding rate performance and maintained a high volumetric capacity of 65.5 mAh cm^–3^ as the current density increased to 5.0 A cm^–3^ (Figure 14c).

Although freestanding V–MOF possesses largely ordered channel structures for superior rate performance, the existence of organic linkers with a large mass ratio would lead to a relatively low specific/volumetric capacity. In contrast, certain MOF derivatives obtained by pyrolysis can eliminate the issue of massive organic linkers by forming oxides or carbon-based materials and are usually transformed to porous nanostructures for abundant active sites and a short ion diffusion distance.^61,182^ Similarly, the amorphous carbon derived from organic linkers could improve the electrical conductivity and cycle stability owing to the accommodation of stress during the insertion/desertion of Zn^2+^. Therefore, extensive efforts have been devoted to constructing freestanding MOF derivatives as cathodes for ZIBs.^109,183,184^ For example, Wang et al. reported MOF-derived bulk oxygen defect Mn_3_O_4_@C nanorod arrays (O_d_-Mn_3_O_4_@C NA/CC) as the freestanding cathode for ZIBs in which O_d_-Mn_3_O_4_@C NA/CC was synthesized by the solvothermal method and sequent calcination, as illustrated in Figure 14d.^109^Figure 14e shows the corresponding SEM and TEM images, indicating that the nanorod arrays with porous structures were uniformly dispersed on the surface of carbon fibers. During the calcination process, the formed carbon would carry off lattice oxygen, thereby leading to the generation of plenty of oxygen defects (Figure 14f), which were expected to improve the conductivity and activity of the electrode materials. Combined with the continuous carbon skeleton and freestanding structure for the enhanced conductivity, the O_d_-Mn_3_O_4_@C NA/CC displayed a remarkable capacity of 396.2 mAh g^–1^ (0.2 A g^–1^) and still maintained 36.1% of the initial capacity as the current density was increased to 5 A g^–1^, implying the excellent storage performance of Zn^2+^ and rate capability. Besides, the total density of state (TDOS) of the O_d_-Mn_3_O_4_@C NA/CC was calculated to investigate the mechanism of oxygen defects enhancing conductivity. As shown in Figure 14g and 14h, losing the O atom made the (MnO_6_) octahedron transform into a pyramidal crystal field, improving the conductivity according to the theory of d-orbital level splitting. The result is further demonstrated by the electron density difference in Figure 14i. Figure 14j shows that the band gaps of Mn_3_O_4_ and O_d_-Mn_3_O_4_ were 0.6 and 0 eV, proving that the existence of oxygen defects could effectively improve the conductivity.

### 4.4 Ni–Fe Batteries (NFBs)

**Figure 15:**
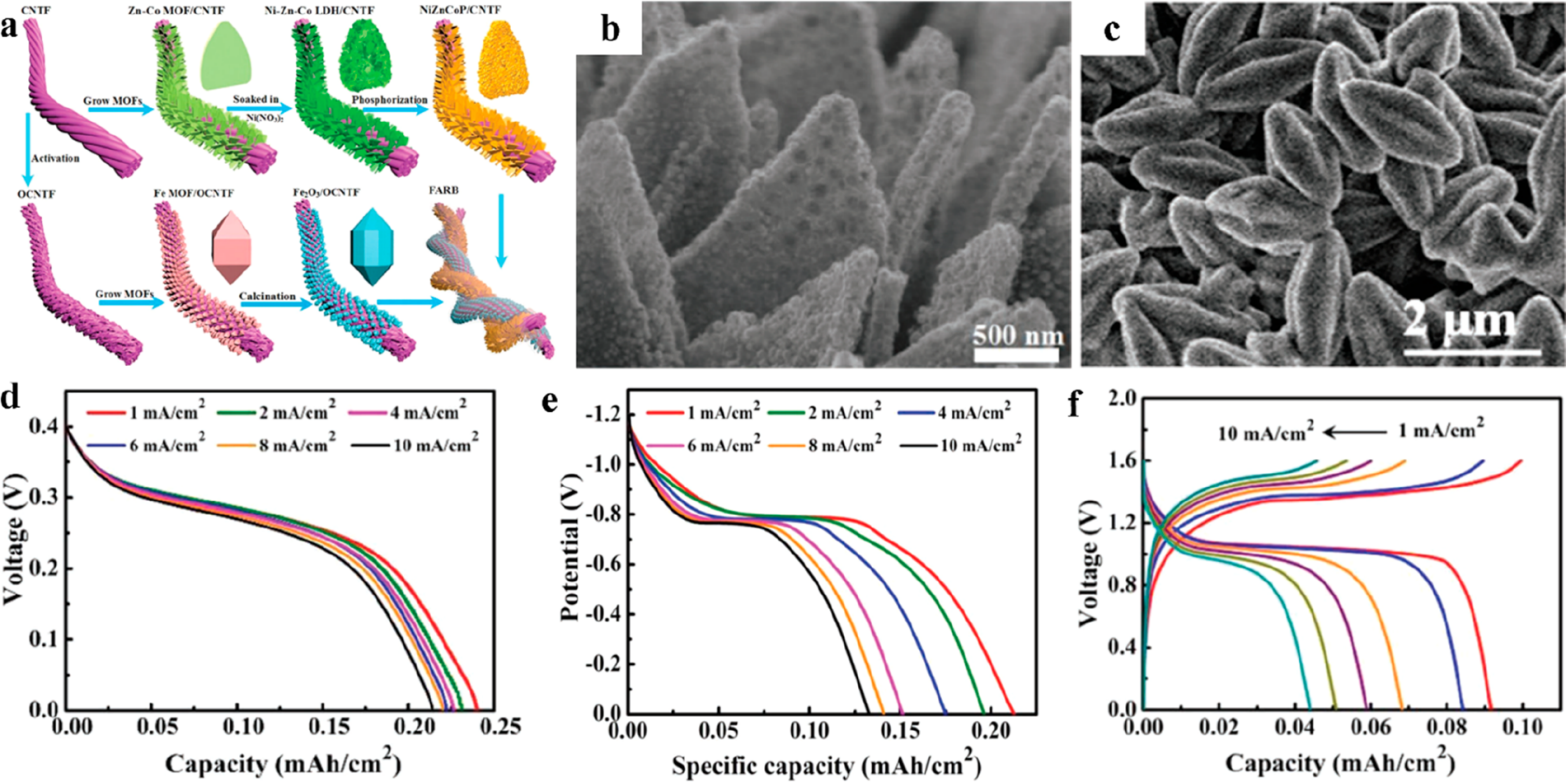
Freestanding MOF derivative materials as electrodes for NFBs. (a) Preparation process of the fiber-shaped NFB. SEM images of (b) NiZnCoP/CNTF and (c) Fe_2_O_3_/CNTF. Discharging curves at different current densities from 1 to 10 mA cm^–2^ of (d) NiZnCoP/CNTF and (e) Fe_2_O_3_/CNTF. (f) Charging/discharging curves at various current densities of the assembled device. Reproduced with permission from ref (186). Copyright 2018 The Author(s).

In addition to ZABs and ZN/CBs, Ni–Fe batteries also have an important place in aqueous alkaline batteries owing to the abundant Ni/Fe source, low cost, and stable output voltage.^182,185^ However, the low rate capability and poor cycle life limit their further development of NFBs, resulting from the intrinsic sluggish electron and ion transport of semiconducting materials. Therefore, freestanding MOF derivatives with high conductivity and stability help address these issues to achieve high-performance NFBs. Recently, a fiber-shaped QSS NFB based on all-MOF-derived electrode materials was constructed, as illustrated in Figure 15a.^186^ Thus, the freestanding MOF-derived NiZnCoP grown on CNTF was synthesized by mild ion exchange and subsequent phosphorization treatment as the cathode (NiZnCoP/CNTF) and the freestanding MOF-derived Fe_2_O_3_ grown on the oxidized CNTF (OCNTF) was prepared through a simple thermal treatment as the anode (Fe_2_O_3_/OCNTF) for NFBs. For the NiZnCoP/CNTF, it could still maintain the original nanowall arrays and exhibited high porosity (Figure 15b), which provided a large accessible active area and short diffusion distance for a high specific capacity and rate capability. As expected, the NiZnCoP/CNTF delivered a high areal capacity of 0.24 mAh cm^–2^ at 1 mA cm^–2^ and an outstanding rate performance (0.21 mAh cm^–2^ at 10 mA cm^–2^) (Figure 15d). For Fe-based anodes, the SEM image (Figure 15c) showed that the spindle-like Fe_2_O_3_ was uniformly distributed on the surface of CNTF in which the internal space among nanoparticles was conductive to the fast diffusion of electrolyte ions (OH^–^). Figure 15e shows the discharging curves of Fe_2_O_3_/CNTF at various current densities, which gives a stable output voltage of ∼ −0.8 V and a high areal capacity of 0.21 mAh cm^–2^ at 1 mA cm^–2^. On the basis of the remarkable electrochemical performance of the cathode and anode, the assembled fiber-shaped NFB delivered a stable discharge platform of ∼1.05 V and small voltage hysteresis of ∼0.3 V at 1 mA cm^–2^ (Figure 15f), indicating the good conductivity and fast electrochemical kinetics. Moreover, the device also achieved a high volumetric energy density (30.61 mWh cm^–3^) and power density (3339.7 mW cm^–3^) as well as good mechanical flexibility, demonstrating the unique advantages of the freestanding MOF derivatives for flexible NFBs.

### 4.5 Supercapacitors

Compared to batteries with high energy density, supercapacitors, as a class of energy storage devices with high power density, have received widespread attention on account of their high safety, fast charging/discharging characteristics, and excellent cycle life.^187−189^ However, the relatively low energy density becomes a significant obstacle to the wide applications of supercapacitors. On the basis of the energy storage mechanisms of the electric double-layer capacitor (EDLC) and pseudocapacitor, various design strategies of electrode preparation have been adopted to achieve an improvement of energy densities while maintaining high power densities in which the general qualities of promising electrodes should contain large accessible surface areas, high porosity, good electron transfer capability, and abundant redox-active sites.^190^ As emerging porous materials, freestanding MOFs, consisting of pristine MOFs and MOF derivatives, have been proven to be ideal electrode materials for supercapacitors, as summarized in Table S5.

#### 4.5.1 Freestanding MOF Derivative Electrodes for Supercapacitors

**Figure 16:**
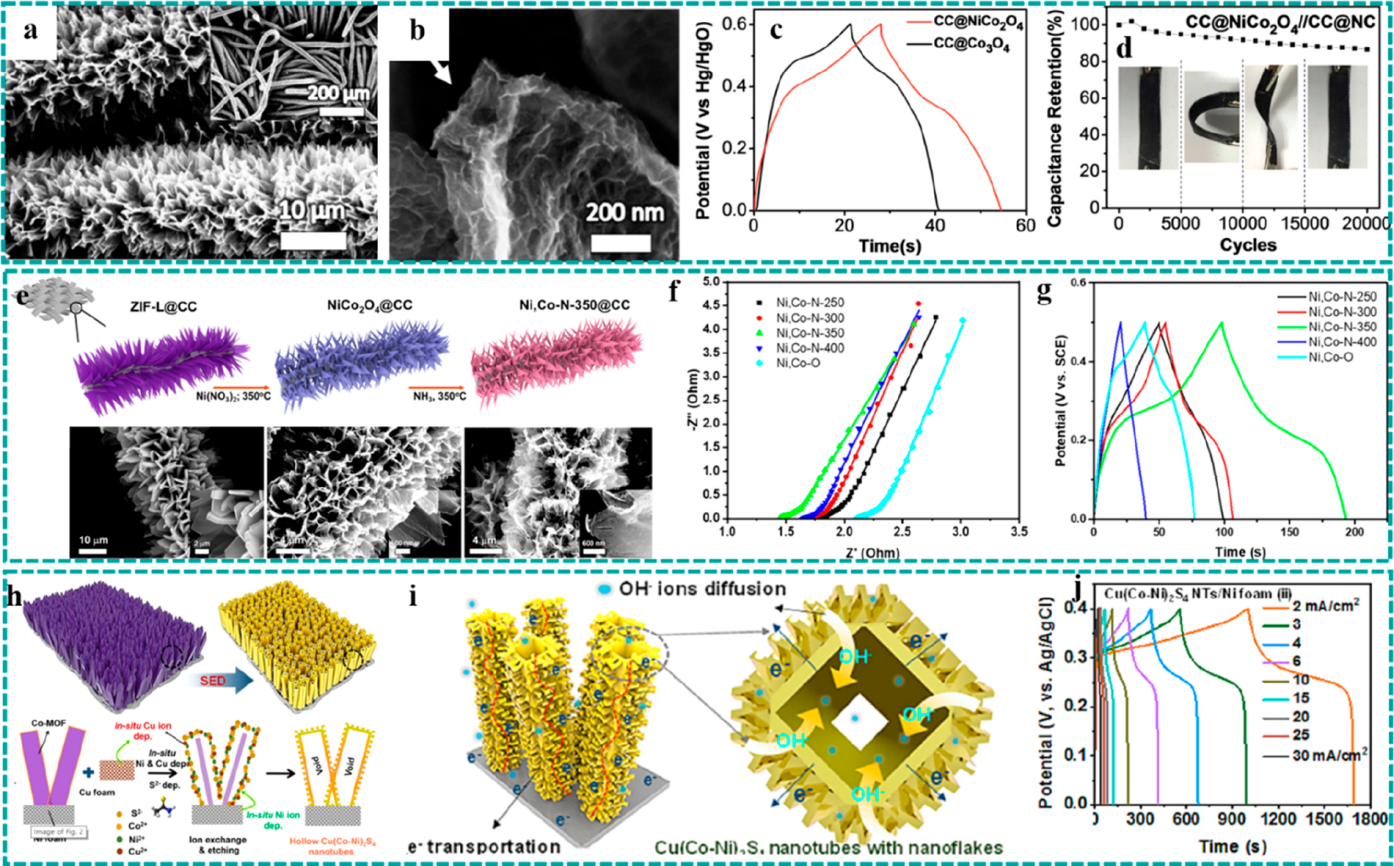
Freestanding MOF derivative electrodes applied in supercapacitors. (a and b) SEM images of CC@NiCo_2_O_4_ with different resolution. (c) Comparison of GCD curves of electrode materials with and without introduction of Ni. (d) Long-term cycling stability test of the assembled device at 5 mA cm^–2^ (inset: optical images of the flexible device under various bending states). Reproduced with permission from ref (49). Copyright 2017 Wiley-VCH. (e) Schematic displaying the synthetic process and corresponding SEM images at different reaction steps of Ni,Co–N-350@CC. Comparisons of (f) Nyquist plots and (g) GCD curves of electrode materials with different nitridation temperatures. Reproduced with permission from ref (65). Copyright 2018 American Chemical Society. (h) Schematic of the formation mechanism of hollow Cu(Co–Ni)_2_S_4_ nanotubes. (i) Schematic diagram of ion diffusion and electron transport and (j) GCD curves at various current densities of Cu(Co–Ni)_2_S_4_ NTs/NF. Reproduced with permission from ref (114). Copyright 2021 Elsevier.

Due to the intrinsically inferior electrical conductivity, it is difficult for traditional pristine MOFs to directly serve as electrodes for supercapacitors. By comparison, transition metal oxides derived from MOFs are well known as a class of pseudocapacitive materials.^49,65,66,87,114,191−195^ Wang et al. reported an innovative method to grow 2D MOF-derived hollow NiCo_2_O_4_ nanowall arrays on flexible CC by an ion-exchange and etching process and subsequent thermal treatment (Figure 16a and 16b), directly serving as a freestanding electrode (CC@NiCo_2_O_4_) for supercapacitors.^49^ The freestanding MOF derivative electrodes exhibit numerous advantages for electrochemical capacitance, including (i) high porosity for fast ion diffusion and transport, (ii) freestanding electrode design for excellent mechanical stability and electric conductivity, and (iii) hollow 2D nanostructures for a large surface area and short diffusion distance. Figure 16c compares the galvanostatic charge–discharge (GCD) curves of CC@ NiCo_2_O_4_ and CC@ Co_3_O_4_ at 10 mA cm^–2^, indicating that the introduction of Ni could improve the specific capacitance of the electrode. Moreover, a flexible asymmetric supercapacitor using NiCo_2_O_4_ nanowalls and nitrogen-doped carbon flakes grown on CC was assembled, maintaining 86.7% of the initial capacity under different bending states after 2000 cycles at a current density of 5 mA cm^–2^ (Figure 16d). The excellent cycle stability and mechanical flexibility further demonstrated the great potential of freestanding MOF-based/derived electrodes for high-performance flexible supercapacitors. Nevertheless, metal oxide/hydroxide compounds possess relatively low electrical conductivity, further resulting in limited power density. To address this issue, the obtained NiCo_2_O_4_ was further nitridized by thermal treatment in an NH_3_ atmosphere to prepare a 2D heterostructure composed of Ni-doped Co and Co_2_N, and the alloys and metal nitrides have been proven to enhance the electrical conductivity and electrochemical activities.^65^ As shown in Figure 16e, the structure of the 2D nanosheet could be well retained after the nitridation process, exposing large surface areas and dense active sites. The enhanced conductivity was demonstrated by the Nyquist plots (Figure 16f) in which the doping of the N element could obviously improve the conductivity of NiCo_2_O_4_. Besides, the effect of the nitriding temperature on the electrode material was also studied, implying that the freestanding electrode material at 350 °C could achieve an optimal conductivity. Moreover, the corresponding electrode delivered the largest specific capacitance compared with the other electrodes prepared at different nitridation temperatures (Figure 16g). The high conductivity and excellent electrochemical properties were mainly attributed to the metal–metal nitride heterostructure and MOF-derived porous structure.

In addition to the thermal treatment, the solvothermal/hydrothermal method is an effective strategy for preparing MOF derivatives with hierarchical structures, directly serving as freestanding electrodes for supercapacitors. Compared to the thermal treatment with high temperature, solvothermal/hydrothermal methods with relatively mild temperature prevent the precursors from collapsing and falling off due to a high temperature and could form a more abundant and complex morphological structure, which is beneficial to exposing large accessible active areas. For example, Yu et al. developed a solvothermal method to synthesize MOF-derived ternary Cu(Co–Ni)_2_S_4_ nanotubes with the hierarchical structure on NF serving as the freestanding electrode (Cu(Co–Ni)_2_S_4_ NTs/NF) for supercapacitors.^114^ As shown in Figure 16h, the formation of Cu(Co–Ni)_2_S_4_ NTs/NF involved a synchronous etching and doping process consisting of two simultaneous processes: the etching of Co–MOF and the regrowth with the doping of the Cu element. The obtained Cu(Co–Ni)_2_S_4_ NTs/NF with a hierarchical hollow structure exhibited numerous benefits, as illustrated in Figure 16i, including (i) the hierarchical hollow structure increased the electroactive surface and shortened the diffusion distance of OH^–^, improving the specific capacity and redox kinetics of electrode, (ii) Cu doping enhanced the electrical conductivity of the electrode for good rate capability, (iii) the freestanding design decreased the internal resistance of the electrode, and (iv) the surrounding nanoflakes were conductive to keep the overall stability. Benefiting from these remarkable features, Cu(Co–Ni)_2_S_4_ NTs/NF delivered an areal capacity of 382.1 μAh cm^–2^ at 2 mA cm^–2^ and retained a high capacity of 200 μAh cm^–2^ as the current density increased 15 times (Figure 16j), indicating excellent rate performance.

#### 4.5.2 Freestanding Pristine MOF Electrodes for Supercapacitors

**Figure 17:**
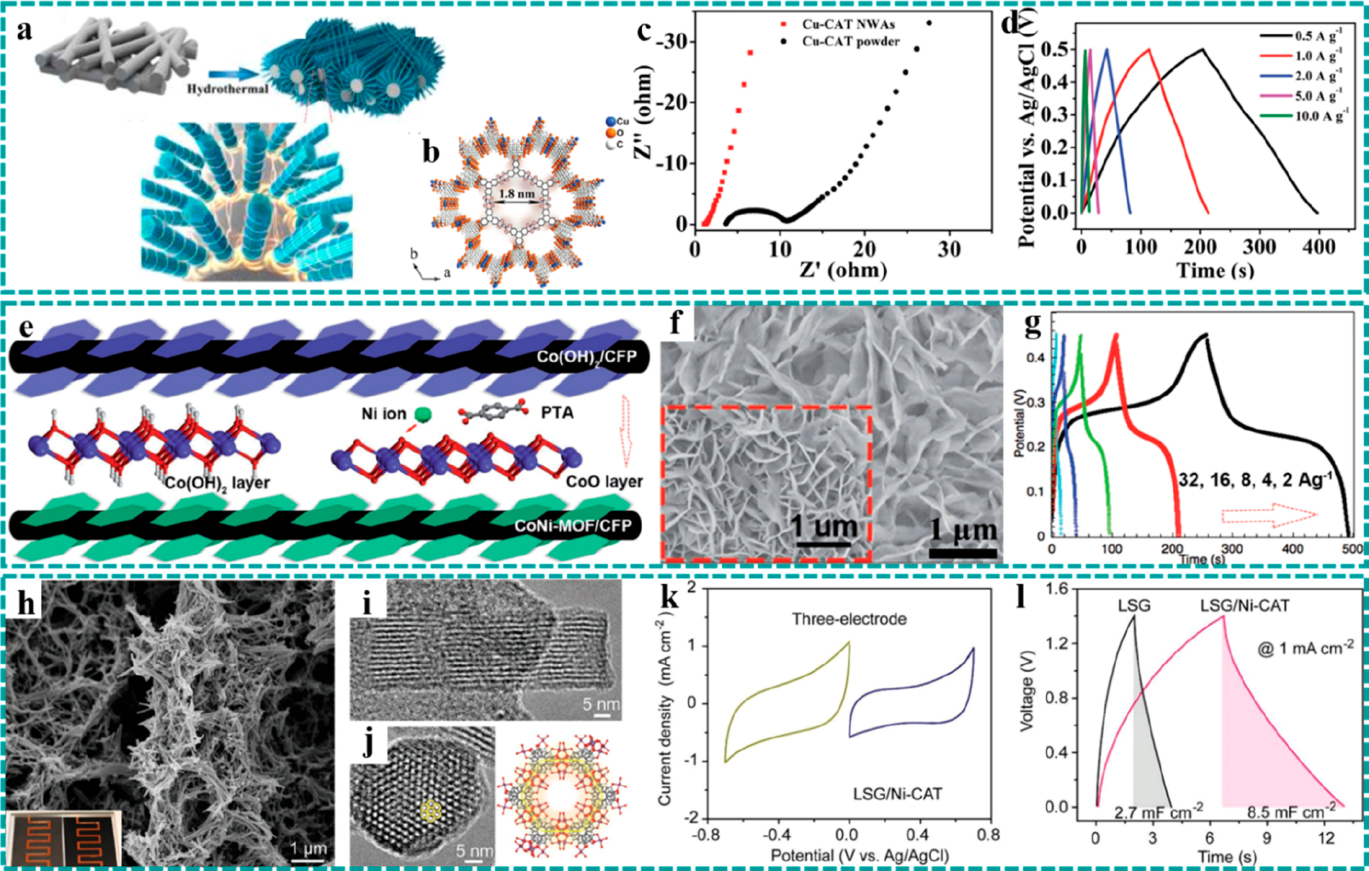
Freestanding pristine MOF electrodes for supercapacitors. (a) Schematic showing a one-step method for the preparation of Cu–CAT NWAs. (b) Crystal structure of Cu–CAT. (c) Comparison of Nyquist plots of the freestanding Cu–CAT NWAs electrode and Cu–CAT powder-form electrode. (d) GCD curves of Cu–CAT NWAs. Reproduced with permission from ref (75). Copyright 2017 Wiley-VCH. (e) Schematic diagram of conversion preparation from Co(OH)_2_ to CoNi–MOF. (f) SEM images of CoNi–MOF and Co(OH)_2_ (inset). (g) GCD curves at various current densities of CoNi–MOF/CFP. Reproduced with permission from ref (86). Copyright 2018 Wiley-VCH. (h) SEM image of LSG/Ni–MOF (inset: optical images of LSG before and after growth of Ni–MOF). (i and j) High-resolution TEM images and corresponding crystal structure of Ni–MOF. (k) CV curves of LSG/Ni–MOF as both the positive and the negative electrodes. (l) Comparison of GCD curves of LSG/Ni–MOF and LSG. Reproduced with permission from ref (73). Copyright 2019 Wiley-VCH.

Although the freestanding MOF derivatives obtained by pyrolysis or ion exchange overcome some of the inherent limitations of poor electrical conductivity and deliver good electrochemical properties in supercapacitors, they can suffer from the loss of the regular channel structure and reduction in abundant reactive sites of pristine MOFs and thereby may not perfectly present their unique advantages for electrochemical capacitance.^196,197^ In 2017, Dincǎ et al. reported a conductive MOF, Ni_3_(2,3,6,7,10,11-hexaiminotriphenylene)_2_, serving as the binder-free powder-form electrode for EDLCs, which stimulated considerable interest in the development of conductive MOFs.^197^ To achieve higher specific capacitances and better rate performance, extensive efforts have been devoted to the nanostructure design of freestanding pristine MOFs, including nanowires, nanosheets, etc.^44,70,73,75,86,198−202^ In the same year, Xu et al. synthesized conductive MOF (Cu–CAT) nanowire arrays (NWAs) on CFP by a simple hydrothermal method (Figures 17a), directly serving as the flexible freestanding electrode for supercapacitors.^75^ The excellent conductivity of the target electrode was mainly attributed to the following three factors. (i) The high electrical conductivity of Cu–CAT. Cu–CAT has a relatively high inherent electrical conductivity (20 S m^–1^) because of the effective orbital overlap between Cu^2+^ and the 2,3,6,7,10,11-hexahydroxytriphenylene (HHTP) ligands, endowing the electrode with fast charge transport. (ii) The unique 1D channel structure. The crystal structure viewed along the *c* axis is shown in Figure 17b, in which the *ab* plane consists of Cu^2+^ and HHTP ligands. The Cu–CAT with a honeycomb-like structure has 1D channels along the *c* axis with a diameter of ∼1.8 nm, coinciding with the high-resolution TEM result (1.83 nm). Moreover, the TEM image further exhibited the extension of the 1D channel of Cu–CAT that was consistent with the growth direction of the nanowire, which could facilitate the rapid transport of electrolyte ions for fast reaction kinetics. (iii) The design of freestanding electrodes. Compared with the powder electrode, the advantages of the freestanding Cu–CAT NWAs electrode were clearly demonstrated by the electrochemical impedance spectra (Figure 17c) in which the freestanding electrode delivered much lower ohmic resistance (1.2 Ω) and charge transfer resistance (0.5 Ω) than those of the powder electrodes (3.6 and 7.4 Ω). Benefiting from the uniform NWAs and excellent conductivity, the freestanding electrode achieved a specific capacitance (202 F g^–1^ at 0.5 A g^–1^) and a remarkable rate capability (66% retention of the initial capacitance as the current density increased to 2 A g^–1^) as shown in Figure 17d.

Despite the remarkable properties of freestanding conductive MOFs when applied in supercapacitors, it is difficult for most of the known conductive MOFs to grow on the surface of conductive substrates via an in situ growth method. Therefore, developing a rational strategy to construct freestanding conductive MOFs as electrodes is imperative for high-performance supercapacitors. As depicted in section 4.5.1, freestanding pristine MOFs are common templates to synthesize the corresponding metal oxides/hydroxides as binder-free electrodes for supercapacitors, regarded as a regular design strategy. For example, Zheng et al. reported an inverted design strategy where Co(OH)_2_ nanosheets grown on CFP were used as the template and precursor to synthesize CoNi–MOF as freestanding electrodes (CoNi–MOF/CFP) for supercapacitors, as illustrated in Figure 17e, in which the Ni atom replaced the site of the H atom in Co(OH)_2_ and participated in the formation of CoNi–MOF nanosheets.^86^Figure 17f and the inset show the SEM images of CoNi–MOF and Co(OH)_2_, indicating that the original vertically oriented nanosheet arrays were well reserved after the transformation. Besides, introduction of faradaic pseudocapacitance could greatly increase the specific capacitance of EDLC, thereby achieving high energy density supercapacitors. Combined with the excellent conductivity of the freestanding electrode and conductive MOF, a high specific capacitance of 1044 F g^–1^ at a current density of 2 A g^–1^ and an excellent capacitance retention of 54.5% at 32 A g^–1^ were achieved (Figure 17g), implying remarkable rate performance. This work made full use of the advantages of metal hydroxides that were easy to grow on conductive substrates and the strength of different chemical bonds, thereby unlocking an inverted design strategy to construct freestanding conductive MOFs as electrodes for supercapacitors.

In addition, freestanding conductive MOFs have several exceptional advantages for electrochemical microsupercapacitors (MSCs), which are the products formed in response to the rapid development of portable electronic devices. As for the powder-form conductive MOFs, thermal evaporation/sputtering and etching processes are usually required for the construction of MSCs, which may destroy/damage the origin channel structures and reduce active sites. In contrast, Alshareef et al. utilized polyimide (PI) film as the substrate and carbon source to prepare the 3D laser-scribed graphene (LSG) and synthesized 1D conductive Ni–catecholate (Ni–CAT) MOF nanorods on the surface of LSG, serving as the electrodes (LSG/Ni–MOF) for MSCs, and the original structure and morphology were well reserved.^73^ The SEM image showed that the 1D Ni–CAT MOF nanorods uniformly covered the surface of 3D LSG, and the optical color of the electrodes became darker after growth (Figure 17h). A homogeneous pore size (∼1.2 nm) and ordered channels along the growth direction of nanorods were demonstrated by the TEM studies (Figure 17i and 17j), endowing the electrode materials with faster ion diffusion and transmission. Moreover, the freestanding LSG/Ni–MOF electrode could serve as both the positive and the negative electrodes with potential windows from −0.7 to 0 V and 0 to 0.7 V, respectively, as shown in Figure 17k. On the basis of this character, a symmetric MSC was assembled using the LSG/Ni–MOF as the positive and negative electrodes. Compared with the pure LSG, the loading of Ni–CAT delivered an obvious improvement on the specific areal capacitance, as shown in Figure 17l.

## 5 Freestanding MOF-Based/Derived Electrodes for Energy Conversion

**Figure 18:**
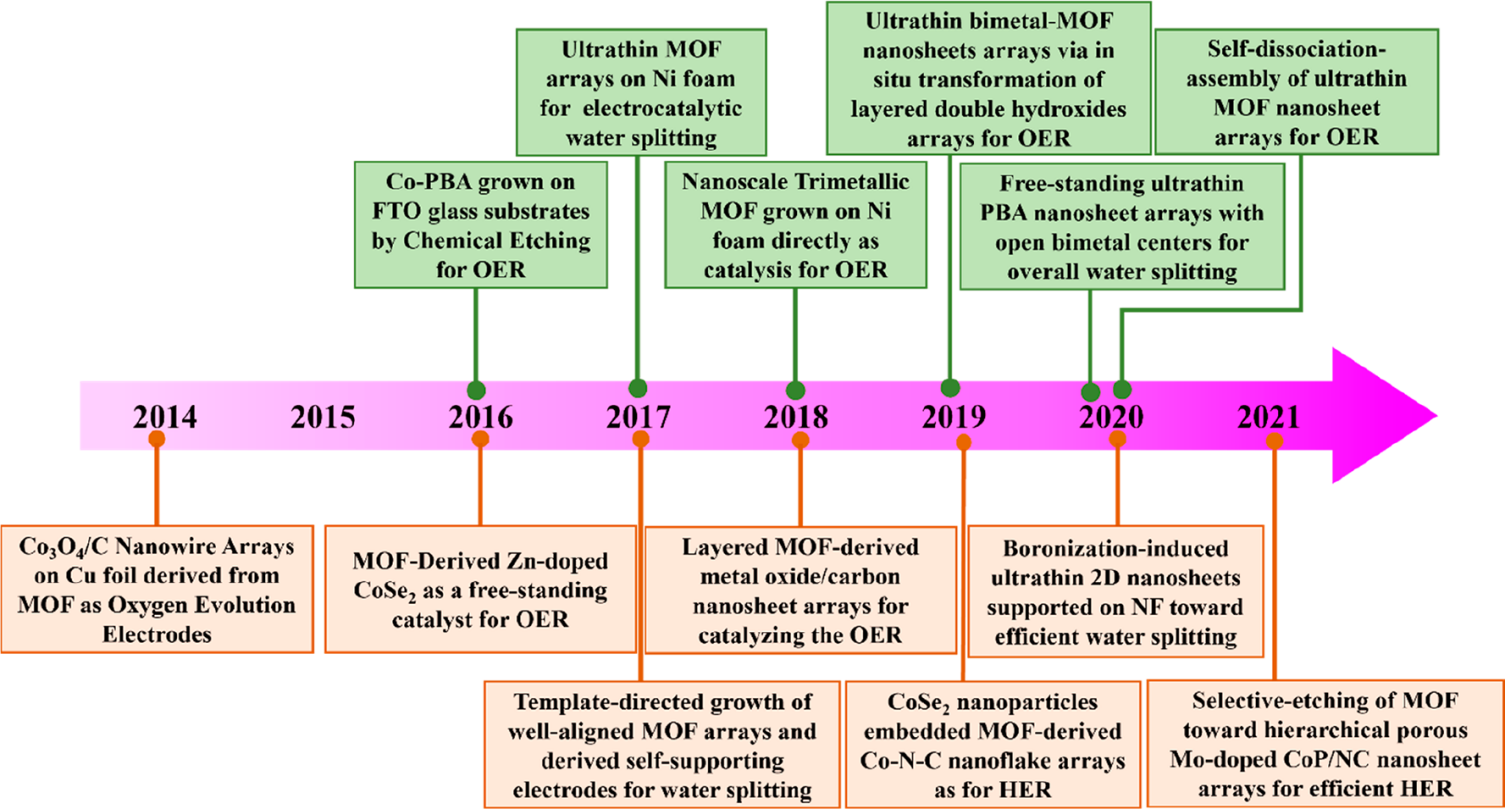
Timeline of freestanding MOF-based/derived electrodes for the HER and OER.

In the past couple of decades, electrochemical water splitting, as a sustainable and environmentally friendly energy conversion technology, has been regarded as an efficient pathway to achieve a low-carbon-emission future, which mainly consists of two important electrochemical reactions: the HER and OER.^203,204^ However, the sluggish reaction kinetics often lead to the inefficiency of the HER and OER because of the high overpotential. To overcome the barrier, rational design and preparation of electrocatalysts would be crucial to lower the overpotential for the high-efficiency catalytic reactions. Despite the fact that noble metals (e.g., Ir and Ru) have delivered excellent catalytic performance, the high cost and scarce resources severely limit their application in the future.^205^ In contrast, freestanding MOF-based/derived electrodes with low cost offer the favorable properties that promising electrocatalysts should possess, including (i) a large specific surface area and ordered channel structure, (ii) adjustable metal clusters and multifunctional linkers for various metal-based, carbon-based, and nitrogen-based reactive sites, and (iii) high electrical conductivity for fast reaction kinetics. Therefore, extensive efforts have recently been devoted to the design and preparation of freestanding MOF-based/derived electrodes as highly efficient electrocatalysts for the HER and OER, as shown in Figure 18.^64,74,76,77,82,83,116,206−211^ In addition, Table S6 summarizes the work using freestanding MOFs and their derivatives as electrocatalysts for the HER and OER.

### 5.1 Freestanding MOF-Based/Derived Electrodes for the OER

#### 5.1.1 Freestanding MOF Derivative Electrodes for the OER

**Figure 19:**
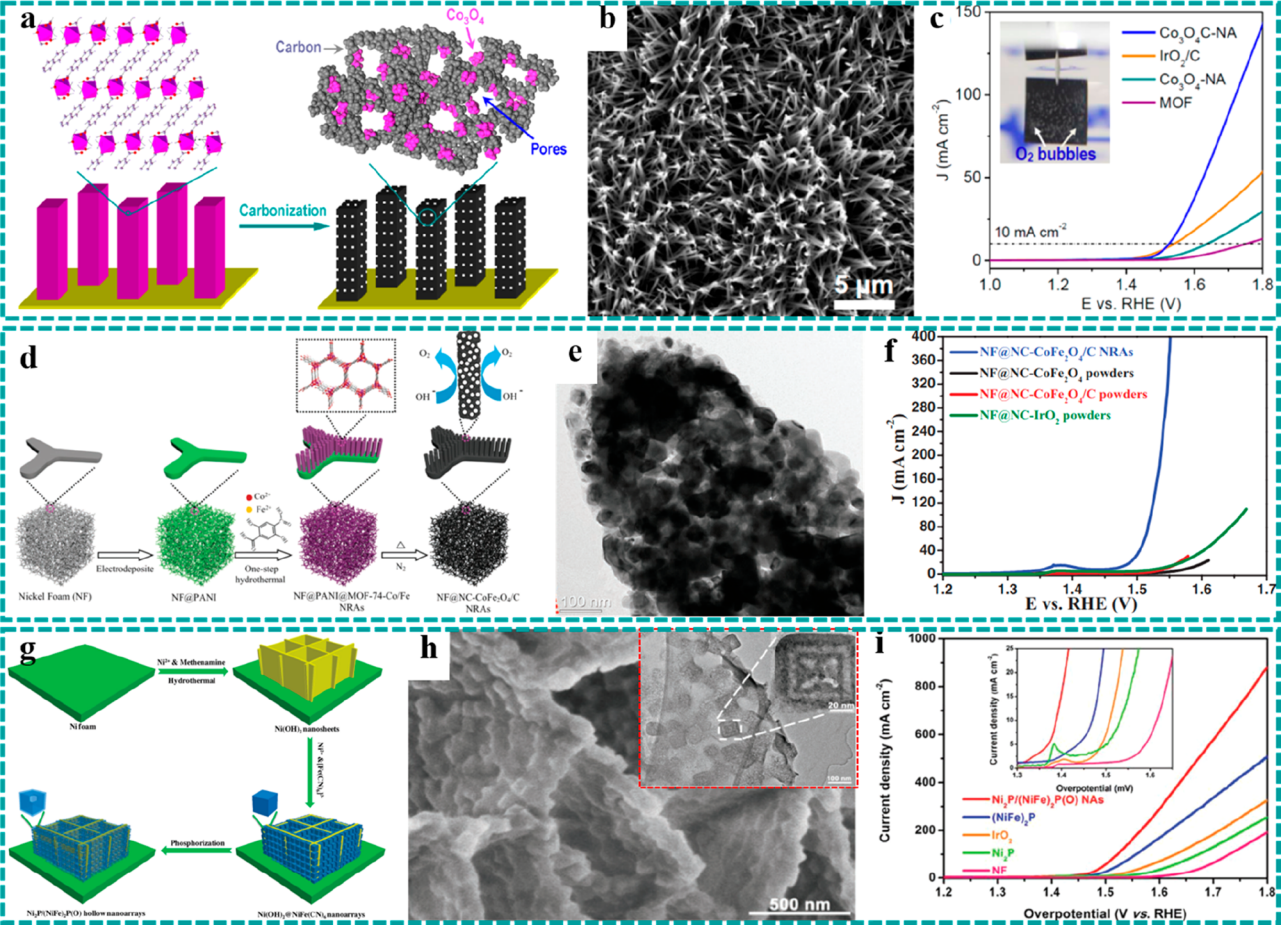
Freestanding MOF derivative electrodes for the OER. (a) Preparation schematic and (b) SEM image of Co_3_O_4_C-NA. (c) LSV curves at 0.5 mV s^–1^ of Co_3_O_4_C-NA and the control groups for the OER. Reproduced with permission from ref (206). Copyright 2014 American Chemical Society. (d) Schematic of the synthetic process and (e) TEM image of NF@NC–CoFe_2_O_4_/C NRAs. (f) LSV curves of NF@NC–CoFe_2_O_4_/C NRAs and the control groups for the OER. Reproduced with permission from ref (63). Copyright 2017 Wiley-VCH. (g) Schematic displaying the fabrication process and (h) SEM and TEM images of Ni_2_P/(NiFe)_2_P(O) NAs. (i) LSV curves at 0.5 mV s^–1^ of Ni_2_P/(NiFe)_2_P(O) NAs and the control groups. Reproduced with permission from ref (216). Copyright 2018 Wiley-VCH.

The OER is a key reaction in several energy conversion and storage devices, such as electrochemical water splitting, metal–air batteries, etc.^212,213^ Thus, the onset potential, overpotential (at a current density of 10 mA cm^–2^), and Tafel slope are important parameters in assessing the OER performance offered by catalysts. Freestanding MOF derivatives have been investigated among the OER catalysts, which are shown to provide advantages to improve the efficiency of electrocatalysts.^63,206−208,214−219^ They can be made into various nanostructured arrays and possess abundant metal/carbon-based active sites, a high level of porosity, and strong adhesion between active materials and conductive substrates. For example, in 2014, Qiao et al. first reported MOF-derived Co_3_O_4_-carbon porous nanowire arrays grown on CF as the freestanding OER catalyst (Co_3_O_4_C-NA).^206^ As shown in Figure 19a, after the carbonization process, the Co_3_O_4_ nanoparticles were shown to uniformly disperse in the continuous carbon-based network with porous structures, which promoted the accessibility of active sites. The SEM image in Figure 19b further showed that the 1D nanowire arrays were well maintained, indicating the excellent stability of the freestanding structure. Figure 19c shows the polarization curves of Co_3_O_4_C-NA, IrO_2_/C, Co_3_O_4_–NA, and the original MOF in an O_2_-saturated 0.1 M KOH electrolyte in which Co_3_O_4_C-NA delivered a low onset potential of ∼1.47 V. The excellent OER catalytic efficiency could be attributed to the following points: (i) a strong combination between Co_3_O_4_ particles and carbon-based networks promoted the charge transfer efficiency and catalytic stability, (ii) the vertical 1D nanowire arrays with mesoporous structure enabled a large specific surface area and shortened the ion diffusion distance, and (iii) the freestanding electrodes effectively enhanced the charge transport and adhesion between the active materials and the conductive CF for the desired electrical conductivity and structural stability.

In addition to the single-metal oxides, spinel-structured bimetallic oxides AB_2_O_4_ (A and B are metals) have been shown with further improved application potential as an OER catalyst owing to the electron hopping between the different valence states of metals in the O sites.^220,221^ However, most of these studies on the AB_2_O_4_-based OER catalysts were concentrated on the powder or film forms, where their catalytic performance could be affected by the poor intrinsic conductivity and inferior structure stability arising from the agglomeration and collapse during the electrochemical reaction.^222^ With a large degree of structural adjustability/controllability, these MOF materials provide an excellent opportunity to construct freestanding AB_2_O_4_-based OER catalysts, although it is still a challenge to directly grow some of the bimetal-based MOF nanoarrays on conductive substrates. Recently, Li et al. developed a polyaniline (PANI) assistance strategy to prepare freestanding MOF-74-Co/Fe nanorod arrays (NRAs) on the surface of NF, where PANI film could facilitate the adsorption of metal ions (Co^2+^ and Fe^2+^) and orientated growth of MOF crystals.^63^ As illustrated in Figure 19d, 1D CoFe_2_O_4_/C NRAs with a porous structure were successfully synthesized by a simple calcination process under a N_2_ atmosphere, which can directly serve as a freestanding OER catalyst electrode. TEM studies (Figure 19e) showed that a CoFe_2_O_4_/C nanorod possessed the hierarchical porous structure, which was conductive to achieving remarkable catalytic performance due to the large specific surface area, fast ion diffusion, high accessibility and utilization of active sites, and free and fast diffusion of O_2_. Benefiting from these advantages, the NF@NC–CoFe_2_O_4_/C NRAs delivered a low overpotential (240 mV at 10 mA cm^–2^) and a small onset potential (∼1.45 V) (Figure 19f), which were even better than those of the noble metal oxides. Besides, compared to the NF@NC–CoFe_2_O_4_/C NRAs, the powder form of the NC–CoFe_2_O_4_/C electrode exhibited an exceedingly high overpotential (315 mV) and onset potential, demonstrating the superiority of the freestanding MOF design.

Moreover, PBAs are among the precursors to synthesize high-performance OER catalysts owning to their abundant adjustable combinations of metal elements, simple preparation process, and various morphological structures that can be made.^223^ In the early stage of investigations, most works focused on the catalytic electrodes in the powder forms. Thereafter, there was considerable effort in freestanding PBAs design. For example, Li et al. reported a template-assistance strategy to prepare freestanding KNi[Fe(CN)_6_] PBA nanocube arrays on Ni(OH)_2_ nanosheets grown on NF in which Ni(OH)_2_ nanosheets not only facilitated the in situ growth of PBAs but also improved the structural stability of the freestanding electrode.^216^ As shown in Figure 19g, oxygen-doped nickel–iron phosphide nanocube arrays (Ni_2_P/(NiFe)_2_P(O) NAs) with hollow structures were obtained by a phosphating treatment, which could improve the charge transport capability of active materials. The hierarchical nanosheet arrays with hollow structures were verified by the SEM and TEM results (Figure 19h), which were shown to greatly increase the specific surface area and enhance the accessibility of active sites. The catalytic property of the Ni_2_P/(NiFe)_2_P(O) NAs in 1 M KOH aqueous electrolyte was analyzed by the polarization curves (Figure 19i), delivering an ultralow overpotential of 260 mV as the current density reached 500 mA cm^–2^.

#### 5.1.2 Freestanding Pristine MOF Electrodes for the OER

**Figure 20:**
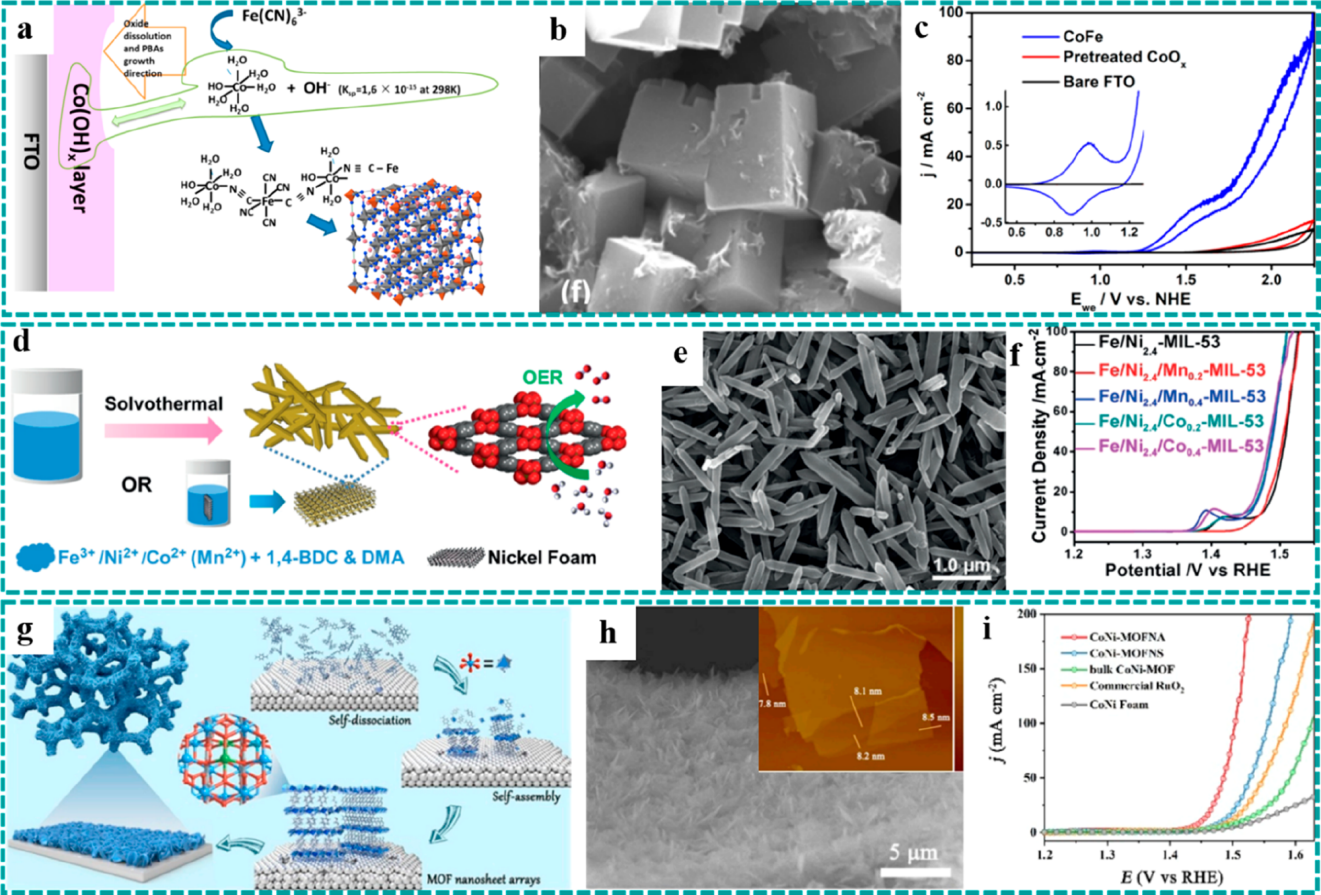
Freestanding pristine MOFs as electrodes for the OER. (a) Schematic diagram of the mechanism of preparing the freestanding CoFe electrode by the template-assisted method. (b) SEM image of CoFe. (c) LSV curves at 5 mV s^–1^ of CoFe, CoO_*x*_, and fluorine–tin–oxide-coated glass slides (FTO). Reproduced with permission from ref (64). Copyright 2016 American Chemical Society. (d) Preparation schematic and (e) SEM image of Fe/Ni/Co(Mn)-MIL-53. (f) LSV curves of Fe/Ni/Co(Mn)-MIL-53 and the control groups. Reproduced with permission from ref (74). Copyright 2018 Wiley-VCH. (g) Schematic showing the preparation process and (h) SEM and AFM images of CoNi–MOFNA. (i) LSV curves of CoNi–MOFNA and the control groups for the OER. Reproduced with permission from ref (210). Copyright 2020 Elsevier.

Compared with MOF derivatives, freestanding pristine MOFs simplify the process of electrode preparation and retain the inherent ordered and open framework structures, which help prevent the destruction of organic ligands and agglomeration of active sites.^64,74,83,210,224−227^ Therefore, developing feasible strategies to construct freestanding pristine MOFs and using them directly as catalysts are highly meaningful to facilitate the accessibility of active sites and improve the catalytic efficiency. PBAs are generally more stable than transition metal oxides/hydroxides in acidic and neutral conditions, exhibiting a wider pH application range. On the basis of the local dissolution approach of the transition metal oxides in neutral or slightly acidic solutions, Jose Ramon Galan-Mascaros et al. utilized CoO_*x*_ nanowires as a cobalt source to prepare freestanding cobalt hexacyanoferrate (CoFe) electrodes for the OER catalysts by a simple chemical bath method, overcoming the issue that PBA-based materials are challenging to nucleate and grow on substrates.^64^ As illustrated in Figure 20a, dissolved Co^2+^ immediately reacted with Fe(CN)_6_^3–^ to form CoFe cubes in situ on the surface of CoO_*x*_ nanowires (Figure 20b). The OER catalytic performance of the CoFe electrode was assessed in a neutral aqueous electrolyte. Compared with CoO_*x*_, the freestanding CoFe electrode adequately demonstrated its unique advantages, including a small overpotential of 334 mV (Figure 20c). This work stimulated broad enthusiasm to develop reasonable strategies to construct freestanding pristine MOFs as efficient catalysts for the OER.

An appropriate combination of diversified metal types in active materials can achieve enhanced catalytic performance because of the synergistic effect involved. There have been extensive efforts devoted to the design and preparation of freestanding polymetallic MOFs as high-efficiency catalysts for the OER. For example, Lang et al. reported a freestanding rod-like trimetallic MOF (Fe/Ni/Co(Mn)-MIL-53) grown on NF by a solvothermal method as a catalyst for the OER, as illustrated in Figure 20d.^74^ The introduction of other metal ions (e.g., Ni) in MIL-53(Fe) was attributed to the partial replacement of FeO_6_ by the NiO_6_ octahedrons, where the content ratios of Fe and Ni could be easily controlled. SEM studies (Figure 20e) showed that rod-like Fe/Ni_2.4_-MIL-53, with a length of ∼900 nm and a diameter of ∼100 nm, was uniformly distributed on the surface of NF. Figure 20f shows the OER polarization curves of Fe/Ni–MIL-53 and Fe/Ni/Co(Mn)-MIL-53, where Fe/Ni_2.4_/Co_0.4_-MIL-53 possessed a remarkable OER performance with a low overpotential of 219 mV at 10 mA cm^–2^ (236 mV at 20 mA cm^–2^). The excellent OER performance delivered by Fe/Ni_2.4_/Co_0.4_-MIL-53 was attributed to the following. (i) In Fe/Ni-based catalysts, where Ni was the active core for the OER, the Ni^3+^/Ni^2+^ peak would be shifted to a high potential when in combination with Fe. Fe/Mn(Co)-based MOFs delivered a poor OER performance.^221,228^ (ii) In comparison to the Fe/Ni-based MOFs, the introduction of Co shifted the Ni 2p spectrum to a higher binding energy by changing the coordination environment of the active centers, further enhancing the corresponding OER performance.^229^ Due to the synergy effect of the metal combination, efficient modulation in the electronic environment of the active metal sites was conductive to optimize the electrocatalytic performance for the OER. (iii) The NF played an essential role in improving the electrocatalytic performance, where the active materials anchored on the surface of porous NF would achieve fast charge transport and ion diffusion.

Although the above-mentioned freestanding pristine MOFs delivered good electrocatalytic performance for the OER, these MOFs possessed a 3D bulk structure in micrometer scales, which could result in relatively low electrical conductivity, slow mass transport, and inner inaccessible metal active sites. Recent studies have proven that the active materials with ultrathin 2D nanostructures are more conducive to enhance the catalytic performance than the micrometer-sized bulk materials owing to their richer active sites formed by the unsaturated coordinated metal atoms at the surface of 2D nanosheets.^23,230^ Therefore, there would be considerable new opportunities in developing feasible strategies to construct freestanding 2D pristine MOFs with improved structures and performance. Recently, Dong et al. developed a self-dissociation-assembly strategy to in situ grow ultrathin MOF nanosheet arrays on the surface of CoNi alloy foam, directly serving as freestanding catalysts (CoNi–MOFNA) for the OER.^210^ As illustrated in Figure 20g, the preparation process of the freestanding MOFs with a 2D nanosheet nanostructure could be divided into two steps. (i) A supply of metal sources and the formation of seed layers. Owing to the oxidation and dissolution process of CoNi foam under the reaction of benzenedicarboxylic acid (BDC), the Co^2+^ and Ni^2+^ released on the surface of CoNi foam could immediately react with the BDC molecules and nucleate to form a seed layer on CoNi foam. (ii) In situ self-assembly of 2D MOF nanosheets. The use of an appropriate seed layer, control of the intrinsic structural topologies of CoNi–MOF, and appropriate synthesis conditions facilitated the construction of freestanding 2D MOFs. As a result, uniform and ultrathin nanosheet arrays were tightly grown on the surface of CoNi foam (Figure 20h) in which the average thickness of the nanosheets was ∼8.2 nm according to the measurement result of atomic force microscopy (AFM) as shown in the inset of Figure 20h. To demonstrate the superiority of 2D MOF nanosheets, the LSV curves of CoNi–MOFNA and bulk CoNi–MOF electrodes at 5 mV s^–1^ in an alkaline electrolyte are shown in Figure 20i in which the current density of the CoNi–MOFNA electrode was sharply increased at a low onset potential (*E*_onset_) of 1.41 V. Moreover, compared with the overpotential (284 mV) of the bulk CoNi–MOF electrode, the CoNi–MOFNA electrode delivered a lower overpotential of 215 mV at 10 mA cm^–2^, implying the excellent catalytic performance of the freestanding pristine MOF with a 2D nanosheet structure for the OER.

### 5.2 Freestanding MOF Derivative Electrodes for the HER

**Figure 21:**
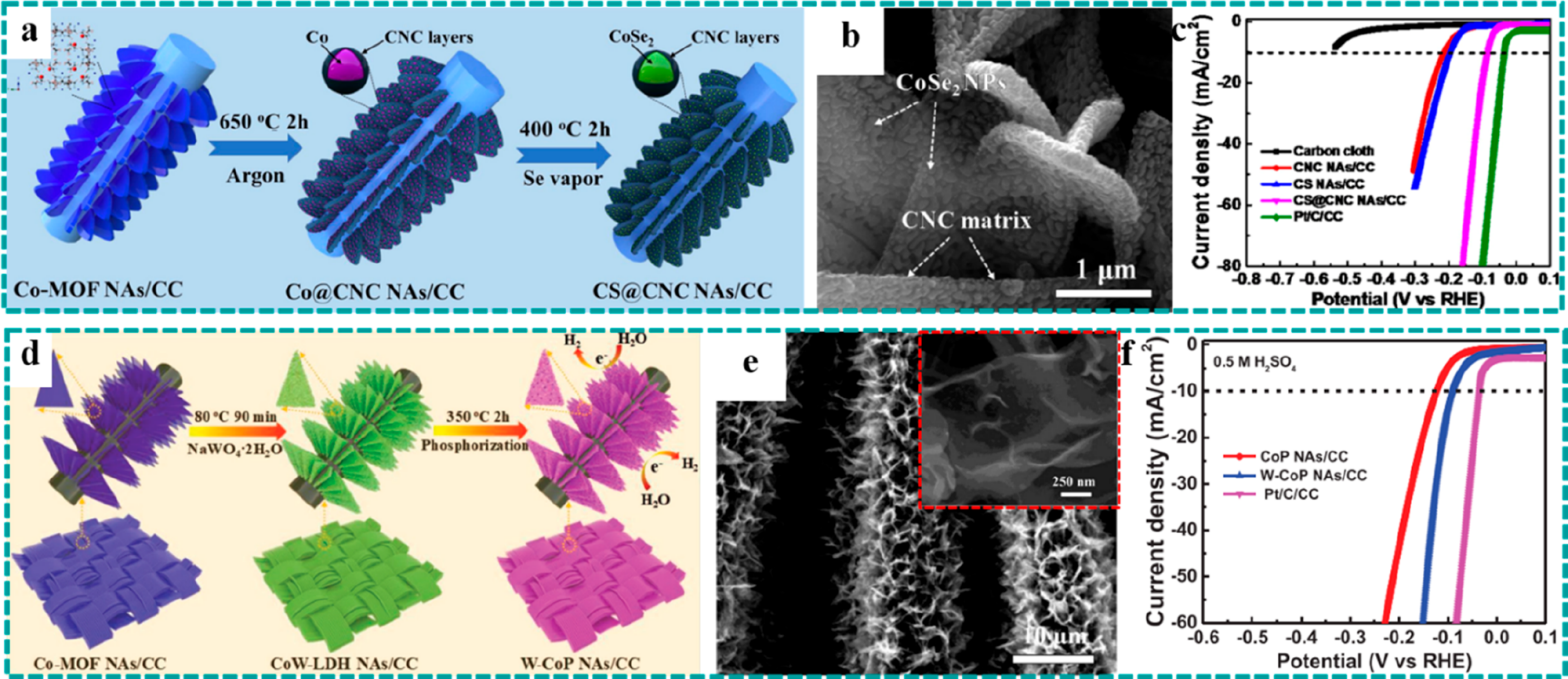
Freestanding MOF derivative electrodes for the HER. (a) Schematic illustration for the preparation, (b) SEM image of CS@CNC NAs/CC, and (c) LSV curves of CS@CNC NAs/CC and the control groups in 0.5 M H_2_SO_4_ electrolyte. Reproduced with permission from ref (76). Copyright 2019 Elsevier. (d) Schematic illustration of the synthesis procedure, (e) SEM images of W-CoP NAs/CC, and (f) LSV curves of W-CoP NAs/CC, CoP NAs/CC, and Pt/C/CC in 0.5 M H_2_SO_4_ electrolyte. Reproduced with permission from ref (112). Copyright 2019 Wiley-VCH.

As the cathodic half-reaction in water splitting, the HER has drawn ever-increasing attention since hydrogen, as its product, is a key component in developing sustainable energy.^231,232^ To date, platinum-based materials have been demonstrated as ideal electrocatalysts for the HER.^233,234^ However, the high cost and scarce resources have severely impeded their wide applications. Therefore, extensive efforts have been shifted to develop non-noble metal-based materials as highly efficient electrocatalysts because of their low cost, intrinsic catalytic properties, and stability, including transition metal oxides, nitrides, oxides, selenides, phosphides, etc., which are expected to replace the noble platinum-based electrocatalysts in large-scale applications.^235,236^ Nevertheless, the relatively low electrical conductivity and small specific surface area become the bottleneck of transition metal-based catalysts for the HER. Recently, constructing freestanding MOF derivative electrodes with 3D high-conductivity skeletons and porous structures has been acting as an effective strategy to solve the above issues for highly efficient HER catalysts.^76,112,211,237−239^ Indeed, several MOFs have been shown to provide abundant N element and form NC or metal-based active materials during the calcining processes, greatly enhancing the HER performance. For example, Chen et al. reported MOF-derived CoSe_2_ nanoparticle-embedded Co–N-doped carbon nanoflake arrays on the surface of CC as freestanding catalytic electrodes (CS@CNC NAs/CC) for the HER.^76^Figure 21a shows the schematic diagram of its synthesis process in which the derivative process could be divided into two steps: carbonization and selenization. After the high-temperature treatment, the 3D nanosheet arrays could be well preserved, and there were many small protrusions evenly distributed on their surface (Figure 21b), implying the uniform distribution of CoSe_2_ nanoparticles. In addition, as further demonstrated by the TEM studies, the CoSe_2_ nanoparticles were tightly encapsulated within amorphous carbon layers. The ingenious core–shell structure enhanced the charge transport for excellent conductivity and improved the structural stability for long-term cycle life. More importantly, the porous hybrid structure of CS@CNC NAs/CC was more conductive to capture the hydrated proton to form the intermediate H_ads_. To measure the corresponding electrocatalytic performance for the HER, Figure 21c exhibits the LSV curve of CS@CNC NAs/CC in 0.5 M H_2_SO_4_ electrolyte. Impressively, CS@CNC NAs/CC delivered remarkable HER performance with a low overpotential of 84 mV at 10 mA cm^–2^, close to the performance of the Pt-based electrode (35 mV). The CS@CNC NAs/CC electrode also could keep the low overpotential of 84 mV for 72 h, revealing superior structural stability.

Heteroatom doping has been proven to be a valid approach to improve the catalytic performance of transition metal-based materials through the modulation of electronic structures and charge conductivity.^240−242^ As a common strategy to obtain MOF derivatives, ion exchange provides an opportunity to achieve the heteroatom doping of transition metal-based materials as freestanding electrodes for enhanced HER performance. For example, in one of our previous works, we developed a liquid-phase reaction and a subsequent phosphorization process to synthesize W-doped CoP nanoflake arrays with the hierarchical porous structures on CC (Figure 21d) serving as the freestanding catalytic electrode (W–CoP NAs/CC) for the HER.^112^ As shown in Figure 21e, the open nanosheet arrays with high porosity shortened the ion diffusion distance, exposed abundant active sites, and facilitated the fast diffusion of H_2_ bubbles for high catalytic efficiency. Apart from the advantages of freestanding design and nanosheet arrays, the W doping could effectively modulate the electronic environment of the active sites, thereby improving the HER catalytic performance. On the basis of the elaborate structural design and ion doping, W-CoP NAs/CC delivered a low overpotential (10 mA cm^–2^) of 89 mV in 0.5 M H_2_SO_4_ electrolyte (pH ≈ 0) (Figure 21f), much lower than that of CoP NAs/CC without W doping (123 mV), implying the effectiveness of W doping for improving the catalytic performance. More importantly, the excellent HER performance of W-CoP NAs/CC was exhibited at wide pH values in which low overpotentials of 94 and 102 mV were obtained in alkaline (pH ≈ 14) and neutral (pH ≈ 7) electrolytes, respectively, indicating the vast application range of W-CoP NAs/CC catalysts.

### 5.3 Freestanding MOF-Based/Derived Electrodes for Overall Water Splitting

#### 5.3.1 Freestanding MOF Derivative Electrodes for Overall Water Splitting

**Figure 22:**
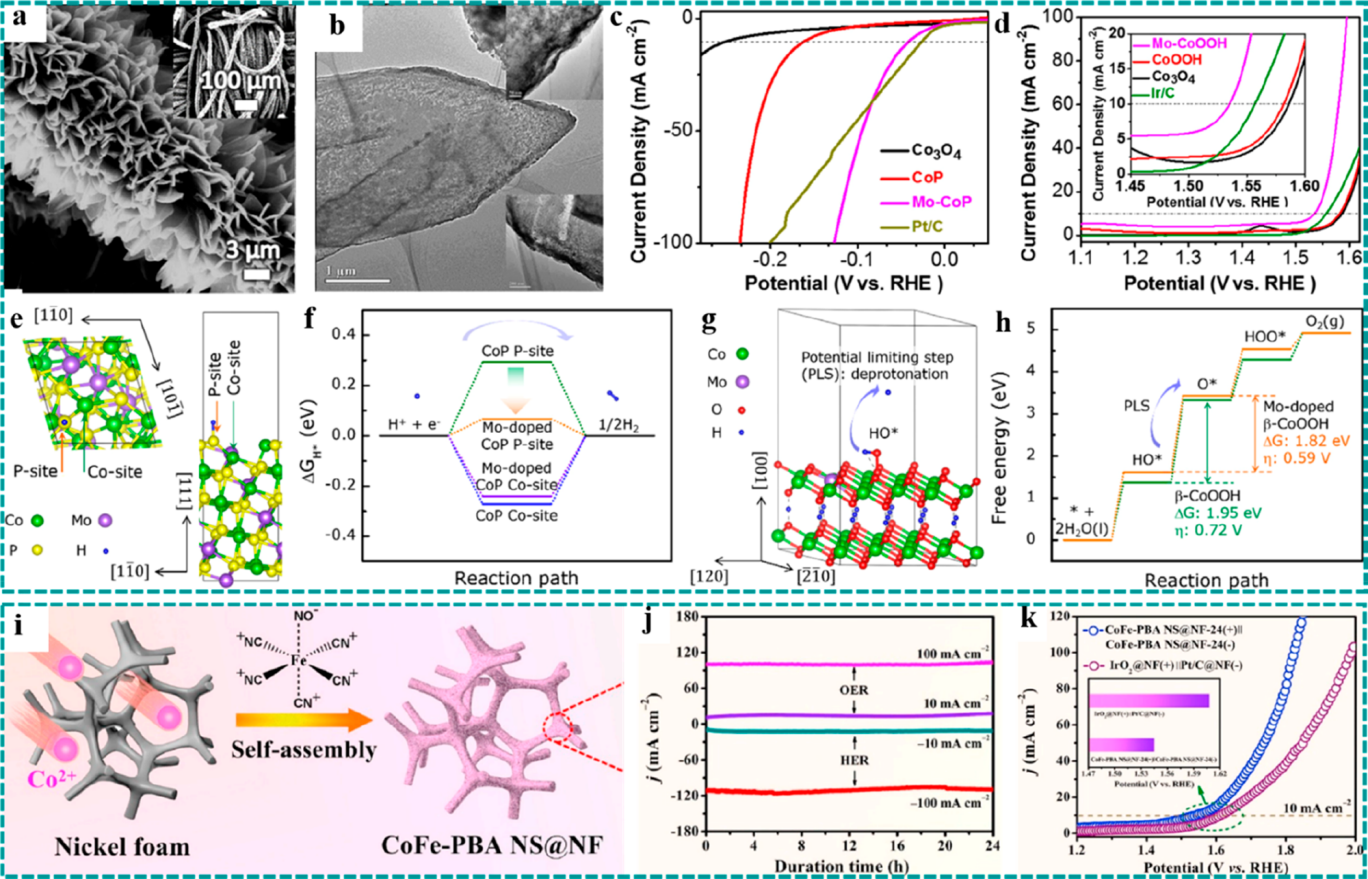
Freestanding MOF-based/derived electrodes for overall water splitting. (a) SEM and (b) TEM images of Mo–CoP nanoarrays. (c) LSV curves for the HER of Mo–CoP and the control groups. (d) LSV curves for the OER of Mo–CoOOH and the control groups. (e) Atom structure model of the (111) surface of Mo–CoP. (f) HER free energy of P and Co sites for CoP and Mo–CoP. (g) Atomic structure model of the (111) surface of Mo–CoOOH. (h) Standard free energy of the OER path for CoOOH and Mo–CoOOH. Reproduced with permission from ref (113). Copyright 2018 Elsevier. (i) Preparation schematic of CoFe–PBA NS@NF. (j) Catalytic stability tests of CoFe–PBA NS@NF for the HER and OER. (k) LSV curves of CoFe–PBA NS@NF//CoFe–PBA NS@NF and Pt/C@NF//IrO_2_@NF systems for overall water splitting. Reproduced with permission from ref (209). Copyright 2020 Elsevier.

As described above, extensive efforts have been devoted to designing and preparing freestanding MOF-based/derived electrodes as catalysts for the OER or HER, exhibiting excellent catalytic performance. Nevertheless, overall water splitting consists of the OER and HER, which would require active materials to achieve highly efficient catalytic properties for both the OER and the HER. Therefore, constructing freestanding MOF-based/derived electrodes with reasonable morphological structures and chemical compositions as bifunctional catalysts is of great interest, which could simultaneously reduce the overpotentials of both the HER and the OER for highly efficient overall water splitting.^69,77,82,113,116,209,214,243−245^ For example, Wang et al. reported MOF-derived hollow Mo-doped CoP (Mo–CoP) nanowall arrays grown on CC, directly serving as binder-free bifunctional electrocatalysis for overall water splitting.^113^ After a hydrolysis reaction and subsequent phosphidation process, the Co–MOF nanowall arrays with a compact structure were transformed to hollow Mo–CoP nanoarrays with porous structure, uniformly covering the carbon fibers of CC, as shown in Figure 22a and 22b. To explore the HER and OER performance of Mo–CoP, the LSV curves were tested at a three-electrode system in 1 M KOH. For the HER, compared to those of hollow CoP (160 mV) and Co_3_O_4_ (242 mV) nanoarrays, the Mo–CoP electrode delivered a much lower overpotential (*I* = 10 mA cm^–2^) of 40 mV (Figure 22c), implying that phosphidation and Mo doping could greatly improve the HER performance of the electrocatalyst. For the OER, the Mo–CoP would be in situ converted into Mo–CoOOH with the original hollow nanowall arrays owing to the continuous oxidation process. As observed in Figure 22d, Mo–CoOOH exhibited a small overpotential (*I* = 10 mA cm^–2^) of 305 mV, which was lower than those of CoP-derived CoOOH (323 mV) and Co_3_O_4_ (356 mV), further demonstrating the effectiveness of Mo doping for the catalytic reaction. On the basis of the remarkable HER and OER performance of Mo–CoP and its derivative, the corresponding property for overall water splitting was expected. The system assembled by Mo–CoP and Mo–CoOOH exhibits a small operating voltage of 1.56 V, even better than the performance delivered by the Pt/C(−)//Ir/C(+) system, indicating superior catalytic efficiency for overall water splitting. To understand the enhancement mechanism involved for the HER and OER, density functional theory was adopted to investigate the effect of Mo doping (Figure 22e). For the HER, the (111) surface of Mo–CoP was chosen as the analytical model in which the ratio of Mo:Co was 5:13, corresponding to the EDS result. A free energy change Δ*G*_H*_ of hydrogen adsorption was used as the standard to evaluate the performance of the catalysts. As shown in Figure 22f, the Δ*G*_H*_ values of the P and Co sites were 0.29 and −0.27 eV, respectively, while the introduction of Mo resulted in the sharp decline of the P site to 0.07 eV, demonstrating that Mo doping played a vital role in the improvement of the HER performance. Figure 22g shows the calculated model of Mo–CoOOH in which the potential limiting step (PLS) has the highest free energy change Δ*G*° in four elementary steps. The values of Δ*G*2° and the calculated overpotential η of Mo–CoOOH are 1.82 eV and 0.59 V, respectively, which was lower than the overpotential (0.72 V) of β-CoOOH (Figure 22h), proving the reason for Mo doping for enhanced OER performance. Through the above analysis, the outstanding catalytic performance could be attributed to the following: (i) introduction of Mo lowered the energy barrier, (ii) ordered nanoarrays with a hollow porous structure offered rich reactive sites and rapid ion transport, and (iii) the design of the freestanding electrode and phosphorization enhanced the electrical conductivity.

#### 5.3.2 Freestanding Pristine MOF Electrodes for Overall Water Splitting

In comparison with MOF derivatives, direct application of pristine MOFs as catalysts for overall water splitting could make better use of the ordered channel structure and large specific surface area as well as accessible active sites. However, the generally poor electrical conductivity of bulk MOFs is a limiting parameter in their development of use in water splitting.^246^ Indeed, few pristine MOFs could deliver satisfying HER performance, including PBAs, which is a large barrier in developing the pristine MOF into bifunctional catalysts. Recently, in attempts to solve some of the above issues, Wu et al. developed a simple chemical bath method to synthesize ultrathin Co[Fe(CN)_5_(NO)]·4H_2_O nanosheet arrays on NF, serving as a freestanding bifunctional catalyst (CoFe–PBA NS@NF) for overall water splitting, as illustrated in Figure 22i.^209^ For catalytic reactions, the existence of unsaturated metal centers is usually beneficial to the improvement of catalytic activities.^77,247^ Besides, construction of freestanding electrodes has proven to be an effective strategy to prevent ultrathin nanosheets from aggregating, ensuring that the CoFe–PBA NS@NF always maintains a large accessible active area during the catalytic reaction process. Moreover, the largely seamless contact between nanosheet arrays and NF overcame the limitation of the intrinsic poor electrical conductivity of PBA-based materials and effectively suppressed the peeling off of active materials during the generation process of bubbles. Benefiting from the above advantages, CoFe–PBA NS@NF achieved low overpotentials (10 mA cm^–2^) of 48 and 256 mV for the HER and OER in an alkaline electrolyte (Figure 22j), respectively, and a negligible activity decay occurred for 24 h, exhibiting excellent catalytic stability. The CoFe–PBA NS@NF//CoFe–PBA NS@NF system only needed a small voltage of 1.545 V to drive the electrolysis of water at 10 mA cm^–2^, which was even lower than that (1.608 V) of the Pt/C@NF//IrO_2_@NF system (Figure 22k).

## 6 Conclusions and Perspectives

In summary, we have thoroughly examined the recent progress in the design, preparation, and electrochemical applications of freestanding MOF-based/derived electrodes, where the designs of synthetic strategies and the advantages for constructing high-performance electrodes for EESC are particularly highlighted. Compared to the traditional powder-formed electrodes obtained by slurry-casting methods, freestanding electrodes skip the use of the undesired binders and offer a seamless contact between the active materials and the conductive substrates, improving the electron transport and mechanical stability, which are conductive to overcome the limitations caused by the generally poor electrical conductivity of MOF-based electrode materials, especially for most of the pristine MOFs. In combination with a large specific surface area, highly tunable level of porosity, and abundant active sites, freestanding MOF-based/derived electrodes exhibit several unique advantages when employed as electrodes in energy storage and conversion, as summarized by the examples in Table S7, including Li-based batteries, SIBs, Zn-based batteries, NFBs, supercapacitors, and water splitting.

**Figure 23:**
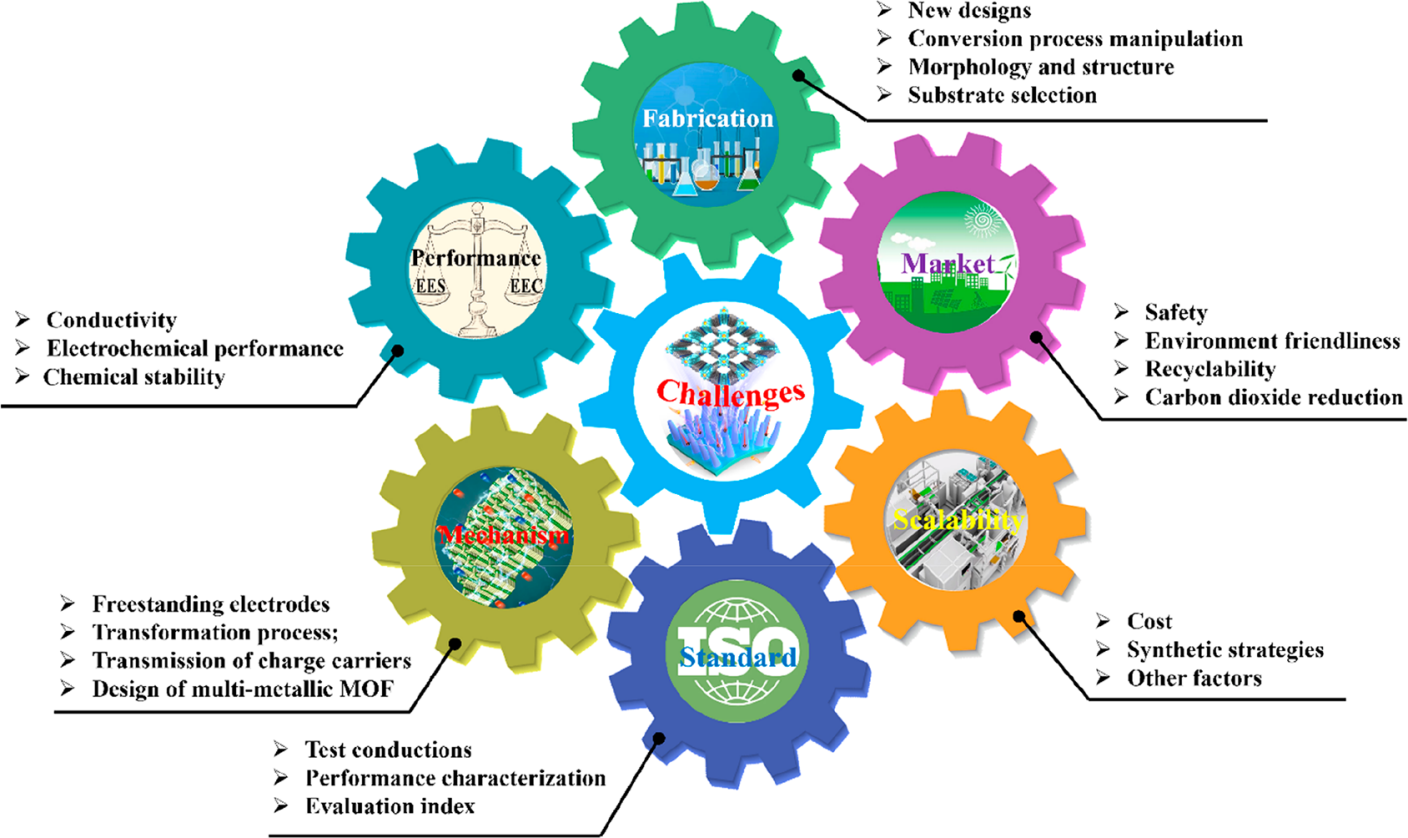
Challenges of freestanding MOF-based/derived electrodes for EESC.

Although great achievements have been made in developing various freestanding MOF-based/derived electrodes in the past decade, it still requires a lot of effort to address some of the existing challenges for large scale practical applications in the near future. Herein, the existing difficulties and challenges in developing freestanding MOF electrodes are emphatically analyzed and discussed from the perspectives of fabrication, performance, mechanism, multifunction, standard, and scalability. As shown in Figure 23, each factor can include several aspects that restrict and promote each other.

### 6.1 Precise Fabrication

(I) Design and synthesize freestanding MOFs of the targeted structures and performance. There is a rich variety of freestanding pristine MOFs that can be made for applications in EESC by proper design and fabrication. Although many excellent works on freestanding pristine MOFs have been reported, only a small fraction has been explored in the vast pool of >20 000 MOFs reported. Therefore, it would be highly desirable to construct a diversified range of freestanding MOF-based electrodes in order to enrich the urgently needed choices for applications and also the fundamental knowledge on the MOFs themselves and their interfacial interactions with substrates. In addition to the MOFs themselves, the surface functionalities of the substrates have proven to have considerable significance in the control of the nucleation, morphology, and performance of freestanding pristine MOFs.
(II) Explore and optimize the transformation process from pristine MOFs to their derivatives. As detailed in this review, freestanding MOF derivatives possess generally porous nanostructures and improved electrical conductivity, exhibiting good electrochemical performance in EESC. However, it is still challenging to achieve an accurate control of the delicate structures of MOF derivatives due to the limited understanding on the transformation process at high temperatures. The rapid development of in situ technology will help researchers uncover the largely unsolved mechanisms of the transformation process in the future, further preciously tuning the structures of MOF derivatives.
(III) Regulate and control the morphology and structure of freestanding pristine MOFs. Compared to other electrode materials, as discussed in this review, MOFs possess numerous advantages, including a regular pore/channel structure, a large specific surface area, and tunable combinations of metal and organic lingers. Nevertheless, the general lack of knowledge on the self-assembly of pristine MOFs in a closed reaction system makes it difficult to effectively regulate the pore size and nanostructure morphology, unable to make the best use of their advantages. An in-depth understanding of the growth mechanism and design would endow the freestanding MOFs with more and better functions in the future.
(IV) Select appropriate conductive substrates. As a typical class of freestanding electrodes, the conductive substrates facilitate the fast transport of electrons, achieve a uniform dispersion of MOF-based active materials, and restrain their agglomeration during the electrochemical process. To date, conductive substrates are mainly divided into two categories: metal-based and carbon-based substrates, each of which has its unique features, pros, and cons. The former offers excellent electrical conductivity but suffers from a heavy mass and small surface area, leading to a relatively low mass loading of active materials. On the contrary, the latter exhibits the advantages of being lightweight and having a large surface area but is generally limited by the relatively poorer conductivity, especially for batch preparation. Thus, development of an appreciate conductive substrate with the combined advantages will be one of the focuses of constructing high-performance freestanding electrodes.

### 6.2 Performance Improvement

(I) Electrical conductivity of pristine MOFs. Compared to their conducting derivatives, most pristine MOFs with intact channel structures and active sites suffer from an intrinsically poor electrical conductivity. One of common approaches to improve the electrical conductivity of MOF-based electrodes is the construction of freestanding architectures, as discussed in this review. On the basis of the recent progress made with the conductivity of MOFs, some of the effective strategies to enhance the intrinsic conductivity of MOFs include the following. (a) In the structure approach, rational orbital interactions have been shown to be able to enhance the electrical conductivity of pristine MOFs. Two examples are the orbital overlaps between metal ions and organic linkers and efficient ligand–ligand π···π stacking, where theoretical simulations have played an important role in guiding the design and preparation of conductive MOFs with enhanced conductivity. (b) In compositions, introduction of appropriate guest molecules in the pore channels of frameworks can be conductive to improving the conductivity of nonconductive MOFs, such as by redox-active molecules, conductive polymers, etc., which not only preserve the intrinsic ordered channel structures but also endow the target MOFs with unique functionalities. (c) In morphology, compared to MOFs in bulk forms, MOF nanoflakes or nanowires grown on conductive substrates can directly serve as electrodes for EESC, and several of them have shown satisfactory electrochemical performance, although the overall conductivity is not perfectly high. This has been attributed to the unsaturated metal atoms in coordination at the surface of 2D nanoflakes and 1D nanowires, which can expose more active sites and enhance the overall electrical conductivity. Besides, these MOF nanostructures could shorten the electron/charge transport pathway and accordingly improve the conductivity. An improvement in conductivity of MOFs and MOF-based electrodes would help achieve both a higher utilization efficiency of the active sites and a higher electron transfer rate, which would be conductive to delivering high power and energy densities as well as catalytic activity.
(II) Relationship between electrochemical properties and structure designs of freestanding MOF-based/derived electrodes. Although freestanding conductive MOFs show many promising prospects in EESC, their electrochemical properties are not satisfied in many cases. Therefore, understanding the structure–function relationship should be taken into account. For example, for applications in supercapacitors, the design of freestanding MOF-based/derived electrodes should prioritize the large specific surface area and good conductivity, while for metal-ion batteries organic linkers and metal nodes with abundant active sites in freestanding MOF-based/derived electrodes should be preferred for the targeted high energy densities. Moreover, for catalytic reactions, organic linkers and metal nodes providing elements with high catalytic activities are required and designed for effective catalytic performance. These structure–function relationships then provide valuable guidance for fabricating freestanding MOF-based/derived electrodes with better performance in EESC. Machine learning-aided simulations together with the materials database of existing freestanding MOFs can be used to predict their electrochemical performance and applications and further assist in designing new freestanding MOFs. In turn, the electrochemical performance by the thus-designed freestanding MOF-based electrodes would guide the theoretical calculation, thereby constructing a closed-loop active-learning environment. Therefore, machine-learning-aided simulation as a powerful tool is expected to help establish the relationships between the freestanding MOF materials and their electrochemical performance. This enables one to wisely choose freestanding MOFs for targeted applications. In this connection, Table S8 summarizes the current criteria for selecting freestanding MOF properties for different electrochemical applications, hoping to help readers choose the correct freestanding MOF for a specific application.
(III) In comparison to carbon-based and metal oxide-based electrode materials, freestanding pristine MOF electrodes generally exhibit poor stability in alkaline/acidic aqueous electrolytes, even in some neutral aqueous electrolytes. To date, construction of freestanding MOF-derived electrodes has effectively enhanced their chemical stability in an aqueous solution at the expense of an ordered pore structure and partial active sites. Therefore, it would be of value to investigate innovative strategies to improve the chemical stability of pristine MOFs for a broader range of practical applications. On the basis of theoretical calculations, proper combinations in choices of organic ligands and metal nodes would be among the strategies.

### 6.3 In-Depth Understanding of the Mechanism

Many underlying mechanisms have yet to be revealed and understood, limited by the present characterization technologies. With the rapid development of in situ characterization (XRD, X-ray photoelectron spectroscopy, TEM, SEM, and FTIR) combined with theoretical calculations, more efforts should be invested to gradually reveal the in-depth mechanism of freestanding MOF-based/derived electrodes from synthesis to application to provide more theoretical guidance for delivering better electrochemical performance. For example, in situ characterization would be a powerful tool to investigate the nucleation and growth of MOFs on the surface of substrates, which can help researchers optimize their morphologies and structures by purposefully controlling the reaction conditions. Similarly, a thorough understanding of the transformation processes to prepare MOF derivatives would be beneficial to achieving precise control of their delicate structures. In addition, theoretical calculations would be particularly useful to clarify the charge transport mechanism between the guest molecules and the MOF-based/derived hosts, providing a clear understanding of the mechanism of MOF-based/derived electrodes. Moreover, under the guidance of theoretical simulation, it can save the experimental “trials and errors” in designing and optimizing freestanding multimetallic MOFs for better electrochemical performance.

### 6.4 Evaluation Standard

In the initial development of freestanding MOF-based/derived electrodes for EESC, there is generally a lack of unified evaluation standards for test conditions, characterization, and performance evaluation. First, testing the experimental conditions, such as temperature, environment, and exact content of electrolytes, would significantly affect the properties and stabilities of freestanding MOF-based electrodes in EESC. Besides, the performance data characterized by the unified standard is difficult to compare. For example, performance characterization based on the surface area is common and important in EESC, including areal energy/power densities, overpotential (10 mA cm^–2^), cyclic stabilities, etc. However, the macroscopic unit area of many substrates (e.g., carbon paper vs carbon cloth, metal foam with different meshes) can be very different from the actual surface area, causing a lack of credibility in performance comparisons. Finally, the unified evaluation indexes of freestanding MOF-based electrodes are quite difficult or almost impossible due to numerous influencing factors. Furthermore, other factors such as preparation cost, mass loading, binding force, mechanism flexibility, electrochemical stability, multifunction, etc., would also affect the evaluation of freestanding MOF-based/derived electrodes. Therefore, establishing the corresponding scientific research systems and standardized evaluation indexes in the near future would be desirable, which can accelerate the synthetic design, performance optimization, and batch preparation of freestanding MOF-based/derived electrodes.

### 6.5 Industrial Scalability

Although great progress has been achieved in laboratories, development of freestanding MOF-based/derived electrodes is still in its infancy, and almost none of them could be prepared on a large scale in a cost-effective way. In order to realize the transition from lab devices to industry products, several challenges of batch preparation of freestanding MOF-based/derived electrodes need to be addressed. (I) Cost. Most organic ligands and reaction solvents are expensive, severely limiting the large-scale preparation of freestanding MOF-based/derived electrodes. Thus, designing low-cost freestanding MOFs would be a key step to achieve the above target. (II) Synthetic strategy. Besides cost, scale-up preparation of freestanding MOF-based/derived electrodes also requires the universality and operability of synthetic strategies, avoiding harsh reaction conditions and the use of special equipment. Compared to thermal transformation and solvothermal/hydrothermal methods that need high temperature or pressure, the conditions required for chemical baths, hybridization, and electrodeposition are generally milder and more operational, thereby being more promising to develop strategies for the scale-up preparation of freestanding MOF-based/derived electrodes. (III) Other factors. From adequate preparation in the laboratory to large-scale preparation in industry, the physical and chemical properties of freestanding MOF-based/derived electrodes would also change significantly. For example, can the mass loading and uniformity of MOFs on the surface of substrates in scale-up preparation be consistent with the samples obtained in the laboratory? It is related directly to the electrochemical properties (energy densities or catalytic efficiency) of the devices. As another example, electrical conductivities would become a limiting factor of scale-up preparation of many freestanding MOF-based/derived electrodes using carbon-based substrates owing to the relatively poor conductivity of carbon-based materials, such as the conductive networks based on electrospinning, 3D printing, subsequent heat treatment, CNTFs, etc. Seeking reasonable strategies to improve the electrical conductivity of carbon-based substrates would be significant for overcoming the above limitation.

### 6.6 Potential Market

After addressing the above challenges, the next critical step of the commercial application of freestanding MOF-based/derived electrodes is oriented to market demand. With peak carbon dioxide emissions and a carbon neutrality plan on the agenda, the exploration of new clean energy and its matching storage systems will become the focus in the future. Therefore, the construction of EESC devices based on freestanding MOF-based/derived electrodes should consider the following factors. First, as an energy storage device, in addition to good electrochemical performance, the device should ensure high safety, environmental friendliness, and recyclability. Next, as an overall water-splitting system, a device with high catalytic efficiency, low cost, and neutral electrolytes should be considered as a priority. Besides, it is worth mentioning that carbon dioxide reduction, as an important strategy in carbon neutrality, has attracted much attention. Due to the high porosity, unique structural features, and large surface area, MOF-based/derived materials have been regarded as reliable candidates for the adsorption, separation, and electroreduction of carbon dioxide. Combined with the advantages of unique architectures, freestanding MOF-based/derived electrodes could become some of the most promising catalysts for carbon dioxide reduction in the near future.

Indeed, opportunities and challenges coexist. Theoretical exploration guides the synthesized design, the synthesized design regulates the structural composition, the structural composition determines the electrochemical property, and the electrochemical property feeds the theoretical exploration. With the mutual promotion of synthesized design, structural regulation, and theoretical exploration, there will be booming development of freestanding MOF-based/derived electrodes for practical applications in EESC.

## Supporting Information Available

Freestanding MOF-based/derived electrodes for Li-based batteries, SIBs, ZABs, ZN/CBs, ZIBs, supercapacitors, the HER, and the OER; comparisons between freestanding electrodes and traditional powder-form electrodes; electrochemical applications and corresponding properties required (PDF)

## Abbreviations

MOFs: metal–organic frameworks
EESC: energy storage and conversion
PCPs: porous coordination polymers
CNTF: carbon nanotube fibers
CC: carbon clothes
NF/CF: nickel/copper foams
CFP: carbon fiber paper
ZIF: zeolitic imidazolate framework
SEM: scanning electron microscopy
KZHCF: K_2_Zn_3_(Fe(CN)_6_)_2_·9H_2_O
SIBs: sodium-ion batteries
PAN: polyacrylonitrile
rGO: reduced graphene oxide
CHNs: copper hydroxide nanostrands
H_3_BTC: 1,3,5-benzenetricarboxylic acid
TCPP: 5,10,15,20-tetrakis(4-carboxyphenyl)porphyrin
LIBs: lithium-ion batteries
NC: nitrogen-doped carbon
LDH: layered double hydroxide
TEM: transmission electron microscope
LABs: Li–air batteries
LSBs: Li–S batteries
ZIBs: Zn-ion batteries
ZABs: Zn–air batteries
CFC: carbon fiber cloth
CIMC: hierarchical monolithic 3D carbon networks
ISCF: interpenetrated and self-standing conductive framework
ORR: oxygen reduction reaction
OER: oxygen evolution reaction
mp-CNSs: mesoporous carbon nanosheets
PBAs: Prussian blue and its analogues
KNHCF: KNiFe(CN)_6_
QSS: quasi-solid state
ZN/CBs: Zn–Ni/Co batteries
CNW: carbon fibers networks
GO: graphene oxide
HER: hydrogen evolution reaction
NiCo-DH: nickel–cobalt double hydroxides
TDOS: total density of state
NFBs: Ni–Fe batteries
EDLC: electric double-layer capacitor
GCD curves: galvanostatic charge–discharge curves
NWAs: nanowire arrays
HHTP: 2,3,6,7,10,11-hexahydroxytriphenylene
MSCs: microsupercapacitors
PI: polyimide
LSG: laser-scribed graphene
BDC: benzenedicarboxylic acid
AFM: atomic force microscopy
*E*_onset_: low onset potential
FTO: fluorine–tin–oxide-coated glass
LSV: linear sweep voltammetry
PANI: polyaniline
NRAs: nanorod arrays
ESR: electron spin resonance
