# Supplementary material for: Freestanding Metal–Organic Frameworks and Their Derivatives: An Emerging Platform for Electrochemical Energy Storage and Conversion
Source: Chem Rev. 2022 Apr 21;122(11):10087–125. doi: 10.1021/acs.chemrev.1c00978 (PMC9185689; doi:10.1021/acs.chemrev.1c00978)
Supplement: Supplementary file 1 — cr1c00978_si_001.pdf [file cr1c00978_si_001.pdf]

Supporting Information

Freestanding Metal-Organic Frameworks and Their Derivatives: An Emerging Platform for Electrochemical Energy Storage and Conversion

Bing He[a+], Qichong Zhang[b,e*], Zhenghui Pan[c], Lei Li[d], Chaowei Li[h], Ying Ling[b], Zhixun Wang[a], Mengxiao Chen[g], Zhe Wang[a], Yagang Yao[f], Qingwen Li[b], Litao Sun[d*], John Wang[c,i*] and Lei Wei[a*]

a. School of Electrical and Electronic Engineering, Nanyang Technological University, 50 Nanyang Avenue, 639798, Singapore

b. Key Laboratory of Multifunctional Nanomaterials and Smart Systems, Suzhou Institute of Nano-Tech and Nano-Bionics, Chinese Academy of Sciences, Suzhou 215123, China

c. Department of Materials Science and Engineering, National University of Singapore, 117574 Singapore

d. SEU-FEI Nano-Pico Center, Key Laboratory of MEMS of Ministry of Education, Southeast University, Nanjing, 210096, China

e. Division of Nanomaterials and Jiangxi Key Lab of Carbonene Materials, Jiangxi Institute of Nanotechnology, Nanchang 330200, China

f. College of Engineering and Applied Sciences, and Collaborative Innovation Center of Advanced Microstructures, Nanjing University, Nanjing 210093, China

g. College of Biomedical Engineering and Instrument Science, Zhejiang University, Hangzhou, 310027, China

h. Henan Key Laboratory of New Optoelectronic Functional Materials, College of Chemistry and Chemical Engineering, Anyang Normal University, 436 Xian'ge Road, Anyang 455000, China

i. Institute of Materials Research and Engineering, A*Star, Singapore 138634, Singapore

[*] E-mail: qczhang2016@sinano.ac.cn; slt@seu.edu.cn; msewangj@nus.edu.sg; wei.lei@ntu.edu.sg

Table S1. Freestanding MOFs-based/-derived electrodes for Li-based batteries.

MOF	Sample	Substrate	Preparation strategy	Application	Electrolyte	Capacity	Rate performance	CR/CN	Ref.
ZIF-8	ZnO	No	Electrospinning + annealing	Anode for LIBs	1 M $LiPF_6$ OE	896 mAh g^{-1} at 0.1 A g^{-1}	492 mAh g^{-1} at 1.5 A g^{-1}	91%/100	[1]
Co-MOF	Co-based carbon hybrids	No	assembly +CVD	Anode for LIBs	1 M $LiPF_6$ OE	763 mAh g^{-1} at 0.1 A g^{-1}	212 mAh g^{-1} at 10 A g^{-1}	92%/550	[2]
ZIF-67	$LiCoO_2$	Cu foam	Chemical deposition +annealing	Cathode for LIBs	1 M $LiPF_6$ OE	155.4 mAh g^{-1} at 0.2 C	111.7 mAh g^{-1} at 15 C	78.4%/600	[3]
Co-MOF	Co_3O_4	NF	Chemical deposition +annealing	Anode for LIBs	1 M $LiPF_6$ OE	1226 mAh g^{-1} at 1 A g^{-1}	543 mAh g^{-1} at 20 A g^{-1}	68%/2000	[4]
ZIF-8	ZnO@NC	CNTs	Chemical deposition +annealing	Anode for LIBs	1 M $LiPF_6$ OE	950 mAh g^{-1} at 0.05 A g^{-1}	400 mAh g^{-1} at 1.0 A g^{-1}		[5]
Co-MOF	CuV_2O_6-$Co_3V_2O_8$	Cu foam	Chemical deposition+ ion exchange +annealing	Anode for LIBs	1 M $LiPF_6$ OE	1081.9 mAh g^{-1} at 0.25 A g^{-1}	306.5 mAh g^{-1} at 1.75 A g^{-1}	~100%/150	[6]
Zn/Co-MOF	$ZnCo_2O_4$	CC	Chemical deposition +annealing	Anode for LIBs	1 M $LiPF_6$ OE	2.61 mAh cm^{-2} at 0.12 mA cm^{-2}	2.05 mAh cm^{-2} at 1.2 mA cm^{-2}	~100%/100	[7]
ZIF-67	Co_3S_4	No	Electrospinning + annealing	Anode for LIBs	1 M $LiPF_6$ OE	756 mAh g^{-1} at 0.2 A g^{-1}	592 mAh g^{-1} at 1.0 A g^{-1}	~100%/200	[8]
Cu-BTC	CuO	3D graphene network	Chemical deposition +annealing	Anode for LIBs	1 M $LiPF_6$ OE	395 mAh g^{-1} at 0.1 A g^{-1}	219 mAh g^{-1} at 1.6 A g^{-1}	~99%/50	[9]
Cu-TCNQ	CuO	Cu foil	Chemical deposition +annealing	Anode for LIBs	1 M $LiPF_6$ OE	730 mAh g^{-1} at 0.1 A g^{-1}	367 mAh g^{-1} at 1.0 A g^{-1}	~92%/150	[10]
ZIF-67	carbon necklace papers	No	Electrospinning + annealing	Anode for LIBs	1 M $LiPF_6$ OE	940 mAh g^{-1} at 0.1 A g^{-1}	308 mAh g^{-1} at 2.0 A g^{-1}	~100%/400	[11]
Co-MOF	Co_3O_4	Ti foam plates	Electrodeposition+ annealing	Anode for LIBs	1 M $LiPF_6$ OE	700 mAh g^{-1} at 1.0 A g^{-1}	180 mAh g^{-1} at 50 A g^{-1}	~100%/2000	[12]
CPO-27	CPO-27	NF	Solvothermal method	Anode for LIBs	1 M $LiPF_6$ OE	670 mAh g^{-1} at 0.1 A g^{-1}	440 mAh g^{-1} at 2.0 A g^{-1}	93%/500	[13]
ZIF-67	Co/N-PCN@rGO	rGO	Chemical bath deposition +annealing	Cathode for Li-S batteries	1 M LiTFSI + 2.0 wt% $LiNO_3$ OE	1170 mAh g^{-1} at 0.2 C	880 mAh g^{-1} at 2 C	~67%/500	[14]
HKUST-1	porous carbon polyhedron	No	Filtration + chemical bath deposition +annealing	Cathode for Li-S batteries	1 M LiTFSI + 1.5 wt% $LiNO_3$ OE	1000 mAh g^{-1} at 0.2 C	650 mAh g^{-1} at 10 C	97.3%/500	[15]
ZIF-67	ZIF-67	3D monolithic carbon	Chemical bath deposition	Cathode for Li-S batteries	1 M LiTFSI + 0.1 M $LiNO_3$ OE	1350 mAh g^{-1} at 0.05 C	714 mAh g^{-1} at 0.05 C	61%/300	[16]
HKUST-1	HKUST-1		Chemical bath deposition			1377 mAh g^{-1} at 0.05 C	541 mAh g^{-1} at 0.05 C	82%/300	
HKUST-1	HKUST-1	No	Filtration + chemical bath deposition	Cathode for Li-S batteries	1 M LiTFSI + 1.0 wt% $LiNO_3$ OE	1102 mAh g^{-1} at 0.2 C	449 mAh g^{-1} at 10 C	60%/500	[17]

ZIF-8	N-doped porous carbon	No	Filtration + chemical bath deposition +annealing	Cathode for Li-S batteries	1 M LiTFSI + 1.0 wt% $LiNO_3$ OE	1235 mAh g^{-1} at 0.2 C	782 mAh g^{-1} at 5 C	64.1%/1800	18
ZIF-8	ZIF-8	carbon nanotube networks	Solvothermal method	Cathode for Li-S batteries	1 M LiTFSI + 1.0 wt% $LiNO_3$ OE	1480 mAh g^{-1} at 0.05 C	840 mAh g^{-1} at 1 C	89.3%/500	19
Co-MOF	Co_3O_4	CC	Filtration + chemical bath deposition +annealing	Cathode for Li-O_2 batteries	1 M LiTFSI OE	6509 mAh g^{-1} at 0.2 A g^{-1}			20
ZIF-9	Co_3O_4	No	Electrospinning + annealing	Cathode for Li-O_2 batteries	1 M $LiCF_3SO_3$ OE	760 mAh g^{-1} at 0.8 A g^{-1}			21
ZIF-67	Co-NC	CC	Chemical deposition +annealing	Anode for Li-O_2 batteries	1 M LiTFSI + 1.0 wt% $LiNO_3$ OE				22
Co-MOF	Co-NC	No	3D-printing+ annealing	Cathode for Li-O_2 batteries	0.5 M $LiClO_4$ OE	1124 mAh g^{-1} at 0.05 mA cm^{-2}	525 mAh g^{-1} at 0.8 mA cm^{-2}		23
ZIF-L	3D NC	No	3D-printing+ annealing	Anode for Li-O_2 batteries	1 M LiTFSI + 1.0 wt% $LiNO_3$ OE				24

CR: capacity retention, CN: cycle number, OE: organic electrolyte.

Table S2. Freestanding MOFs-based/-derived electrodes for SIBs.

MOF	Sample	Substrate	Preparation strategy	Application	Electrolyte	Capacity	Rate performance	CR/CN	Ref.
Co-MOF	NC@MoS_2	CC	Chemical deposition +annealing	Anode for SIBs	1 M $NaClO_4$ OE	660 mAh g^{-1} at 0.1 A g^{-1}	235 mAh g^{-1} at 2.0 A g^{-1}	75.3%/1000	[25]
ZIF-67	CoO_x@NC	No	Electrospinning + annealing	Anode for SIBs	1 M $NaClO_4$ OE	423 mAh g^{-1} at 0.2 A g^{-1}	141 mAh g^{-1} at 20 A g^{-1}	92.2%/6000	[26]
CuTCNQ	CuTCNQ	carbon nanofibers network	Template-assistance methods	Cathode for SIBs	1 M $NaClO_4$ OE	252 mAh g^{-1} at 0.03 A g^{-1}	89 mAh g^{-1} at 0.6 A g^{-1}	78%/1200	[27]
KNHCF	KNHCF	CNTF	Template-assistance methods	Cathode for SIBs	1 M Na_2SO_4 AE	58.54 mAh cm^{-3} at 0.05 A cm^{-3}	42.56 mAh cm^{-3} at 5.0 A cm^{-3}	90.2%/1000	[28]
KZHCF	KZHCF	CC	Template-assistance methods	Cathode for SIBs	1 M $NaClO_4$ AE	0.76 mAh cm^{-2} at 0.5 A cm^{-2}	0.44 mAh cm^{-2} at 20 A cm^{-2}	87.5%/300	[29]
Na_xM_n[Mn(CN)$_6$]	Na_xM_n[Mn(CN)$_6$]	Au-sputtered glass	Electrodeposition	Cathode for SIBs	10 M $NaClO_4$ AE	85 mAh g^{-1} at 5 A g^{-1}		97%/3000	[30]

Note: Prussian blue and its analogues are marked by undeiline.

Table S3. Freestanding MOFs-based/-derived electrodes for Zn-air batteries.

MOF	Sample	Substrate	Preparation strategy	Overpotential for OER (10 mA cm^{-2})	HW potential for ORR	Electrolyte	Open-circuit voltage	Voltage gap	Stability	Ref.
ZIF-67	Co_4N	CC	Chemical deposition + annealing	310 mV	0.8 V	6 M KOH + 0.2 M $Zn(Ac)_2$ AE	1.4 V	1.09 V at 50 mA cm^{-2}	408 cycles (136 h)	31
ZIF	D-ZIF	NF	Chemical deposition + annealing	370 mV	0.6 V	6 M KOH + 0.02 M $Zn(Ac)_2$ AE	1.38 V	0.78 V at 2 mA cm^{-2}	330 h	32
Ni-BTC	Ni@N-HCGHF	No	Filtration + annealing	260 mV	0.875 V	6 M KOH + 0.2 M $Zn(Ac)_2$ AE	1.49 V		100 cycles	33
Co-BTC	Co-N-C	Cobalt plate	Electrodeposition + annealing	310 mV		6 M KOH + 0.2 M $Zn(Ac)_2$ AE	1.49 V	~0.63 V at 3 mA cm^{-2}	45 cycles	34
Co-MOF	NC-Co SA	CC	Chemical deposition + annealing	360 mV	0.87 V	6 M KOH + 0.1 M $Zn(Ac)_2$ AE	1.411 V	0.45 V at 10 mA cm^{-2} (solid)	570 cycles (180 h)	35
CoZn-MOF	P-$CoSe_2$/N-C	CC	Chemical deposition + annealing	230 mV	0.87 V	11.25 M KOH + 0.25 M ZnO SE	1.30 V (solid)	~1 V at 1 mA cm^{-2} (solid)	~26.7 h (solid)	36
ZIF-67	CCVG@CoNCNT	CC	Chemical deposition + annealing	357 mV	~0.57 V	6 M KOH + 0.2 M $Zn(Ac)_2$ SE	1.35 V (solid)	1.72 V at 60 mA cm^{-2} (solid)	25 h (solid)	37
ZIF-L	Co SA@NCF	CNF	Chemical deposition + annealing	400 mV	0.88 V	1.6 M KOH + 0.002 M $Zn(Ac)_2$ SE	1.41 V (solid)		90 cycles (solid)	38
Zn/co-ZIF	HCA-Co	CC	Chemical deposition + annealing	290 mV	0.87 V	11 M KOH + 0.75 M ZnO + 1 M Na_2SnO_3 + 0.05 M $In(OH)_3$ SE	1.40 V (solid)		~33.3 h (solid)	39
Co-MOF	CoP_x@CNS	NF	Chemical deposition + annealing	286 mV at 50 mA cm^{-2}	0.76 V	6 M KOH + 0.2 M $Zn(Ac)_2$ AE	1.40 V	~0.7 V at 5 mA cm^{-2}	400 cycles (130 h)	40
ZIF-67	Co_3O_4–C	NF	Template-assistance method + annealing	310 mV	0.83 V	6 M KOH AE	1.42 V	1.01 V at 30 mA cm^{-2}	180 cycles	41
ZIF-L	Co_3O_4@N-CNMAs	CC	Chemical deposition + annealing	310 mV	0.9 V	6 M KOH + 0.1 M $Zn(Ac)_2$ AE	1.66 V	0.83 V at 5 mA cm^{-2}	384 h	42
ZIF	CoNCNTF	CNT	Chemical deposition + annealing	380 mV	0.857 V	1.6 M KOH + 0.002 M $Zn(Ac)_2$ SE	1.34 V (solid)	0.29 V at 0.5 mA cm^{-2}	68 cycles	43
Co-MOF	NC-Co_3O_4	CC	Chemical deposition + annealing	358 mV	0.87 V	11.25 M KOH + 0.25 M ZnO SE	1.44 V (solid)	~0.85 V at 10 mA cm^{-2} (solid)	600 cycles (~210 h) (solid)	44
KFeCo PBA	FeCo/FeCoNi	No	Electrospinning + annealing	378 mV	0.85 V	6 M KOH + 0.2 M $Zn(Ac)_2$ AE	1.481 V	0.68 V at 5 mA cm^{-2}	360 cycle (240 h)	45
Co/Mn-ZIF	Co_3O_4/Mn_3O_4/CN_x	CNFs	Chemical deposition + annealing	400 mV	0.85 V	6 M KOH AE	1.518 V	1.16 V at 5 mA cm^{-2}	50 h	46

ZIF-67	Co/N-C	CNFs	Chemical deposition + annealing	~526 mV	0.79 V	6 M KOH AE	1.53 V	~1 V at 5 mA cm^{-2}	300 cycles	[47]
ZIF-67	Co/Co-N-C	Carbon felt	Template-assistance method + annealing	310 mV	~0.84 V	6 M KOH + 0.1 M $Zn(Ac)_2$ AE	1.41 V	0.82 V at 10 mA cm^{-2}	1000 cycles	[48]
Co/Fe-MOF	Fe-Co_4N@N-C	CC	Chemical deposition + conversion method + annealing	390 mV	0.83 V	6 M KOH + 0.2 M $Zn(Ac)_2$ AE	1.46 V	~0.8 V at 5 mA cm^{-2}	220 cycles	[49]
ZIF-8	Co-N_x/C	Ti foils	Hydrothermal + annealing	300 mV	0.877 V	6 M KOH + 0.2 M $Zn(Ac)_2$ AE	1.42 V	~0.9 V at 50 mA cm^{-2}	80 h	[50]
ZIF-67	CoN_x	Graphene aerogel	Chemical deposition + annealing	295 mV	0.83 V	6 M KOH + 0.2 M $Zn(Ac)_2$ AE	1.33 V	0.79 V at 50 mA cm^{-2}	12 h	[51]
ZIF-L	NC-Co/CoN_x	CC	Chemical deposition + annealing	289 mV	0.87 V	11.25 M KOH + 0.25 M ZnO SE	1.40 V (solid)		25 h (solid)	[52]
Co-ZIF	NP-Co_3O_4/C	CC	Chemical deposition + annealing	~330 mV	0.9 V	6 M KOH AE	1.576 V	1.02 V at 100 mA cm^{-2}	400 h	[53]
ZIF-67	Co@NPCFs	CNFs	Chemical deposition + annealing	400 mV	0.66 V	6 M KOH + 0.2 M $Zn(Ac)_2$ AE	1.44 V	1.16 V at 5 mA cm^{-2}	480 cycles (80 h)	[54]
ZIF-67	s-Co@NCP	rGO	Adsorption + annealing		0.81 V	6 M KOH AE	1.466 V		~69 h	[55]

HW: half-wave, SE: solid-state electrolyte, SA: single atom, CNF: carbon nanofiber.

Note: Prussian blue and its analogues are marked by undeiline

Table S4. Freestanding MOFs-based/-derived electrodes for Zn-NiCo and ZIBs.

MOF	Sample	Substrate	Preparation strategy	Application	Electrolyte	Capacity	Rate performance	CR/CN	Ref.
ZIF-67	$CoSe_{2-x}$	CC	Chemical deposition +annealing	Cathode for Zn-Co battery	5 M KOH + 0.02 M $Zn(Ac)_2$ AE	~11.2 mAh cm^{-2} at 4 mA cm^{-2}	~7.42 mAh cm^{-2} at 10 mA cm^{-2}	72.4%/10000	[56]
Co-MOF	NiCo-DH	NF	Chemical deposition + ion exchange	Cathode for Zn-Ni battery	ZnO saturated 2.5 M KOH AE	329 mAh g^{-1} at 0.5 mA cm^{-2}	204 mAh g^{-1} at 15 mA cm^{-2}	73%/850	[57]
Ni-MOF-74	Ni-MOF-74	CNTFs	Solvothermal method	Cathode for Zn-Ni battery	ZnO saturated 2 M KOH AE	184.5 mAh cm^{-3} at 0.25 A cm^{-3}	147.6 mA h cm^{-3} at 5.0 A cm^{-3}	86.2%/2000	[58]
Ag-MOF	Ag	CC	Chemical deposition + annealing	Cathode for Zn-Ag battery	1 M KOH AE	~1.605 mAh cm^{-2} at 0.2 mA cm^{-2}	~1.32 mAh cm^{-2} at 2 mA cm^{-2}	90%/70	[59]
Ni-MOF	Ni-MOF	CNTF	Solvothermal method	Cathode for Zn-Ni battery	ZnO saturated 2 M KOH AE	0.4 mAh cm^{-2} at 0.5 mA cm^{-2}	0.315 mAh cm^{-2} at 5 mA cm^{-2}	89%/600	[60]
Mn-MIL-100	Mn_2O_3@C	CNTF	Solvothermal method + annealing	Cathode for ZIBs	2 M $ZnSO_4$ + 0.5 M $MnSO_4$ AE	154.9 mAh cm^{-3} at 0.3 A cm^{-3}	90.3 mAh cm^{-3} at 3.0 A cm^{-3}	79.6%/3000	[61]
V-MIL-47	V-MIL-47	CNTF	Solvothermal method	Cathode for ZIBs	2 M $ZnCl_2$ AE	101.8 mAh cm^{-3} at 0.1 A cm^{-3}	65.5 mAh cm^{-3} at 5.0 A cm^{-3}	84.6%/400	[62]
Mn-MOF	Od-Mn_3O_4@C	CC	Solvothermal method + annealing	Cathode for ZIBs	2 M $ZnSO_4$ + 0.2 M $MnSO_4$ AE	396.2 mAh g^{-1} at 0.2 A g^{-1}	143 mAh g^{-1} at 5 A g^{-1}	95.7%/12000	[63]

Table S5. Freestanding MOFs-based/-derived electrodes for supercapacitors.

MOF	Sample	Substrate	Preparation strategy	Electrolyte	Potential window	Capacitance	Rate performance	CR/CN	Ref.
ZIF-L	Ni/Co-N	CC	Chemical deposition + annealing	1 M KOH AE	0-0.5 V	361.93 C g^{-1} at 2 mA cm^{-2}	207.6 C g^{-1} at 50 Ma cm^{-2}		[64]
Co-MOF	$ZnCo_2O_4$ @NC	Carbon textiles	Chemical deposition + ion exchange + annealing	3 M KOH AE	-0.5-4.5 V	2.244 F cm^{-2} at 2 Ma cm^{-2}	1.676 F cm^{-2} at 64 Ma cm^{-2}	~99.37%/10000	[65]
Co-MOF	P-Co_3O_4@PNC	Carbon fibers	Chemical deposition + annealing	2 M KOH AE	0-0.4 V	1.023 F cm^{-2} at 1 Ma cm^{-2}	0.806 F cm^{-2} at 30 Ma cm^{-2}	96.9%/10000	[66]
Ni−HITP	Ni−HITP	CNFs	Hydrothermal method + filtration	3 M KCl AE	0-0.7 V	125 F g^{-1} at 0.33 A g^{-1}	87.5 F g^{-1} at 33 A g^{-1}		[67]
Cu-CAT	Cu-CAT	Carbon papers	Chemical deposition	3 M KCl AE	0-0.5 V	202 F g^{-1} at 0.5 A g^{-1}	134 F g^{-1} at 10 A g^{-1}	80%/5000	[68]
ZIF-8	NC	No	Electrospinning + annealing	2 M H_2SO_4 AE	0-1 V	307.2 F g^{-1} at 1.0 A g^{-1}	193.4 F g^{-1} at 50.0 A g^{-1}	98.2%/10000	[69]
Co-MOF	Cu(Co-Ni)$_2S_4$	NF	Chemical deposition + ion exchange + annealing	1 M KOH AE	0-0.4 V	0.38 mAh cm^{-2} at 2 Ma cm^{-2}	0.2 mAh cm^{-2} at 30 Ma cm^{-2}	96.2%/5000	[70]
Cu-CAT	Cu-CAT	Ppy	Hydrothermal method	3 M KCl AE	0-0.5 V	116 F g^{-1} at 1.25 Ma cm^{-2}	71 F g^{-1} at 25 Ma cm^{-2}	87%/5000	[71]
CoNi-MOF	CoNi-MOF	Carbon paper	Template-assistance method	1 M KOH AE	0-0.45 V	1044 F g^{-1} at 2 A g^{-1}	569 F g^{-1} at 32 A g^{-1}		[72]
MIL-88-Fe	S-α-Fe_2O_3@C	CNTFs	Solvothermal method + annealing	1 M Na_2SO_4 AE	-1.0-0 V	1.23 F cm^{-2} at 2 Ma cm^{-2}	0.78 F cm^{-2} at 20 Ma cm^{-2}	97.6%/4000	[73]
Co-MOF	$NiCo_2O_4$	CC	Chemical deposition + ion exchange + annealing	2 M KOH AE	0-0.6 V	1055.3 F g^{-1} at 2.5 Ma cm^{-2}	483.3 F g^{-1} at 60 Ma cm^{-2}		[74]
ZIF-67	Porous carbon	No	Electrospinning + annealing	2 M H_2SO_4 AE	0-1.0 V	421 F g^{-1} at 0.5 A g^{-1}	264 F g^{-1} at 20 A g^{-1}		[75]
CoNi-MOF	CoNi-MOF	Ni film	Template-assistance method + solvothermal method	6 M KOH AE	0-0.5 V	813 C cm^{-3} at 0.5 A cm^{-3}	325 C cm^{-3} at 5 A cm^{-3}		[76]
Ni-MOF	Ni-MOF	CC	Solvothermal method	2 M KOH AE	0-0.4 V	208.8 mAh g^{-2} at 2 Ma cm^{-2}	142.4 mAh g^{-2} at 20 Ma cm^{-2}		[77]
CuCo-MOF	Cu-Co_9S_8	NF	Template-assistance method + solvothermal method	6 M KOH AE	0-0.5 V	2636 F g^{-1} at 2 A g^{-1}	1584 F g^{-1} at 30 A g^{-1}	94%/5000	[78]
UiO-66	UiO-66	Carbon fibers	electrodeposition	3 M KCl AE	-0.4-0.6 V	15 Mf cm^{-1} at 5 Mv s^{-1}	8 Mf cm^{-1} at 100 Mv s^{-1}	96%/1200	[79]
ZIF-67	Co_3O_4/NC	CC	Chemical vapor method + annealing	6 M KOH AE	0-0.5 V	1.22 F cm^{-2} at 0.5 Ma cm^{-2}	0.72 F cm^{-2} at 20 Ma cm^{-2}	98.2%/4000	[80]
Co-ZIF	NiCo-LDH	Carbon foam	Chemical deposition + ion exchange	2 M KOH AE	0-0.5 V	756 C g^{-1} at 0.5 A g^{-1}	414 C g^{-1} at 20 A g^{-1}	81.7%/5000	[81]
Co-Ni-	Co_3O_4-	Graphene	Chemical deposition +	2 M KOH	0.1-0.4 V	783 F g^{-1} at	530 F g^{-1} at 10	84%/5000	[82]

CMF	NiO	foam	annealing	AE		0.5 A g^{-1}	A g^{-1}		
Zn/Co ZIF-L	$Zn_{0.76}Co_{0.24}S/NiCo_2S_4$	CC	Chemical deposition + ion exchange + annealing	2 M KOH AE	0-0.5 V	2674 F g^{-1} at 1 A g^{-1}	2112 F g^{-1} at 20 A g^{-1}	93%/2000	83
Co-MOF	Co-MOF	CC	Chemical deposition + ion exchange	1 M LiOH	0-0.5 V	803 F g^{-1} at 0.5 Ma cm^{-2}	415 F g^{-1} at 15 Ma cm^{-2}	90%/15000	84
Mn-BTC	Mn_2O_3	graphene network	Chemical deposition + annealing	0.5 M Na_2SO_4 AE	0-0.8 V	471.1 F g^{-1} at 0.2 A g^{-1}	270.0 F g^{-1} at 5 A g^{-1}	~100%/1800	85
Co-MOF	$CoSe_2$/NC	NF	Chemical deposition + annealing	6 M KOH AE	0-0.35 V	120.2 mAh g^{-2} at 1 A g^{-1}	73.6 mAh g^{-2} at 20 A g^{-1}	92%/10000	86
HKUST-1	Porous carbon	No	Filtration + annealing	6 M KOH AE	-1.1- -0.1 V	194.8 F g^{-1} at 2 A g^{-1}	120.9 F g^{-1} at 100 A g^{-1}	95%/10000	87
ZIF-L	NC	Carbon foam	Chemical deposition + annealing	6 M KOH AE	-1- 0 V	238 F g^{-1} at 1 A g^{-1}	194.8 F g^{-1} at 2 A g^{-1b}		88
Co-MOF	Co_3O_4/C	NF	Hydrothermal method + annealing	3 M KOH AE	-0.1-0.4 V	776.5 F g^{-1} at 1 Ma cm^{-2}	635.3 F g^{-1} at 20 Ma cm^{-2}	96%/2000	89
Co/Zn-MOF	Co/Zn-S	No	Filtration + annealing	6 M KOH AE	0-0.5 V	1640 F g^{-1} at 1 A g^{-1}	1076.4 F g^{-1} at 10 A g^{-1}		90
Mn-MOF	Mn-MOF	NF	Solvothermal method	2 M KOH AE	0-0.6 V	567.5 mAh g^{-2} at 1 A g^{-1}	317.5 mAh g^{-2} at 12 A g^{-1}	92.3%/5000	91
ZIF-67	$NiCo_2S_4$	NF	Chemical deposition + ion exchange + Hydrothermal method	6 M KOH AE	0-0.4 V	939 C g^{-1} at 1 A g^{-1}	712 C g^{-1} at 10 A g^{-1}	92.8%/5000	92
	CoS_2		Chemical deposition + Hydrothermal method + annealing		-1.1 - -0.3 V	343.5 C g^{-1} at 1 A g^{-1}	146.3 C g^{-1} at 20 A g^{-1}		
Co-MOF	NiCo-A-S	CC	Chemical deposition + ion exchange + annealing + Hydrothermal method	3 M KOH AE	0-0.5 V	213 mAh g^{-2} at 1 A g^{-1}	171 mAh g^{-2} at 20 A g^{-1}	86%/5000	93
Ni-MOF	NiCoS	NF	Hydrothermal method	1 M KOH AE	0-0.4 V	2815.4 F g^{-1} at 1 Ma cm^{-2}	1053.6 F g^{-1} at 30 Ma cm^{-2}	43%/3000	94
Co-MOF	NiCo-LDH /Co_9S_8	CC	Chemical deposition + ion exchange + Hydrothermal method	6 M KOH AE	0-0.4 V	2850 F g^{-1} at 1 A g^{-1}	2063 F g^{-1} at 10 A g^{-1}	92.6%/5000	95
Co-MOF	Co_3O_4	CC	Chemical deposition + electrochemical oxidation	1 M KOH AE	0-0.45 V	226.1 C g^{-1} at 1.3 A g^{-1}	219.6 C g^{-1} at 8.9 A g^{-1}	77%/5000	96
Co-MOF	$CoMoO_4$	NF	Solvothermal method + ion exchange	3 M KOH AE	0-0.5 V	12.2 F cm^{-2} at 2 Ma cm^{-2}	10 F cm^{-2} at 50 Ma cm^{-2}	90.5%/5000	97
ZIF-67	CoS_2	Cu foam	Chemical deposition + annealing + chemical bath	6 M KOH AE	0-0.4 V	2185 F g^{-1} at 1 Ma cm^{-2}	1785 F g^{-1} at 50 Ma cm^{-2}	~100%/10000	98
Zn/Co-MOF	Zn-Co-P	NF	Chemical deposition + annealing	6 M KOH AE	0-0.45 V	2115.5 F g^{-1} at 1 A g^{-1}	1086.5 F g^{-1} at 50 A g^{-1}	80.3%/7000	99

Table S6. Freestanding MOF based electrodes for HER and OER.

MOF	Sample	Substrate	Preparation strategy	Electrolyte	application	Onset potential/V	Overpotential/mV	Tafel/ mV dec^{-1}	Stability	Ref.
NiCo-BDC	NiCo-BDC	NF	Template-assistance method	1 M KOH AE	OER		230	61	24 h	100
MOF-74-Co/Fe	$CoFe_2O_4$/C	NF	Solvothermal method + annealing	1 M KOH AE	OER	1.45	240	45	0.42%/30 h	101
$FeCo_{0.5}Ni_{0.5}$-MOF	$FeCo_{0.5}Ni_{0.5}$-LDH	Cu foil	Chemical deposit + ion exchange	1 M KOH AE	OER		248	38	50 h	102
Co-BDC	Co_3O_4/C	Ni foil	Solvothermal method + annealing	1 M KOH AE	OER		208	50.1	36 h	103
Cu-TDC	Cu_2O-S/C	Cu foil					313	65.6	60	
Co-MOF	Co_3O_4/C	Cu foil	Chemical deposit + annealing	0.1 M KOH AE	OER	1.47	290	70	6.5%/30 h	104
Fe/Ni/Co-MIL-53	Fe/Ni/Co-MIL-53	NF	Solvothermal method	1 M KOH AE	OER		219	53.5	1000 cycles	105
MIL-53 (FeNi)	MIL-53 (FeNi)	NF	Solvothermal method	1 M KOH AE	OER		233 (50 mA cm^{-2})	39.6	~13.3 h	106
KNi[Fe(CN)$_6$]	$Ni_2P/(NiFe)_2P(O)$	NF	Template-assistance method + annealing	1 M KOH AE	OER		150	60	50 h	107
CoNi-MOF	CoNi-MOF	NF	Solvothermal method	1 M KOH AE	OER	1.41	215	51.6	300 h	108
Fe/Ni-BTC	Fe/Ni-BTC	NF	Electrodeposition	0.1 M KOH AE	OER	1.40	270	47	15 h	109
ZnCo-MOF	Zn-$CoSe_2$	CC	Chemical deposit + annealing	1 M KOH AE	OER	~1.35	356	88	1%/14 h	110
Ni-BDC	Ni-BDC @NiS	NF	Solvothermal method	1 M KOH AE	OER	1.52	340 (20 mA cm^{-2})	62	12 h	111
NiFe-PBA	Fe-NiO	CC	Template-assistance method + annealing	1 M KOH AE	OER		218	47	50 h	112
Co-MOF	$CoSe_2$/C	CC	Chemical deposit + annealing	0.5 M H_2SO_4 AE	HER		84	38	72 h	113
Co-MOF	W-CoP	CC	Ion exchange + annealing	0.5 M H_2SO_4 AE	HER	0.031	89	58	36 h	114
CoW-MOF	S-CoWP @S,N-C	CC	Solvothermal method + annealing	0.5 M H_2SO_4 AE	HER		146	68	3%/40 h	115
NiRu-MOF	NiRu-MOF	NF	Solvothermal method	1 M KOH AE	HER		156	90	24 h	116
Co-MOF	Mo-CoP/NC	Ti foil	Chemical deposit + ion exchange + annealing	0.5 M H_2SO_4 AE	HER	0.004	59	51.2	24 h	117
Ni-ZIF	Ni-ZIF/Ni-B	NF	Hydrothermal + chemical bath	1 M KOH AE	OER		234	57	36 h	118
					HER		67	108	64 h	
Co-Fe PBA	Fe-CoP	NF	Chemical deposit + annealing	1 M KOH AE	OER		190	36	30 h	119
					HER		78	92	30 h	
Co-MOF	Mo-CoP	CC	Ion exchange + annealing	1 M KOH AE	OER		305	56	20 h	120
					HER		40	65	20 h	
NiFe-	NiFe-	NF	Solvothermal method	1 M KOH AE	OER		230 (50	32	17 h	121

MOF	MS/MOF						mA cm^{-2})			
					HER		90	82	28 h	
ZIF-67	CoNC	NF	Template-assistance method + annealing	1 M KOH AE	OER		309	53	3000 cycles	122
					HER		190	98		
NiFe-MOF	NiFe-MOF	NF	Hydrothermal method	0.1 M KOH AE	OER		240	34	~5.6 h	123
					HER		134		2000 s	
CoFe-PBA	CoFe-PBA	NF	Hydrothermal method	1 M KOH AE	OER		256	54	24 h	124
					HER		48	66		
ZIF-67	CNT	NF	Electrodeposition + annealing	1 M KOH AE	OER		286	62	~6 h	125
					HER		133	96		
CPO-27-Ni	CPO-27-Ni	NF	Template-assistance method + annealing	1 M KOH AE	OER		295	52	0.3%/30 h	126
					HER		49.48	74	3.7%/30 h	
Co-MOF	CoP	NF	Chemical deposit + ion exchange + annealing	1 M KOH AE	OER		317 (50 mA cm^{-2})	65.6	12 h	127
					HER		41.1	65.3	15 h	
Co-MOF	NC-CNT/CoP	CC	Chemical deposit + annealing	1 M KOH AE	OER		240		20 h	128
					HER		120	73	15%/20 h	
NiFe-MOF	NiFe-MOF	NF	Solvothermal method	1 M KOH AE	OER		198	30.6	40 h	129
					HER		142	94.7	50 h	
Co-MOF	Co-Pt/C	CC	Chemical deposit + annealing	1 M KOH AE	OER		320	72	10 h	130
					HER	0.035	50	46	10 h	
FeCo-PBA	FeCo/C	NF	Template-assistance method + annealing	1 M KOH AE	OER		219	74		131
	FeCoP/C				HER		55	107		
Co-MOF	Ni_2P-Co_2P@C	NF	Chemical deposit + ion exchange + annealing	1 M KOH AE	OER		290 (50 mA cm^{-2})	64	20 h	132
					HER		167 (50 mA cm^{-2})	68	20 h	
Ni-9AC-AD	Ni-9AC-AD	NF	Hydrothermal method	1 M KOH AE	OER		350 (50 mA cm^{-2})	51.3	30 h	133
					HER		143	79.5	30 h	

PKi: phosphate solution.

Note: Prussian blue and its analogues are marked by undeiline

Table S7 Comparisons between freestanding electrodes and traditional powder-form electrodes

Differences	Freestanding MOF based electrodes	Traditional power-form MOF based electrode
**Electrode preparation**	MOF-based active materials grown on conductive substrates or formed a freestanding film directly as electrodes.	MOF-based active materials coated on conductive substrates by slurry-coasting methods.
**Additives**	No	Unactive binders and conductive additives
**Substrate selections**	Diversification. 1D: carbon nanotube fibers; 2D: carbon cloths, carbon papers, metal foils; 3D: nickel foams, 3D graphene foam	Limited selections. 2D Al/Cu foils or carbon cloths as main option
**Electrode configuration**	(i) Nanorod/sheets/wires/wall arrays<br>(ii) 3D architecture with porous structure	Thin film form without ordered nanostructure arrays
**Active sites**	Abundant and accessible active sites	Limited active sites due to the coverage of polymer binders and the stacking of active materials
**Ion diffusion**	3D nanostructure arrays or porous structures for fast ion diffusion	Dense accumulation for sluggish ion diffusion rate
**Electron transportation**	Seamless contact between active materials and conductive substrates or 3D continuous conductive network for fast electron transportation	Interspersion of insulating adhesive and poor contact between active materials and conductive substrates resulting in large electron transfer resistance
**Stability for energy storage**	Enough free space accommodating volume expansion of active materials during charge-discharge process to restrain the decay of the capacity	Easily falling off during the cycle test due to the dense electrode configuration
**Stability for OER and ORR**	Stable, owning to the strong adhesion with substrates and open array structures	Easily falling off with the generation of gas bubbles owning to the poor contact with substrates
**Mechanism research**	Accurate platform to study the transformation of substances during the energy storage and conversion due to the absence of additives	Existence of interference in the precise study of the structures, valences and performances of active materials due to the introduction of conductive agents and binders
**Wearable application**	Good mechanical flexibility due to the existence of the space among nanostructure units and seamless contact with the flexible substrates	Poor mechanical flexibility because of the easily cracking and peeling of active materials after bending multiple times

Table S8 Electrochemical applications and the corresponding properties required.

**Applications**	**Required properties**	**Freestanding MOFs (PBAs)**	**Freestanding MOF derivatives**
Li/Na/Zn-ion Batteries	➢ Abundant redox active sites;<br>➢ Regular channels and porous structure;<br>➢ Excellent conductivity.	CPO-27, KZHCF (PBAs), CuTCNQ	ZnO (ZIF-8), Co_3O_4 (ZIF-67), Mn_2O_3@C (Mn-MIL-100)
Li/Zn-air batteries	➢ Nanostructure active species (nitrides, cardides, oxides, and metal atoms) for OER and ORR;<br>➢ Excellent conductivity and porous structure.	D-ZIF	NC-Co/CoNx (ZIF-L), NC-Co_3O_4 (Co-MOF),<br>Co-NC (ZIF-67)
Li-S batteries	➢ Hindering the shuttle effect of soluble LiPSs;<br>➢ High pore volume to accommodate volume variation;<br>➢ Excellent conductivity.	ZIF-67, HKUST-1, ZIF-8	N-doped porous carbon (ZIF-8), Co/N-PCN@rGO (ZIF-67)
Supercapacitors	➢ Abundant redox active sites;<br>➢ Large accessible surface areas;<br>➢ Excellent conductivity.	Ni-HITP, Cu-CAT, UiO-66	$NiCo_2O_4$ (Co-MOF), Mn_2O_3 (Mn-BTC), Co_3O_4 (ZIF-67)
Electrochemical water splitting	➢ Nanostructure active species (nitrides, cardides, oxides, and metal atoms) for OER and HER;<br>➢ Excellent conductivity and porous structure.	NiCo-BDC, MIL-53 (FeNi), CoFe-PBA, Fe/Ni-BTC	Fe-NiO (NiFe-PBA), Mo-CoP (Co-MOF), CoNC (ZIF-67)

References：

(1) Samuel, E.; Joshi, B.; Kim, M.-W.; Kim, Y.-I.; Park, S.; Kim, T.-G.; Swihart, M. T.; Yoon, W. Y.; Yoon, S. S. Zeolitic Imidazolate Framework-8 Derived Zinc Oxide/Carbon Nanofiber as Freestanding Electrodes for Lithium Storage in Lithium-Ion Batteries. *J. Power Sources* **2018,** *395*, 349-357.

(2) Du, M.; Song, D.; Huang, A.; Chen, R.; Jin, D.; Rui, K.; Zhang, C.; Zhu, J.; Huang, W. Stereoselectively Assembled Metal-Organic Framework (MOF) Host for Catalytic Synthesis of Carbon Hybrids for Alkaline-Metal-Ion Batteries. *Angew. Chem. Int. Ed.* **2019,** *58*, 5307-5311.

(3) Lin, J.; Zeng, C.; Wang, L.; Pan, Y.; Lin, X.; Reddy, R. C. K.; Cai, Y.; Su, C.-Y. Self-Standing MOF-Derived $LiCoO_2$ Nanopolyhedron on Au-Coated Copper Foam as Advanced 3D Cathodes for Lithium-Ion Batteries. *Appl. Mater. Today* **2020,** *19*, 100565.

(4) Fang, G.; Zhou, J.; Liang, C.; Pan, A.; Zhang, C.; Tang, Y.; Tan, X.; Liu, J.; Liang, S. MOFs Nanosheets Derived Porous Metal Oxide-Coated Three-Dimensional Substrates for Lithium-Ion Battery Applications. *Nano Energy* **2016,** *26*, 57-65.

(5) Zhang, H.; Wang, Y.; Zhao, W.; Zou, M.; Chen, Y.; Yang, L.; Xu, L.; Wu, H.; Cao, A. MOF-Derived ZnO Nanoparticles Covered by N-Doped Carbon Layers and Hybridized on Carbon Nanotubes for Lithium-Ion Battery Anodes. *ACS Appl. Mater. Inter.* **2017,** *9*, 37813-37822.

(6) Sekhar, S. C.; Ramulu, B.; Narsimulu, D.; Arbaz, S. J.; Yu, J. S. Metal-Organic Framework-Derived $Co_3V_2O_8$@CuV_2O_6 Hybrid Architecture as a Multifunctional Binder-Free Electrode for Li-Ion Batteries and Hybrid Supercapacitors. *Small* **2020,** *16*, 2003983.

(7) Liu, T.; Wang, W.; Yi, M.; Chen, Q.; Xu, C.; Cai, D.; Zhan, H. Metal-Organic Framework Derived Porous Ternary $ZnCo_2O_4$ Nanoplate Arrays Grown on Carbon Cloth as Binder-Free Electrodes for Lithium-Ion Batteries. *Chem. Eng. J.* **2018,** *354*, 454-462.

(8) Luo, F.; Ma, D.; Li, Y.; Mi, H.; Zhang, P.; Luo, S. Hollow Co_3S_4/C Anchored on Nitrogen-Doped Carbon Nanofibers as a Free-Standing Anode for High-Performance Li-Ion Batteries. *Electrochim. Acta* **2019,** *299*, 173-181.

(9) Ji, D.; Zhou, H.; Tong, Y.; Wang, J.; Zhu, M.; Chen, T.; Yuan, A. Facile Fabrication of MOF-Derived Octahedral CuO Wrapped 3D Graphene Network as Binder-Free Anode for High Performance Lithium-Ion Batteries. *Chem. Eng.g J.* **2017,** *313*, 1623-1632.

(10) Yin, D.; Huang, G.; Na, Z.; Wang, X.; Li, Q.; Wang, L. CuO Nanorod Arrays Formed Directly on Cu Foil from MOFs as Superior Binder-Free Anode Material for Lithium-Ion Batteries. *ACS*

Energy Lett. **2017,** *2*, 1564-1570.

(11) Du, M.; Rui, K.; Chang, Y.; Zhang, Y.; Ma, Z.; Sun, W.; Yan, Q.; Zhu, J.; Huang, W. Carbon Necklace Incorporated Electroactive Reservoir Constructing Flexible Papers for Advanced Lithium-Ion Batteries. *Small* **2018,** *14*, 1702770.

(12) Zhao, G.; Sun, X.; Zhang, L.; Chen, X.; Mao, Y.; Sun, K. A Self-Supported Metal-Organic Framework Derived Co_3O_4 Film Prepared by an In-Situ Electrochemically Assistant Process as Li Ion Battery Aanodes. *J. Power Sources* **2018,** *389*, 8-12.

(13) Zhou, D.; Ni, J.; Li, L. Self-Supported Multicomponent CPO-27 MOF Nanoarrays as High-Performance Anode for Lithium Storage. *Nano Energy* **2019,** *57*, 711-717.

(14) Wang, R.; Chen, Z.; Sun, Y.; Chang, C.; Ding, C.; Wu, R. Three-Dimensional Graphene Network-Supported Co, N-Codoped Porous Carbon Nanocages as Free-Standing Polysulfides Mediator for Lithium-Sulfur Batteries. *Chem. Eng. J.* **2020,** *399*, 125686.

(15) Liu, Y.; Li, G.; Fu, J.; Chen, Z.; Peng, X. Strings of Porous Carbon Polyhedrons as Self-Standing Cathode Host for High-Energy-Density Lithium-Sulfur Batteries. *Angew. Chem. Int. Ed.* **2017,** *56*, 6176-6180.

(16) Liu, B.; Bo, R.; Taheri, M.; Di Bernardo, I.; Motta, N.; Chen, H.; Tsuzuki, T.; Yu, G.; Tricoli, A. Metal-Organic Frameworks/Conducting Polymer Hydrogel Integrated Three-Dimensional Free-Standing Monoliths as Ultrahigh Loading Li-S Battery Electrodes. *Nano Lett.* **2019,** *19*, 4391-4399.

(17) Mao, Y.; Li, G.; Guo, Y.; Li, Z.; Liang, C.; Peng, X.; Lin, Z. Foldable Interpenetrated Metal-Organic Frameworks/Carbon Nanotubes Thin Film for Lithium-Sulfur Batteries. *Nat. Commun.* **2017,** *8*, 14628.

(18) Liu, Y.; Li, G.; Chen, Z.; Peng, X. CNT-Threaded N-Doped Porous Carbon Film as Binder-Free Electrode for High-Capacity Supercapacitor and Li-S Battery. *J. Mater. Chem. A* **2017,** *5*, 9775-9784.

(19) Zhang, H.; Zhao, W.; Zou, M.; Wang, Y.; Chen, Y.; Xu, L.; Wu, H.; Cao, A. 3D, Mutually Embedded MOF@Carbon Nanotube Hybrid Networks for High-Performance Lithium-Sulfur Batteries. *Adv. Energy Mater.* **2018,** *8*, 1800013.

(20) Gong, H.; Wang, T.; Xue, H.; Lu, X.; Xia, W.; Song, L.; Zhang, S.; He, J.; Ma, R. Spatially-Controlled Porous Nanoflake Arrays Derived from MOFs: An Efficiently Long-Life Oxygen Electrode. *Nano Res.* **2019,** *12*, 2528-2534.

(21) Song, M. J.; Kim, I. T.; Kim, Y. B.; Shin, M. W. Self-Standing, Binder-Free Electrospun Co_3O_4/Carbon Nanofiber Composites for Non-Aqueous Li-Air Batteries. *Electrochim. Acta* **2015,**

182, 289-296.

(22) Jiang, G.; Jiang, N.; Zheng, N.; Chen, X.; Mao, J.; Ding, G.; Li, Y.; Sun, F.; Li, Y. MOF-Derived Porous Co_3O_4-NC Nanoflake Arrays on Carbon Fiber Cloth as Stable Hosts for Dendrite-Free Li Metal Anodes. *Energy Storage Mater.* **2019,** *23*, 181-189.

(23) Lyu, Z.; Lim, G. J. H.; Guo, R.; Kou, Z.; Wang, T.; Guan, C.; Ding, J.; Chen, W.; Wang, J. 3D-Printed MOF-Derived Hierarchically Porous Frameworks for Practical High-Energy Density Li-O_2 Batteries. *Adv. Funct. Mater.* **2019,** *29*, 1806658.

(24 Lyu, Z.; Lim, G. J. H.; Guo, R.; Pan, Z.; Zhang, X.; Zhang, H.; He, Z.; Adams, S.; Chen, W.; Ding, J.; et al. 3D-Printed Electrodes for Lithium Metal Batteries with High Areal Capacity and High-Rate Capability. *Energy Storage Mater.* **2020,** *24*, 336-342.

(25) Ren, W.; Zhang, H.; Guan, C.; Cheng, C. Ultrathin MoS_2 Nanosheets@Metal Organic Framework-Derived N-Doped Carbon Nanowall Arrays as Sodium Ion Battery Anode with Superior Cycling Life and Rate Capability. *Adv. Funct. Mater.* **2017,** *27*, 1702116.

(26) Yang, C.; Li, Y.; Zhang, B.; Lian, Y.; Ma, Y.; Zhao, X.; Zeng, X.; Li, J.; Deng, Z.; Ye, J.; et al. Nitrogen-Doped Carbon Fibers Embedding CoO_x Nanoframes Towards Wearable Energy Storage. *Nanoscale* **2020,** *12*, 8922-8933.

(27) Huang, Y.; Fang, C.; Zeng, R.; Liu, Y.; Zhang, W.; Wang, Y.; Liu, Q.; Huang, Y. In Situ-Formed Hierarchical Metal-Organic Flexible Cathode for High-Energy Sodium-Ion Batteries. *ChemSusChem* **2017,** *10*, 4704-4708.

(28) He, B.; Man, P.; Zhang, Q.; Fu, H.; Zhou, Z.; Li, C.; Li, Q.; Wei, L.; Yao, Y. All Binder-Free Electrodes for High-Performance Wearable Aqueous Rechargeable Sodium-Ion Batteries. *Nano-Micro Lett.* **2019,** *11*, 101.

(29) He, B.; Man, P.; Zhang, Q.; Wang, C.; Zhou, Z.; Li, C.; Wei, L.; Yao, Y. Conversion Synthesis of Self-Standing Potassium Zinc Hexacyanoferrate Arrays as Cathodes for High-Voltage Flexible Aqueous Rechargeable Sodium-Ion Batteries. *Small* **2019,** *15*, 1905115.

(30) Yun, J.; Schiegg, F. A.; Liang, Y.; Scieszka, D.; Garlyyev, B.; Kwiatkowski, A.; Wagner, T.; Bandarenka, A. S. Electrochemically Formed NaxMn[Mn(CN)$_6$] Thin Film Anodes Demonstrate Sodium Intercalation and Deintercalation at Extremely Negative Electrode Potentials in Aqueous Media. *ACS Appl. Energy Mater.* **2018,** *1*, 123-128.

(31) Meng, F.; Zhong, H.; Bao, D.; Yan, J.; Zhang, X. In Situ Coupling of Strung Co_4N and Intertwined N-C Fibers toward Free-Standing Bifunctional Cathode for Robust, Efficient, and

Flexible Zn-Air Batteries. *J. Am. Chem. Soc.y* **2016,** *138*, 10226-10231.
(32) Yang, F.; Xie, J.; Liu, X.; Wang, G.; Lu, X. Linker Defects Triggering Boosted Oxygen Reduction Activity of Co/Zn-ZIF Nanosheet Arrays for Rechargeable Zn-Air batteries. *Small* **2021,** *17*, 2007085.
(33) Yan, L.; Xu, Y.; Chen, P.; Zhang, S.; Jiang, H.; Yang, L.; Wang, Y.; Zhang, L.; Shen, J.; Zhao, X.; et al. A Freestanding 3D Heterostructure Film Stitched by MOF-Derived Carbon Nanotube Microsphere Superstructure and Reduced Graphene Oxide Sheets: A Superior Multifunctional Electrode for Overall Water Splitting and Zn-Air Batteries. *Adv. Mater.* **2020,** *32*, 2003313.
(34) Zhang, X.; Luo, J.; Lin, H.-F.; Tang, P.; Morante, J. R.; Arbiol, J.; Wan, K.; Mao, B.-W.; Liu, L.-M.; Fransaer, J. Tailor-Made Metal-Nitrogen-Carbon Bifunctional Electrocatalysts for Rechargeable Zn-air Batteries via Controllable MOF Units. *Energy Storage Mater.* **2019,** *17*, 46-61.
(35) Zang, W.; Sumboja, A.; Ma, Y.; Zhang, H.; Wu, Y.; Wu, S.; Wu, H.; Liu, Z.; Guan, C.; Wang, J.; et al. Single Co Atoms Anchored in Porous N-Doped Carbon for Efficient Zinc−Air Battery Cathodes. *ACS Catal.* **2018,** *8*, 8961-8969.
(36) Zhang, H.; Wang, T.; Sumboja, A.; Zang, W.; Xie, J.; Gao, D.; Pennycook, S. J.; Liu, Z.; Guan, C.; Wang, J. Integrated Hierarchical Carbon Flake Arrays with Hollow P-Doped $CoSe_2$ Nanoclusters as an Advanced Bifunctional Catalyst for Zn-Air Batteries. *Adv. Funct. Mater.* **2018,** *28*, 1804846.
(37) Zhang, Y.; Sun, H.; Qiu, Y.; Ji, X.; Ma, T.; Gao, F.; Ma, Z.; Zhang, B.; Hu, P. Multiwall Carbon Nanotube Encapsulated Co Grown on Vertically Oriented Graphene Modified Carbon Cloth as Bifunctional Electrocatalysts for Solid-State Zn-Air Battery. *Carbon* **2019,** *144*, 370-381.
(38) Ji, D.; Fan, L.; Li, L.; Peng, S.; Yu, D.; Song, J.; Ramakrishna, S.; Guo, S. Atomically Transition Metals on Self-Supported Porous Carbon Flake Arrays as Binder-Free Air Cathode for Wearable Zinc-Air Batteries. *Adv. Mater.* **2019,** *31*, 1808267.
(39) Zhu, C.; Ma, Y.; Zang, W.; Guan, C.; Liu, X.; Pennycook, S. J.; Wang, J.; Huang, W. Conformal Dispersed Cobalt Nanoparticles in Hollow Carbon Nanotube Arrays for Flexible Zn-Air and Al-Air Batteries. *Chem. Eng. J.* **2019,** *369*, 988-995.
(40) Hou, C.-C.; Zou, L.; Wang, Y.; Xu, Q. MOF-Mediated Fabrication of a Porous 3D Superstructure of Carbon Nanosheets Decorated with Ultrafine Cobalt Phosphide Nanoparticles for Efficient Electrocatalysis and Zinc-Air Batteries. *Angew. Chem. Int. Ed.* **2020,** *59*, 21360-21366.
(41) Ren, J.-T.; Yuan, G.-G.; Weng, C.-C.; Yuan, Z.-Y. Rationally Designed Co_3O_4-C Nanowire Arrays on Ni Foam Derived From Metal Organic Framework as Reversible Oxygen Evolution

Electrodes with Enhanced Performance for Zn-Air Batteries. *ACS Sustain. Chem. Eng.* **2018,** *6*, 707-718.

(42) Zhong, Y.; Pan, Z.; Wang, X.; Yang, J.; Qiu, Y.; Xu, S.; Lu, Y.; Huang, Q.; Li, W. Hierarchical Co_3O_4 Nano-Micro Arrays Featuring Superior Activity as Cathode in a Flexible and Rechargeable Zinc-Air Battery. *Adv. Sci.* **2019,** *6*, 1802243.

(43) Ji, D.; Fan, L.; Li, L.; Mao, N.; Qin, X.; Peng, S.; Ramakrishna, S. Hierarchical Catalytic Electrodes of Cobalt-Embedded Carbon Nanotube/Carbon Flakes Arrays for Flexible Solid-State Zinc-Air Batteries. *Carbon* **2019,** *142*, 379-387.

(44) Guan, C.; Sumboja, A.; Wu, H.; Ren, W.; Liu, X.; Zhang, H.; Liu, Z.; Cheng, C.; Pennycook, S. J.; Wang, J. Hollow Co_3O_4 Nanosphere Embedded in Carbon Arrays for Stable and Flexible Solid-State Zinc-Air Batteries. *Adv. Mater.* **2017,** *29*, 1704117.

(45) Wang, Z.; Ang, J.; Zhang, B.; Zhang, Y.; Ma, X. Y. D.; Yan, T.; Liu, J.; Che, B.; Huang, Y.; Lu, X. FeCo/FeCoNi/N-Doped Carbon Nanotubes Grafted Polyhedron-Derived Hybrid Fibers as Bifunctional Oxygen Electrocatalysts for Durable Rechargeable Zinc-Air Battery. *Appl. Catal. B: Environ.* **2019,** *254*, 26-36.

(46) Li, L.; Fu, L.; Wang, R.; Sun, J.; Li, X.; Fu, C.; Fang, L.; Zhang, W. Cobalt, Manganese Zeolitic-Imidazolate-Framework-Derived Co_3O_4/Mn_3O_4/CN_x Embedded in Carbon Nanofibers as an Efficient Bifunctional Electrocatalyst for Flexible Zn-Air Batteries. *Electrochim. Acta* **2020,** *344*, 136145.

(47) Li, B.; Igawa, K.; Chai, J.; Chen, Y.; Wang, Y.; Fam, D. W.; Tham, N. N.; An, T.; Konno, T.; Sng, A.; et al. String of Pyrolyzed ZIF-67 Particles on Carbon Fibers for High-Performance Electrocatalysis. *Energy Storage Mater.* **2020,** *25*, 137-144.

(48) Yu, P.; Wang, L.; Sun, F.; Xie, Y.; Liu, X.; Ma, J.; Wang, X.; Tian, C.; Li, J.; Fu, H. Co Nanoislands Rooted on Co-N-C Nanosheets as Efficient Oxygen Electrocatalyst for Zn-Air Batteries. *Adv. Mater.* **2019,** *31*, 1901666.

(49) Xu, Q.; Jiang, H.; Li, Y.; Liang, D.; Hu, Y.; Li, C. In-Situ Enriching Active Sites on Co-Doped Fe-Co_4N@N-C Nanosheet Array as Air Cathode for Flexible Rechargeable Zn-Air Batteries. *Appl. Catal. B: Environ.* **2019,** *256*, 117893.

(50) Amiinu, I. S.; Liu, X.; Pu, Z.; Li, W.; Li, Q.; Zhang, J.; Tang, H.; Zhang, H.; Mu, S. From 3D ZIF Nanocrystals to Co-Nx/C Nanorod Array Electrocatalysts for ORR, OER, and Zn-Air Batteries. *Adv. Funct. Mater.* **2018,** *28*, 1704638.

(51) Zou, H.; Li, G.; Duan, L.; Kou, Z.; Wang, J. In Situ Coupled Amorphous Cobalt Nitride with

Nitrogen-Doped Graphene Aerogel as a Trifunctional Electrocatalyst towards Zn-Air Battery Deriven Full Water Splitting. *Appl. Catal. B: Environ.* **2019,** *259*, 118100.
(52) Guan, C.; Sumboja, A.; Zang, W.; Qian, Y.; Zhang, H.; Liu, X.; Liu, Z.; Zhao, D.; Pennycook, S. J.; Wang, J. Decorating Co/CoN_x Nanoparticles in Nitrogen-Doped Carbon Nanoarrays for Flexible and Rechargeable Zinc-Air Batteries. *Energy Storage Mater.* **2019,** *16*, 243-250.
(53) Wang, X.; Liao, Z.; Fu, Y.; Neumann, C.; Turchanin, A.; Nam, G.; Zschech, E.; Cho, J.; Zhang, J.; Feng, X. Confined Growth of Porous Nitrogen-Doped Cobalt Oxide Nanoarrays as Bifunctional Oxygen Electrocatalysts for Rechargeable Zinc-Air Batteries. *Energy Storage Mater.s* **2020,** *26*, 157-164.
(54) Chen, Y.; Zhang, W.; Zhu, Z.; Zhang, L.; Yang, J.; Chen, H.; Zheng, B.; Li, S.; Zhang, W.; Wu, J.; et al. Co Nanoparticles Combined with Nitrogen-Doped Graphitic Carbon Anchored on Carbon Fibers as a Self-Standing Air Electrode for Flexible Zinc-Air Batteries. *J. Mater. Chem. A* **2020,** *8*, 7184-7191.
(55) Cai, J.-J.; Zhou, Q.-Y.; Liu, B.; Gong, X.-F.; Zhang, Y.-L.; Goh, K.; Gu, D.-M.; Zhao, L.; Sui, X.-L.; Wang, Z.-B. A Sponge-Templated Sandwich-Like Cobalt-Embedded Nitrogen-Doped Carbon Polyhedron/Graphene Composite as a Highly Efficient Catalyst for Zn-Air Batteries. *Nanoscale* **2020,** *12*, 973-982.
(56) Tang, Y.; Li, X.; Lv, H.; Xie, D.; Wang, W.; Zhi, C.; Li, H. Stabilized Co^{3+}/Co^{4+} Redox Pair in In Situ Produced CoSe2−x-Derived Cobalt Oxides for Alkaline Zn Batteries with 10 000-Cycle Lifespan and 1.9-V Voltage Plateau. *Adv. Energy Mater.* **2020,** *10*, 2000892.
(57) Chen, H.; Shen, Z.; Pan, Z.; Kou, Z.; Liu, X.; Zhang, H.; Gu, Q.; Guan, C.; Wang, J. Hierarchical Micro-Nano Sheet Arrays of Nickel-Cobalt Double Hydroxides for High-Rate Ni-Zn Batteries. *Adv. Sci.* **2019,** *6*, 1802002.
(58) Man, P.; He, B.; Zhang, Q.; Zhou, Z.; Li, C.; Li, Q.; Wei, L.; Yao, Y. A One-Dimensional Channel Self-Standing MOF Cathode for Ultrahigh-Energy-Density Flexible Ni-Zn Batteries. *J. Mater. Chem. A* **2019,** *7*, 27217-27224.
(59) Li, C.; Zhang, Q.; Sun, J.; Li, T.; E, S.; Zhu, Z.; He, B.; Zhou, Z.; Li, Q.; Yao, Y. High-Performance Quasi-Solid-State Flexible Aqueous Rechargeable Ag-Zn Battery Based on Metal-Organic Framework-Derived Ag Nanowires. *ACS Energy Lett.* **2018,** *3*, 2761-2768.
(60) Li, C.; Zhang, Q.; Li, T.; He, B.; Man, P.; Zhu, Z.; Zhou, Z.; Wei, L.; Zhang, K.; Hong, G.; et al. Nickel Metal-Organic Framework Nanosheets as Novel Binder-Ffree Cathode for Advanced

Fibrous Aqueous Rechargeable Ni-Zn Battery. *J. Mater. Chem. A* **2020,** *8*, 3262-3269.
(61) Liu, C.; Li, Q.; Sun, H.; Wang, Z.; Gong, W.; Cong, S.; Yao, Y.; Zhao, Z. MOF-Derived Vertically Stacked Mn_2O_3@C Flakes for Fiber-Shaped Zinc-Ion Batteries. *J. Mater. Chem. A* **2020,** *8*, 24031-24039.
(62) He, B.; Zhang, Q.; Man, P.; Zhou, Z.; Li, C.; Li, Q.; Xie, L.; Wang, X.; Pang, H.; Yao, Y. Self-Sacrificed Synthesis of Conductive Vanadium-Based Metal-Organic Framework Nanowire-Bundle Arrays as Binder-Free Cathodes for High-Rate and High-Energy-Density Wearable Zn-Ion Batteries. *Nano Energy* **2019,** *64*, 103935.
(63) Tan, Q.; Li, X.; Zhang, B.; Chen, X.; Tian, Y.; Wan, H.; Zhang, L.; Miao, L.; Wang, C.; Gan, Y.; et al. Valence Engineering via In Situ Carbon Reduction on Octahedron Sites Mn_3O_4 for Ultra-Long Cycle Life Aqueous Zn-Ion Battery. *Adv. Energy Mater.* **2020,** *10*, 2001050.
(64)Liu, X.; Zang, W.; Guan, C.; Zhang, L.; Qian, Y.; Elshahawy, A. M.; Zhao, D.; Pennycook, S. J.; Wang, J. Ni-Doped Cobalt-Cobalt Nitride Heterostructure Arrays for High-Power Supercapacitors. *ACS Energy Lett.* **2018,** *3*, 2462-2469.
(65) Kong, D.; Wang, Y.; Huang, S.; Hu, J.; Lim, Y. V.; Liu, B.; Fan, S.; Shi, Y.; Yang, H. Y. 3D Self-Branched Zinc-Cobalt Oxide@N-Doped Carbon Hollow Nanowall Arrays for High-Performance Asymmetric Supercapacitors and Oxygen Electrocatalysis. *Energy Storage Mater.* **2019,** *23*, 653-663.
(66) Liu, S.; Kang, L.; Zhang, J.; Jung, E.; Lee, S.; Jun, S. C. Structural Engineering and Surface Modification of MOF-Derived Cobalt-Based Hybrid Nanosheets for Flexible Solid-State Supercapacitors. *Energy Storage Mater.* **2020,** *32*, 167-177.
(67) Zhou, S.; Kong, X.; Zheng, B.; Huo, F.; Strømme, M.; Xu, C. Cellulose Nanofiber @ Conductive Metal-Organic Frameworks for High-Performance Flexible Supercapacitors. *ACS Nano* **2019,** *13*, 9578-9586.
(68) Li, W.-H.; Ding, K.; Tian, H.-R.; Yao, M.-S.; Nath, B.; Deng, W.-H.; Wang, Y.; Xu, G. Conductive Metal-Organic Framework Nanowire Array Electrodes for High-Performance Solid-State Supercapacitors. *Adv. Funct. Mater.* **2017,** *27*, 1702067.
(69) Chen, L.-F.; Lu, Y.; Yu, L.; Lou, X. W. Designed Formation of Hollow Particle-Based Nitrogen-Doped Carbon Nanofibers for High-Performance Supercapacitors. *Energy Environ. Sci.* **2017,** *10*, 1777-1783.
(70) Nagaraju, G.; Sekhar, S. C.; Ramulu, B.; Yu, J. S. High-Performance Hybrid Supercapacitors

Based on MOF-Derived Hollow Ternary Chalcogenides. *Energy Storage Mater.* **2021,** *35*, 750-760.

(71) Hou, R.; Miao, M.; Wang, Q.; Yue, T.; Liu, H.; Park, H. S.; Qi, K.; Xia, B. Y. Integrated Conductive Hybrid Architecture of Metal-Organic Framework Nanowire Array on Polypyrrole Membrane for All-Solid-State Flexible Supercapacitors. *Adv. Energy Mater.* **2020,** *10*, 1901892.

(72) Deng, T.; Lu, Y.; Zhang, W.; Sui, M.; Shi, X.; Wang, D.; Zheng, W. Inverted Design for High-Performance Supercapacitor Via $Co(OH)_2$-Derived Highly Oriented MOF Electrodes. *Adv. Energy Mater.* **2018,** *8*, 1702294.

(73) Zhou, Z.; Zhang, Q.; Sun, J.; He, B.; Guo, J.; Li, Q.; Li, C.; Xie, L.; Yao, Y. Metal-Organic Framework Derived Spindle-like Carbon Incorporated α-Fe_2O_3 Grown on Carbon Nanotube Fiber as Anodes for High-Performance Wearable Asymmetric Supercapacitors. *ACS Nano* **2018,** *12*, 9333-9341.

(74) Guan, C.; Liu, X.; Ren, W.; Li, X.; Cheng, C.; Wang, J. Rational Design of Metal-Organic Framework Derived Hollow $NiCo_2O_4$ Arrays for Flexible Supercapacitor and Electrocatalysis. *Adv. Energy Mater.* **2017,** *7*, 1602391.

(75) Li, Y.; Zhu, G.; Huang, H.; Xu, M.; Lu, T.; Pan, L. A N, S Dual Doping Strategy via Electrospinning to Prepare Hierarchically Porous Carbon Polyhedra Embedded Carbon Nanofibers for Flexible Supercapacitors. *J. Mater. Chem. A* **2019,** *7*, 9040-9050.

(76) Hong, M.; Zhou, C.; Xu, S.; Ye, X.; Yang, Z.; Zhang, L.; Zhou, Z.; Hu, N.; Zhang, Y. Bi-Metal Organic Framework Nanosheets Assembled on Nickel Wire Films for Volumetric-Energy-Dense Supercapacitors. *J. Power Sources* **2019,** *423*, 80-89.

(77) Li, G.; Cai, H.; Li, X.; Zhang, J.; Zhang, D.; Yang, Y.; Xiong, J. Construction of Hierarchical $NiCo_2O_4$@Ni-MOF Hybrid Arrays on Carbon Cloth as Superior Battery-Type Electrodes for Flexible Solid-State Hybrid Supercapacitors. *ACS Appl. Mater. Inter.* **2019,** *11*, 37675-37684.

(78) Lu, W.; Yuan, Z.; Xu, C.; Ning, J.; Zhong, Y.; Zhang, Z.; Hu, Y. Construction of Mesoporous Cu-Doped Co_9S_8 Rectangular Nanotube Arrays for High Energy Density All-Solid-State Asymmetric Supercapacitors. *J. Mater.Chem. A* **2019,** *7*, 5333-5343.

(79) Qi, K.; Hou, R.; Zaman, S.; Qiu, Y.; Xia, B. Y.; Duan, H. Construction of Metal-Organic Framework/Conductive Polymer Hybrid for All-Solid-State Fabric Supercapacitor. *ACS Appl. Mater. Inter.* **2018,** *10*, 18021-18028.

(80) Young, C.; Wang, J.; Kim, J.; Sugahara, Y.; Henzie, J.; Yamauchi, Y. Controlled Chemical Vapor Deposition for Synthesis of Nanowire Arrays of Metal-Organic Frameworks and Their

Thermal Conversion to Carbon/Metal Oxide Hybrid Materials. *Chem. Mater.* **2018,** *30*, 3379-3386.
(81) Liu, Y.; Wang, Y.; Shi, C.; Chen, Y.; Li, D.; He, Z.; Wang, C.; Guo, L.; Ma, J. Co-ZIF Derived Porous NiCo-LDH Nanosheets/N Doped Carbon Foam for High-Performance Supercapacitor. *Carbon* **2020,** *165*, 129-138.
(82) Wang, P.; Zhou, H.; Meng, C.; Wang, Z.; Akhtar, K.; Yuan, A. Cyanometallic Framework-Derived Hierarchical Co_3O_4-NiO/Graphene Foam as High-Performance Binder-Free Electrodes for Supercapacitors. *Chem. Eng. J.* **2019,** *369*, 57-63.
(83) Zhao, Y.; Dong, H.; He, X.; Yu, J.; Chen, R.; Liu, Q.; Liu, J.; Zhang, H.; Li, R.; Wang, J. Design of 2D Mesoporous Zn/Co-Based Metal-Organic Frameworks as a Flexible Electrode for Energy Storage and Conversion. *J. Power Sources* **2019,** *438*, 227057.
(84) Zhang, Y.; Chen, H.; Guan, C.; Wu, Y.; Yang, C.; Shen, Z.; Zou, Q. Energy-Saving Synthesis of MOF-Derived Hierarchical and Hollow $Co(VO_3)_2$-$Co(OH)_2$ Composite Leaf Arrays for Supercapacitor Electrode Materials. *ACS Appl. Mater. Inter.* **2018,** *10*, 18440-18444.
(85) Ji, D.; Zhou, H.; Zhang, J.; Dan, Y.; Yang, H.; Yuan, A. Facile Synthesis of a Metal-Organic Framework-Derived Mn_2O_3 Nanowire Coated Three-Dimensional Graphene Network for High-Performance Free-Standing Supercapacitor Electrodes. *J. Mater. Chem. A* **2016,** *4*, 8283-8290.
(86) Miao, C.; Xiao, X.; Gong, Y.; Zhu, K.; Cheng, K.; Ye, K.; Yan, J.; Cao, D.; Wang, G.; Xu, P. Facile Synthesis of Metal-Organic Framework-Derived $CoSe_2$ Nanoparticles Embedded in the N-Doped Carbon Nanosheet Array and Application for Supercapacitors. *ACS Appl. Mater. Inter.* **2020,** *12*, 9365-9375.
(87) Liu, Y.; Li, G.; Guo, Y.; Ying, Y.; Peng, X. Flexible and Binder-Free Hierarchical Porous Carbon Film for Supercapacitor Electrodes Derived from MOFs/CNT. *ACS Appl. Mater. Inter.* **2017,** *9*, 14043-14050.
(88) Cao, M.; Feng, Y.; Tian, R.; Chen, Q.; Chen, J.; Jia, M.; Yao, J. Free-Standing Porous Carbon Foam as the Ultralight and Flexible Supercapacitor Electrode. *Carbon* **2020,** *161*, 224-230.
(89) Zhang, C.; Xiao, J.; Lv, X.; Qian, L.; Yuan, S.; Wang, S.; Lei, P. Hierarchically Porous Co_3O_4/C Nanowire Arrays Derived from a Metal-Oganic Framework for High Performance Ssupercapacitors and the Oxygen Evolution Reaction. *J. Mater. Chem. A* **2016,** *4*, 16516-16523.
(90) Xin, N.; Liu, Y.; Niu, H.; Bai, H.; Shi, W. In-Situ Construction of Metal Organic Frameworks Derived Co/Zn-S Sandwiched Graphene Film as Free-Standing Electrodes for Ultra-High Energy Density Supercapacitors. *J. Power Sources* **2020,** *451*, 227772.

(91) Shinde, P. A.; Seo, Y.; Lee, S.; Kim, H.; Pham, Q. N.; Won, Y.; Chan Jun, S. Layered Manganese Metal-Organic Framework with High Specific and Areal Capacitance for Hybrid Supercapacitors. *Chem. Eng. J.* **2020,** *387*, 122982.

(92) Guo, D.; Song, X.; Tan, L.; Ma, H.; Pang, H.; Wang, X.; Zhang, L. Metal-Organic Framework Template-Directed Fabrication of Well-Aligned Pentagon-like Hollow Transition-Metal Sulfides as the Anode and Cathode for High-Performance Asymmetric Supercapacitors. *ACS Appl. Mater. Inter.* **2018,** *10*, 42621-42629.

(93) Yang, Q.; Liu, Y.; Yan, M.; Lei, Y.; Shi, W. MOF-Derived Hierarchical Nanosheet Arrays Constructed by Interconnected NiCo-Alloy@NiCo-Sulfide Core-Shell Nanoparticles for High-Performance Asymmetric Supercapacitors. *Chem. Eng.g J.* **2019,** *370*, 666-676.

(94) Xiong, D.; Gu, M.; Chen, C.; Lu, C.; Yi, F.-Y.; Ma, X. Rational Design of Bimetallic Metal-Organic Framework Composites and Their Derived Sulfides with Superior Electrochemical Performance to Remarkably Boost Oxygen Evolution and Supercapacitors. *Chem. Eng. J.* **2021,** *404*, 127111.

(95) Yang, Q.; Liu, Y.; Xiao, L.; Yan, M.; Bai, H.; Zhu, F.; Lei, Y.; Shi, W. Self-Templated Transformation of MOFs into Layered Double Hydroxide Nanoarrays with Selectively Formed Co_9S_8 for High-Performance Asymmetric Supercapacitors. *Chem. Eng. J.* **2018,** *354*, 716-726.

(96) Tao, Y.; Wu, Y.; Chen, H.; Chen, W.; Wang, J.; Tong, Y.; Pei, G.; Shen, Z.; Guan, C. Synthesis of Amorphous Hydroxyl-Rich Co_3O_4 for Flexible High-Rate Supercapacitor. *Chem. Eng. J.* **2020,** *396*, 125364.

(97) Li, Q.; Li, Y.; Zhao, J.; Zhao, S.; Zhou, J.; Chen, C.; Tao, K.; Liu, R.; Han, L. Ultrathin Nanosheet-Assembled Hollow Microplate $CoMoO_4$ Array Derived from Metal-Organic Framework for Supercapacitor with Ultrahigh Areal Capacitance. *J. Power Sources* **2019,** *430*, 51-59.

(98) Prasad Ojha, G.; Muthurasu, A.; Prasad Tiwari, A.; Pant, B.; Chhetri, K.; Mukhiya, T.; Dahal, B.; Lee, M.; Park, M.; Kim, H.-Y. Vapor Solid Phase Grown Hierarchical Cu_xO NWs Integrated MOFs-Derived CoS_2 Electrode for High-Performance Asymmetric Supercapacitors and the Oxygen Evolution Reaction. *Chem. Eng. J.* **2020,** *399*, 125532.

(99) Chu, W.; Hou, Y.; Liu, J.; Bai, X.; Gao, Y. f.; Cao, Z. Zn-Co Phosphide Porous Nanosheets Derived from Metal-Organic-Frameworks as Battery-Type Positive Electrodes for High-Performance Alkaline Supercapacitors. *Electrochim. Acta* **2020,** *364*, 137063.

(100) Wang, B.; Shang, J.; Guo, C.; Zhang, J.; Zhu, F.; Han, A.; Liu, J. A General Method to Ultrathin

Bimetal-MOF Nanosheets Arrays via In Situ Transformation of Layered Double Hydroxides Arrays. *Small* **2019,** *15*, 1804761.
(101) Lu, X.-F.; Gu, L.-F.; Wang, J.-W.; Wu, J.-X.; Liao, P.-Q.; Li, G.-R. Bimetal-Organic Framework Derived $CoFe_2O_4$/C Porous Hybrid Nanorod Arrays as High-Performance Electrocatalysts for Oxygen Evolution Reaction. *Adv. Mater.* **2017,** *29*, 1604437.
(102) Zhang, W.-D.; Yu, H.; Li, T.; Hu, Q.-T.; Gong, Y.; Zhang, D.-Y.; Liu, Y.; Fu, Q.-T.; Zhu, H.-Y.; Yan, X.; et al. Hierarchical Trimetallic Layered Double Hydroxide Nanosheets Derived from 2D Metal-Organic Frameworks for Enhanced Oxygen Evolution Reaction. *Appl. Catal. B: Environ.* **2020,** *264*, 118532.
(103) Zhou, J.; Dou, Y.; Zhou, A.; Shu, L.; Chen, Y.; Li, J.-R. Layered Metal-Organic Framework-Derived Metal Oxide/Carbon Nanosheet Arrays for Catalyzing the Oxygen Evolution Reaction. *ACS Energy Lett.* **2018,** *3*, 1655-1661.
(104) Ma, T. Y.; Dai, S.; Jaroniec, M.; Qiao, S. Z. Metal-Organic Framework Derived Hybrid Co_3O_4-Carbon Porous Nanowire Arrays as Reversible Oxygen Evolution Electrodes. *J. Am. Chem. Soc.* **2014,** *136*, 13925-13931.
(105) Li, F.-L.; Shao, Q.; Huang, X.; Lang, J.-P. Nanoscale Trimetallic Metal-Organic Frameworks Enable Efficient Oxygen Evolution Electrocatalysis. *Angew. Chem. Int. Ed.* **2018,** *57*, 1888-1892.
(106) Sun, F.; Wang, G.; Ding, Y.; Wang, C.; Yuan, B.; Lin, Y. NiFe-Based Metal-Organic Framework Nanosheets Directly Supported on Nickel Foam Acting as Robust Electrodes for Electrochemical Oxygen Evolution Reaction. *Adv. Energy Mater.* **2018,** *8*, 1800584.
(107) Xi, W.; Yan, G.; Lang, Z.; Ma, Y.; Tan, H.; Zhu, H.; Wang, Y.; Li, Y. Oxygen-Doped Nickel Iron Phosphide Nanocube Arrays Grown on Ni Foam for Oxygen Evolution Electrocatalysis. *Small* **2018,** *14*, 1802204.
(108) Huang, L.; Gao, G.; Zhang, H.; Chen, J.; Fang, Y.; Dong, S. Self-dissociation-assembly of ultrathin metal-organic framework nanosheet arrays for efficient oxygen evolution. *Nano Energy* **2020,** *68*, 104296.
(109) Wang, L.; Wu, Y.; Cao, R.; Ren, L.; Chen, M.; Feng, X.; Zhou, J.; Wang, B. Fe/Ni Metal-Organic Frameworks and Their Binder-Free Thin Films for Efficient Oxygen Evolution with Low Overpotential. *ACS Appl. Mater. Inter.* **2016,** *8*, 16736-16743.
(110) Dong, Q.; Wang, Q.; Dai, Z.; Qiu, H.; Dong, X. MOF-Derived Zn-Doped $CoSe_2$ as an Efficient and Stable Free-Standing Catalyst for Oxygen Evolution Reaction. *ACS Appl. Mater. Inter.* **2016,** *8*,

26902-26907.
(111) He, P.; Xie, Y.; Dou, Y.; Zhou, J.; Zhou, A.; Wei, X.; Li, J.-R. Partial Sulfurization of a 2D MOF Array for Highly Efficient Oxygen Evolution Reaction. *ACS Appl. Mater. Inter.* **2019,** *11*, 41595-41601.
(112) Cao, L.-M.; Hu, Y.-W.; Zhong, D.-C.; Lu, T.-B. Template-Directed Growth of Bimetallic Prussian Blue-Analogue Nanosheet Arrays and Their Derived Porous Metal Oxides for Oxygen Evolution Reaction. *ChemSusChem* **2018,** *11*, 3708-3713.
(113) Wang, X.; He, J.; Yu, B.; Sun, B.; Yang, D.; Zhang, X.; Zhang, Q.; Zhang, W.; Gu, L.; Chen, Y. $CoSe_2$ Nanoparticles Embedded MOF-Derived Co-N-C Nanoflake Arrays as Efficient and Stable Electrocatalyst for Hydrogen Evolution Reaction. *Appl. Catal. B: Environ.* **2019,** *258*, 117996.
(114) Wang, X.; Chen, Y.; Yu, B.; Wang, Z.; Wang, H.; Sun, B.; Li, W.; Yang, D.; Zhang, W. Hierarchically Porous W-Doped CoP Nanoflake Arrays as Highly Efficient and Stable Electrocatalyst for pH-Universal Hydrogen Evolution. *Small* **2019,** *15*, 1902613.
(115) Weng, B.; Grice, C. R.; Meng, W.; Guan, L.; Xu, F.; Yu, Y.; Wang, C.; Zhao, D.; Yan, Y. Metal-Organic Framework-Derived CoWP@C Composite Nanowire Electrocatalyst for Efficient Water Splitting. *ACS Energy Lett.* **2018,** *3*, 1434-1442.
(116) Xu, Y.; Yu, S.; Ren, T.; Liu, S.; Wang, Z.; Li, X.; Wang, L.; Wang, H. Hydrophilic/Aerophobic Hydrogen-Evolving Electrode: NiRu-Based Metal-Organic Framework Nanosheets In Situ Grown on Conductive Substrates. *ACS Appl. Mater. Inter.* **2020,** *12*, 34728-34735.
(117) Li, Y.; Zhang, B.; Wang, W.; Shi, X.; Zhang, J.; Wang, R.; He, B.; Wang, Q.; Jiang, J.; Gong, Y.; et al. Selective-Etching of MOF toward Hierarchical Porous Mo-Doped CoP/N-Doped Carbon Nanosheet Arrays for Efficient Hydrogen Evolution at All pH Values. *Chem. Eng. J.* **2021,** *405*, 126981.
(118) Xu, H.; Fei, B.; Cai, G.; Ha, Y.; Liu, J.; Jia, H.; Zhang, J.; Liu, M.; Wu, R. Boronization-Induced Ultrathin 2D Nanosheets with Abundant Crystalline-Amorphous Phase Boundary Supported on Nickel Foam toward Efficient Water Splitting. *Adv. Energy Mater.* **2020,** *10*, 1902714.
(119) Cao, L.-M.; Hu, Y.-W.; Tang, S.-F.; Iljin, A.; Wang, J.-W.; Zhang, Z.-M.; Lu, T.-B. Fe-CoP Electrocatalyst Derived from a Bimetallic Prussian Blue Analogue for Large-Current-Density Oxygen Evolution and Overall Water Splitting. *Adv. Sci.* **2018,** *5*, 1800949.
(120) Guan, C.; Xiao, W.; Wu, H.; Liu, X.; Zang, W.; Zhang, H.; Ding, J.; Feng, Y. P.; Pennycook, S. J.; Wang, J. Hollow Mo-Doped CoP Nanoarrays for Efficient Overall Water Splitting. *Nano*

Energy **2018,** *48*, 73-80.
(121) Zhao, M.; Li, W.; Li, J.; Hu, W.; Li, C. M. Strong Electronic Interaction Enhanced Electrocatalysis of Metal Sulfide Clusters Embedded Metal-Organic Framework Ultrathin Nanosheets toward Highly Efficient Overall Water Splitting. *Adv. Sci.* **2020,** *7*, 2001965.
(122) Cai, G.; Zhang, W.; Jiao, L.; Yu, S.-H.; Jiang, H.-L. Template-Directed Growth of Well-Aligned MOF Arrays and Derived Self-Supporting Electrodes for Water Splitting. *Chem* **2017,** *2*, 791-802.
(123) Duan, J.; Chen, S.; Zhao, C. Ultrathin Metal-Organic Framework Array for Efficient Electrocatalytic Water Splitting. *Nat. Commun.* **2017,** *8*, 15341.
(124) Chen, Z.; Fei, B.; Hou, M.; Yan, X.; Chen, M.; Qing, H.; Wu, R. Ultrathin Prussian Blue Analogue Nanosheet Arrays with Open Bimetal Centers for Efficient Overall Water Splitting. *Nano Energy* **2020,** *68*, 104371.
(125) Li, Z.; Cui, J.; Liu, Y.; Li, J.; Liu, K.; Shao, M. Electrosynthesis of Well-Defined Metal-Organic Framework Films and the Carbon Nanotube Network Derived from Them toward Electrocatalytic Applications. *ACS Appl. Mater. Inter.* **2018,** *10*, 34494-34501.
(126) Zhou, W.; Lu, X.-F.; Chen, J.-J.; Zhou, T.; Liao, P.-Q.; Wu, M.; Li, G.-R. Hierarchical Porous Prism Arrays Composed of Hybrid Ni-NiO-Carbon as Highly Efficient Electrocatalysts for Overall Water Splitting. *ACS Appl. Mater. Inter.* **2018,** *10*, 38906-38914.
(127) Liu, J.; Gao, Y.; Tang, X.; Zhan, K.; Zhao, B.; Xia, B. Y.; Yan, Y. Metal-Organic Framework-Derived Hierarchical Ultrathin CoP Nanosheets for Overall Water Splitting. *J. Mater. Chem. A* **2020,** *8*, 19254-19261.
(128) Guan, C.; Wu, H.; Ren, W.; Yang, C.; Liu, X.; Ouyang, X.; Song, Z.; Zhang, Y.; Pennycook, S. J.; Cheng, C.; et al. Metal-Organic Framework-Derived Integrated Nanoarrays for Overall Water Splitting. *J. Mater. Chem. A* **2018,** *6*, 9009-9018.
(129) Xu, S.; Du, J.; Li, J.; Sun, L.; Li, F. Nickel-Selenide Templated Binary Metal-Qrganic Frameworks for Efficient Water Oxidation. *J. Mater. Chem. A* **2020,** *8*, 16908-16912.
(130) Zhang, H.; Liu, Y.; Wu, H.; Zhou, W.; Kou, Z.; Pennycook, S. J.; Xie, J.; Guan, C.; Wang, J. Open Hollow Co-Pt Clusters Embedded in Carbon Nanoflake Arrays for Highly Efficient Alkaline Water Splitting. *J. Mater. Chem. A* **2018,** *6*, 20214-20223.
(131) Xiang, R.; Duan, Y.; Tong, C.; Peng, L.; Wang, J.; Shah, S. S. A.; Najam, T.; Huang, X.; Wei, Z. Self-Standing FeCo Prussian Blue Analogue Derived FeCo/C and FeCoP/C Nanosheet Arrays for

Cost-Effective Electrocatalytic Water Splitting. *Electrochim. Acta* **2019,** *302*, 45-55.

(132) Zhou, Q.; Wang, J.; Guo, F.; Li, H.; Zhou, M.; Qian, J.; Li, T.-T.; Zheng, Y.-Q. Self-Supported Bimetallic Phosphide-Carbon Nanostructures Derived from Metal-Organic Frameworks as Bifunctional Catalysts for Highly Efficient Water Splitting. *Electrochim. Acta* **2019,** *318*, 244-251.

(133) Ye, W.; Yang, Y.; Fang, X.; Arif, M.; Chen, X.; Yan, D. 2D Cocrystallized Metal-Organic Nanosheet Array as an Efficient and Stable Bifunctional Electrocatalyst for Overall Water Splitting. *ACS Sustain. Chem. Eng.* **2019,** *7*, 18085-18092.
